# Photoluminescence of Pentavalent Uranyl Amide Complexes

**DOI:** 10.1021/jacs.1c05184

**Published:** 2021-08-13

**Authors:** Fabrizio Ortu, Simon Randall, David J. Moulding, Adam W. Woodward, Andrew Kerridge, Karsten Meyer, Henry S. La Pierre, Louise S. Natrajan

**Affiliations:** † Centre for Radiochemistry Resesarch, Department of Chemistry, School of Natural Sciences, The University of Manchester, Oxford Road, Manchester M13 9PL, U.K.; ‡ School of Chemistry, University of Leicester, University Road, Leicester LE1 7RH, U.K.; § Photon Science Institute, The University of Manchester, Oxford Road, Manchester M13 9PL, U.K.; ∥ Department of Chemistry, Lancaster University, Lancaster LA1 4YB, U.K.; ⊥ Friedrich-Alexander-University Erlangen-Nürnberg (FAU), Department of Chemistry and Pharmacy, Inorganic Chemistry, Egerlandstr. 1, 91058 Erlangen, Germany; # School of Chemistry and Biochemistry, Georgia Institute of Technology, Atlanta, Georgia 30332-0400, United States; ¶ Nuclear and Radiological Engineering and Medical Physics Program, School of Mechanical Engineering, Georgia Institute of Technology, Atlanta, Georgia 30332-0400, United States

## Abstract

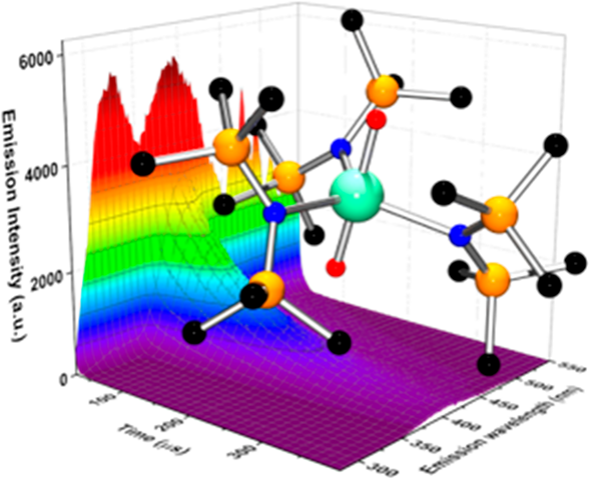

Pentavalent uranyl species are crucial intermediates in transformations that play a key role for the nuclear industry and have recently been demonstrated to persist in reducing biotic and abiotic aqueous environments. However, due to the inherent instability of pentavalent uranyl, little is known about its electronic structure. Herein, we report the synthesis and characterization of a series of monomeric and dimeric, pentavalent uranyl amide complexes. These synthetic efforts enable the acquisition of emission spectra of well-defined pentavalent uranyl complexes using photoluminescence techniques, which establish a unique signature to characterize its electronic structure and, potentially, its role in biological and engineered environments via emission spectroscopy.

## Introduction

The aqueous chemistry of uranium is dominated by the uranyl dication, [U^VI^O_2_]^2+^.^1−4^ Therefore, understanding its physicochemical properties is vital to the successful implementation of adequate nuclear waste management strategies.^5−9^ Despite its inherent stability, uranyl can undergo a number of transformations in the environment, the cornerstone of which is the reduction of soluble uranyl(VI) to insoluble U^4+^.^10^ This transformation has been postulated to proceed via a disproportionation mechanism, which involves the formation of transient uranyl(V) species, {[U^V^O_2_]^+^}_*n*_ (*n* = 1 or 2).^11,12^ The detection of this transient species in the environment and in biological systems remains a challenging goal due to its redox instability and lability.^13^

In aqueous media, [U^V^O_2_]^+^ has been observed in acidic solutions (pH 2–3)^14^ and in concentrated carbonate solutions.^15^ Molecular [U^V^O_2_]^+^ compounds have also been stabilized by operating in the rigorous exclusion of air and moisture.^9,16,17^ Quite remarkably, they have recently been isolated and studied in aqueous media.^18−20^ Since 2003,^21^ there have been a number of reports on the structural and chemical properties of the once elusive [U^V^O_2_]^+^ cation.^9,16,17,22−29^ Nevertheless, there is a remarkable paucity of information regarding the electronic structure and photophysical properties of [U^V^O_2_]^+^.^30^ This is in contrast to the extensive studies of the [U^VI^O_2_]^2+^ dication. In prior seminal reports on the emission spectra of [U^V^O_2_]^+^ species,^31,32^ [U^V^O_2_]^+^ was generated in situ using photolytic or electrolytic reduction and analysis required deconvolution of the luminescence spectra. Very recently, Mazzanti and co-workers reported the emission spectra of a water-stable dipicolinate [U^V^O_2_]^+^ complex.^20^ However, the photophysical properties of the [U^V^O_2_]^+^ cation remain unassigned. We have previously shown that the application of photoluminescence techniques to [U^VI^O_2_]^2+^ can provide detailed information regarding the behavior of uranyl, particularly its coordination, speciation, and nuclearity including the existence of “cation–cation” interactions (CCIs).^33^ Therefore, the application of this approach to discrete molecular [U^V^O_2_]^+^ compounds can offer a essential diagnostic tools for understanding its physicochemical properties.^13^ Herein, we report the synthesis and structural authentication of a series of pentavalent uranyl complexes and the detailed characterization of their photoluminescence properties.

## Experimental Section

### General Methods

***Caution!**^238^U (*t*_1/2_ = 4.47 × 10^9^ years) is a weak α-emitter; therefore, all manipulations should be performed in suitable laboratories that have been designated for radiochemical use, and α-counting equipment should be available.* All manipulations were carried out using standard Schlenk techniques or in an Inert Purelab HE 2GB glovebox. Solvents were dried by refluxing over potassium and were distilled and degassed before use. All solvents were stored over potassium mirrors (with the exception of THF and DME, which were stored over activated 4 Å molecular sieves). [K(THF)_3_][UO_2_(N″)_3_] (**1**), [K(crypt)][UO_2_(N″)_3_] (**2-crypt**), [UO_2_(N″)_2_(THF)_2_] (**4**) were prepared following literature procedures.^34^^1^H, ^13^C{^1^H}, and ^29^Si{^1^H} NMR spectra were recorded at 298 K on a Bruker Avance 400 spectrometer operating at 400.2, 100.6, and 79.5 MHz, respectively; chemical shifts are quoted in ppm and are relative to TMS. FTIR spectra were recorded as Nujol mulls in KBr discs using a Shimadzu IRAffinity-1S spectrometer. Raman spectra were recorded using a XploRAPLUS Horiba Scientific spectrometer. Electronic absorption spectra were recorded in sealed 10 mm path length cuvettes using a Shimadzu UV-2600 spectrometer.

### Synthesis

#### [K(18-crown-6)][UO_2_(N″)_3_] (**2–18C6**)

A Schlenk flask fitted with magnetic stir bar was charged with **4** (1.225 g, 1.7 mmol), KN″ (0.356 g, 1.8 mmol) and 18-crown-6 (0.467 g, 1.8 mmol); the flask was cooled to −50 °C, THF (15 mL) was added and the orange solution stirred for 16 h at room temperature. Volatiles were removed under reduced pressure and the solid washed with hexane (10 mL), then dried in vacuo for 2 h at room temperature, affording **2–18C6** as an orange powder. Yield: 1.416 g, 1.3 mmol, 80%. Anal. Calc’d (%) for C_30_H_78_KN_3_O_8_Si_6_U: C 34.17, H 7.45, N 3.98; Found: C 34.14, H 7.44, N 3.61. ^1^H NMR (pyridine-*d*_6_, 298 K, 400 MHz) δ 0.79 (s, 36H, Si(C*H*_3_)_3_), 3.50 (s, 24H, 18-crown-6–C*H*_2_). ^13^C{^1^H} NMR (pyridine-*d*_6_, 298 K, 100 MHz) δ 6.86 (Si(*C*H_3_)_3_), 70.95 (18-crown-6-*C*H_2_). ^29^Si{^1^H} (pyridine-*d*_6_, 298 K, 79.5 MHz) δ −8.52 (*Si*(CH_3_)_3_). FTIR (ATR microcrystalline) ν̃ 2945 (w), 2892 (w), 1352 (w), 1233 (m), 1103 (s), 959 (vs, U=O_asym_), 833 (vs), 770 (w), 689 (w), 658 (s), 607 (s) cm^–1^. FTIR (ATR) ν̃ 2971 (br, m), 2859 (br, m), 1454 (w), 1352 (w), 1233 (w), 1107 (m), 1066 (vs), 961 (s, U=O_asym_), 907 (w), 834 (vs), 768 (w), 668 (w), 661 (m), 605 (m). Raman (Solid, 638 nm, 100%) ν̃ 2904 (br, 799), 1474 (640), 1278 (600), 1247 (479), 1135 (492), 802 (2257) (U=O_sym_), 280 (1256) cm^–1^ (counts). UV/vis (0.05 mM, THF) λ_max_ (ε/mol^–1^ cm^–1^): 497 (270), 370 (2632), 324 (4018), 230 (5262), 212 (5026) nm. 2–18C6-THF: Crystals were grown form a THF solution layered with hexane and stored at −25 °C several days. 2–18C6-Tol: Crystals were grown from cooling of a boiling toluene solution. 2–18C6-CHCl_3_: Crystals were grown from a saturated solution in CHCl_3_ at room temperature.

#### [K(2.2.2-cryptand)][UO_2_N″_3_] (**2-crypt**)

A Schlenk flask fitted with magnetic stir bar was charged with **1** (1.701 g, 1.7 mmol) and 2.2.2-cryptand (0.636 g, 1.7 mmol); DME (20 mL) was added and the reaction stirred for 4 h. The deep red solution was concentrated to 5 mL and stored at −30 °C, affording **2-crypt** as bright red crystals (1.595 g, 1.4 mmol, 80%). Spectroscopic data matched that previously reported in the literature.^34^

#### [K(18-crown-6)(DME)]_2_[UO_2_(N″)_3_] (**3–18C6**)

A Schlenk flask with a stirrer bar was charged with **2–18C6** (0.836 g, 0.79 mmol). Additionally, a separate flask was charged with a mixture of 18-crown-6 (0.220 g, 0.83 mmol) and KC_8_ (0.109 g, 0.80 mmol). Both flasks were cooled to −50 °C and DME (5 mL each) was added. Both mixtures were stirred for 5 min, then the slurry of 18-crown-6 and KC_8_ was added quickly to the solution of **1–18C6**. More DME (2 mL) was added in order to collect the rest of the KC_8_ slurry and complete the addition. The reaction was allowed to stir at −50 °C for 5 min and then left to settle for 2 min. The suspension was then filtered into a precooled Schlenk flask (−50 °C) affording an emerald green solution. Subsequent concentration of the mother liqueur at −30 °C (ca. 5 mL) followed by layering with pentane (7 mL) and storage at −30 °C led to emerald green crystals of **3–18C6**. Yield: 0.344 g, 0.21 mmol, 27%. ^1^H NMR (tetrahydrofuran-*d*_8_, 298 K) δ −8.64 (br, ν1/2 = 52 Hz, 18H, Si(C*H*_*3*_)_3_), −4.94 (br, ν1/2 = 340 Hz, 9H, Si(C*H*_*3*_)_3_), 3.28 (s, ν1/2 = 3 Hz, 12H, DME), 3.44 (s, ν1/2 = 4 Hz, 8H, DME), 6.44 (br, ν1/2 = 44 Hz, 24H, crown–C*H*_2_). ^13^C{^1^H} NMR (tetrahydrofuran-*d*_8_, 298 K) δ 6.29 (s, Si(*C*H_3_)_3_), 6.50 (s, Si(*C*H_3_)_3_), 58.95 (s, DME), 72.83 (s, DME), 73.35 (br, 18-crown-6-*C*H_2_). ^29^Si{^1^H} NMR (*d*_8_-THF, 298 K) δ −10.87 (*Si*(CH_3_)_3_). μ_eff_ (Evans method, 298 K, tetrahydrofuran-*d*_8_) 2.68 μ_B_. FTIR (ATR microcrystalline) ν̃ 2889 (br, m), 1452 (w), 1352 (m), 1233 (m), 1103 (vs), 1025 (s), 958 (s), 873 (m, U=O_(yl) asym_), 821 (vs), 758 (s), 695 (w), 650 (s), 591 (w) cm^–1^. Raman (solid, 638 nm, 10%) (Smoothed) ν̃ 2893 (br, s), 1475 (w), 1282 (w), 1142 (w), 837 (m), 727 (br, s, U=O_(yl) sym_), 609 (w), 415 (br, m), 282 (br, w). Anal. Calc’d (%) for C_46_H_112_K_2_N_3_O_16_Si_6_U·C_4_H_10_O_2_: C 38.15, H 7.80, N 2.90; Found: C 37.92, H 7.83, N 2.57. UV/vis (2.65 mM, THF) λ_max_ (ε/mol^–1^ cm^–1^): 773 (45), 650 (60), 622-shoulder (58), 567-shoulder (71) 496 (150) nm.

#### [K(2.2.2-cryptand)]_2_[UO_2_(N″)_3_] (**3-crypt**)

A Schlenk flask fitted with a stirrer bar was charged with **2-crypt** (1.084 g, 0.9 mmol); a separate flask was charged with a mixture of 2.2.2-cryptand (0.350 g, 0.9 mmol) and KC_8_ (0.126 g, 0.9 mmol). Both flasks were cooled to −50 °C and DME (5 and 10 mL respectively) was added. The slurry of 2.2.2-cryptand and KC_8_ was added quickly to the solution of 2-crypt; more DME (5 mL) was added in order to collect the rest of the KC_8_ slurry and complete the addition. The reaction was allowed to stir at −50 °C and then filtered in a precooled Schlenk flask (−50 °C), affording a bright green solution. The solution was stored at −25 °C, affording **3-crypt** as emerald green crystals (0.791 g, 0.5 mmol, 51%). ^1^H NMR (tetrahydrofuran-*d*_8_, 298 K, 400 MHz) δ −8.47 (br, ν_1/2_ = 60 Hz, 5H, N(SiC*H*_3_)_2_), −4.75 (br, ν_1/2_ = 324 Hz, 36H, N(SiC*H*_3_)_2_), −0.09 (s, ν_1/2_ = 4 Hz, 9H, N(SiC*H*_3_)_2_), 0.33 (s, ν_1/2_ = 4 Hz, 4H, N(SiC*H*_3_)_2_), 6.72 (br, ν_1/2_ = 24 Hz, 24H, crypt–C*H*_2_), 8.17 (br, ν_1/2_ = 36 Hz, 24H, crypt–C*H*_2_), 8.71 (br, ν_1/2_ = 40 Hz, 24H, crypt–C*H*_2_) ppm. ^13^C{^1^H} NMR (tetrahydrofuran-*d*_8_, 298 K, 100 MHz) δ 6.39 (N(Si*C*H_3_)_2_), 7.09 (N(Si*C*H_3_)_2_), 58.66 (crypt-*C*H_2_), 72.64 (crypt-*C*H_2_), 75.90 (crypt-*C*H_2_) ppm. ^29^Si{^1^H} (tetrahydrofuran-*d*_8_, 298 K, 79.5 MHz) δ −103.12 ppm. μ_eff_ (Evans method, 298 K, tetrahydrofuran-*d*_8_) 2.47 μ_B._ Anal. Calcd (%) for C_54_H_126_K_2_N_7_O_14_Si_6_U: C 40.99, H 8.03, N 6.20; found: C 40.92, H 8.31, N 6.09. UV/vis (3.1 mM, DME) λ_max_ (*ε*/mol^–1^ cm^–1^): 649 (136), 622 (57), 490 (37) nm. FTIR (KBr disc in Nujol mull) ν̃ 1722 (w), 1622 (w), 1362 (s),1296 (s), 1261 (m), 1236 (m), 1223 (m), 1175 (w), 1101 (br s w/shoulder), 1030 (br s), 949 (m), 932 (w), 872 (m, U=O_asym_), 816 (br s), 762 (m w/shoulder), 698 (w), 662 (m), 648 (m), 582–360 (br s) cm^–1^. Raman (50 mM in THF) ν̃ 753(544), 829(705) cm^–1^ (counts), (U=O_(yl) sym_); Raman (solid, 532 nm, 100%) ν̃ 164(127), 192(158), 488(636), 709(279), 790(126), 1416(74) cm^–1^ (counts).

#### [K(18-crown-6)(DME)]_2_[{UO(μ-O)(N″)_2_}_2_] (**5**) and [K(18-crown-6)(DME)]_2_[{UO_2_(μ-O_2_)(N″)_2_}_2_] (**6**)

In the glovebox, to a vial containing a magnetic stirrer bar was added **4** (250 mg, 0.34 mmol) and 10 mL of 1:1 THF:hexane. The mixture was stirred and the dark orange solution transferred to a–35 °C freezer. KC_8_(46 mg, 0.34 mmol) and 2.2.2-cryptand (0.128 mg, 0.34 mmol) were weighed into separate vials, the crypt dissolved in 4 mL of THF and the vials stored at −35 °C. After 30 min, the THF/cryptand solution was transferred to the KC_8_ and the resulting slurry added to the uranyl solution dropwise over 2 min with vigorous stirring. Washings were transferred with an additional 2 mL of cold THF and the mixture stirred for a further minute before being moved to the −35 °C freezer for 1 min. The slurry was stirred and filtered through a fine porosity frit and the brown filtrate concentrated to ca. 3 mL under reduced pressure and cooled to −35 °C. The resultant brown solid was collected and washed with cold THF/hexane (∼1:5, 50 mL) and dried in vacuo. Crystals of **5** were obtained from a concentrated solution in DME stored at −35 °C (Yield: 132 mg, 0.13 mmol, 38%). When the reaction was attempted using Schlenk line techniques, small crops of [K(18-crown-6)(DME)]_2_[{UO_2_(μ-O_2_)(N″)_2_}_2_] (**6**) were also isolated and characterized via single crystal XRD. The high thermal instability of **5** in solution precluded spectroscopic characterization. Anal. Calc’d (%) for C_60_H_144_K_2_N_8_O_16_Si_8_U_2_: C 35.80, H 7.21, N 5.57; found: C 35.22, H 7.12, N 5.07.

#### [{U(μ-O)_2_(N″)_2_(μ-Cl)}{K(18-crown-6)}_2_] (**7**)

A Schlenk flask with a stirrer bar was charged with **4** (0.735 g, 1 mmol). Additionally, a separate flask was charged with a mixture of 18-crown-6 (0.529 g, 2 mmol), KC_8_ (0.137 g, 1 mmol) and KCl (0.087 g, 1.2 mmol). Both flasks were cooled to −50 °C and DME (5 mL each) was added. Both mixtures were allowed to stir for 5 min, at which point the slurry of 2.2.2-cryptand and KC_8_ was added quickly to the solution of **4**. More DME (ca. 5 mL) was added in order to collect the rest of the KC_8_ slurry and complete the addition. The reaction was allowed to stir at −50 °C for 5 min at which point stirring was stopped and the solution left to settle for 2 min. The solution was then filtered into a precooled Schlenk flask (−50 °C) affording an amber-brown solution. Subsequent concentration of the mother liquor to ca. 5 mL followed by layering with pentane (7 mL) and storage at −25 °C lead to amber-brown crystals of **7**. Yield: 0.378 g, 0.38 mmol, 38%). ^1^H NMR (tetrahydrofuran-*d*_8_, 298 K) δ −8.52 (br, ν_1/2_ = 55 Hz, Si(C*H*_3_)_3_, 5.18 (br, ν_1/2_ = 51 Hz, crown–C*H*_2_). ^13^C{^1^H} NMR (tetrahydrofuran-*d*_8_, 298 K) δ 6.24 (Si(*C*H_3_)_3_), 72.15 (crown-*C*H_2_). ^29^Si{^1^H} NMR signal not observed. μ_eff_ (Evans method, 298 K, tetrahydrofuran-*d*_8_) 2.44 μ_B_. Anal. Calc’d (%) for C_36_H_84_ClK_2_N_2_O_14_Si_4_U: C 35.07, H 6.87, N 2.27; Found: C 35.44, H 7.08, N 2.17. FTIR (ATR microcrystalline) ν̃ 2865 (br m), 1452 (w), 1352 (m), 1235 (m), 1103 (vs), 958 (s), 861 (m-shoulder, U=O_(yl) asym_), 821 (s), 748 (w), 644 (w), 593 (w) cm^–1^. Raman (solid, 638 nm, 10%, smoothed) ν̃ 2900 (br, s), 1472(m), 1274(m), 1140 (w), 869 (m), 784 (br s) (U=O_(yl) sym_), 434 (br m), 277 (m) cm^–1^. UV/vis (5.63 mM, THF) λ_max_ (*ε*/mol^–1^ cm^–1^): 841 (54), 655 (105), 495 (250) nm.

## Results and Discussion

### Synthesis and NMR Characterization

**Scheme 1:**
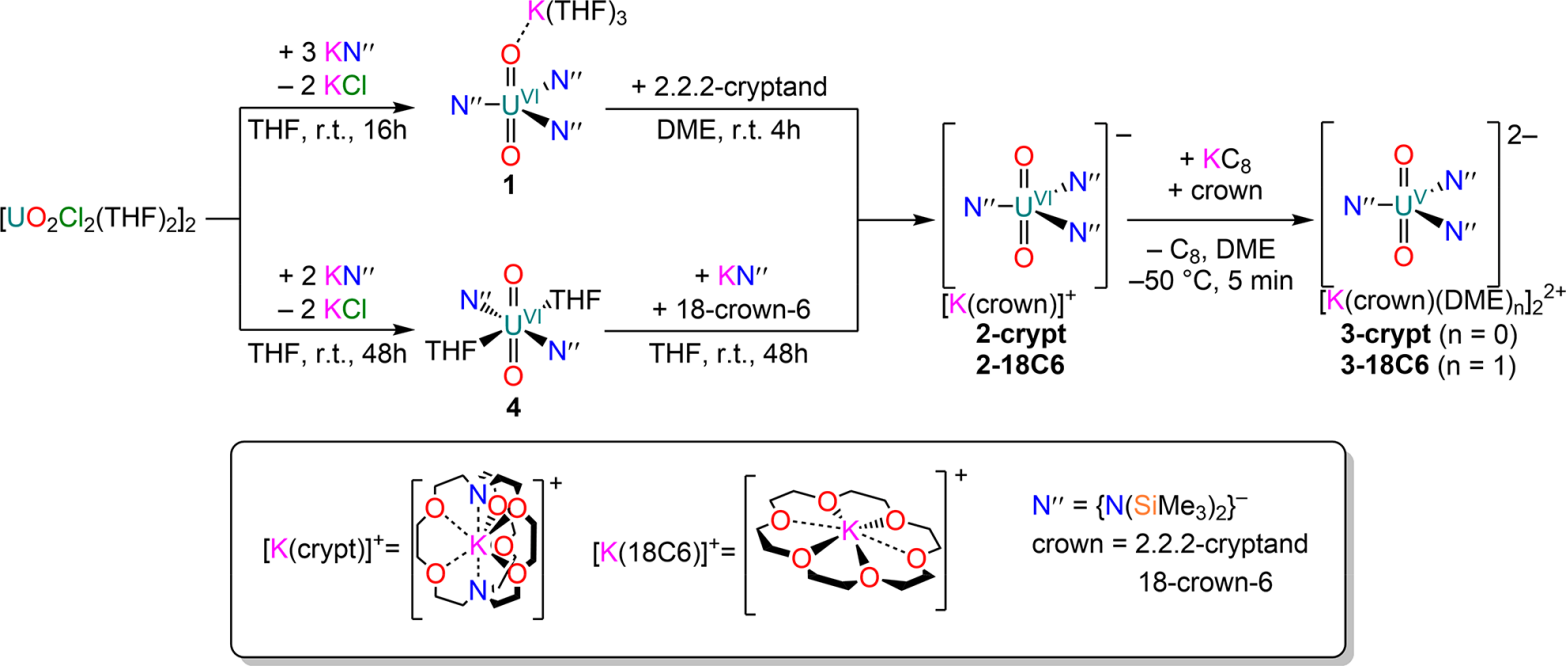
Synthesis of Uranyl(VI) Tris-amides, **2-crypt** and **2-18C6**, and Reduction to Monomeric Uranyl(V) Complexes **3-crypt** and **3-18C6**

**Scheme 2:**
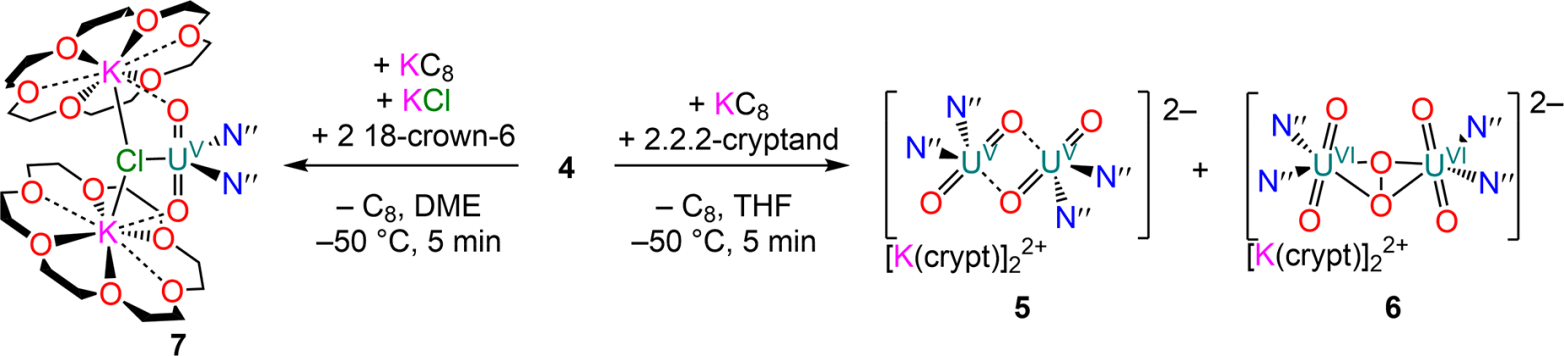
Reduction of Uranyl(VI) Bis-amide **4** and to Dimeric Uranyl(V) Complex **5** and Monomeric Uranyl(V) Complex **7**

The sterically bulky bis(trimethylsilyl)amide ligand, (N(SiMe_3_)_2_)^−^, (N″),^1−46^ was selected to stabilize [U^V^O_2_]^+^ complexes, since the ligands lack strongly absorbing chromophores. This approach has proven successful in enabling the identification and characterization of the optical properties of U^4+^, [Np^V^O_2_]^+^, and [Np^VI^O_2_]^2+^.^47^ Uranyl(VI) silylamide complexes [K(THF)_3_][UO_2_(N″)_3_(THF)] (**1**) and [UO_2_(N″)_2_(THF)] (**4**) were synthesized via salt metathesis between [UO_2_(Cl)_2_(THF)_2_]_2_ and ligand transfer reagent KN″ in a 1:3 (Scheme 1) or 1:2 ratio (Scheme 2). Complex **1** was converted to [K(crypt)][UO_2_(N″)_3_] (**2-crypt**, crypt = 2.2.2-cryptand) by reaction with one equivalent of 2.2.2-cryptand, while the analogous complex [K(18C6)][UO_2_(N″)_3_] (**2–18C6**, 18C6 = 18-crown-6) was obtained by reacting **4** with one equivalent of KN″ and 18-crown-6. The monomeric uranyl(V) derivatives [K(L)(DME)_n_]_2_[UO_2_(N″)_3_] (**3-crypt**, L = crypt, *n* = 0; **3–18C6**, L = 18C6, *n* = 1) were obtained in moderate crystalline yields via reduction of **2-crypt** or **2–18C6** with one equivalent of KC_8_ in the presence of corresponding sequestering agents (Scheme 1). When the reactions were carried out in the absence of sequestering agents, the only identifiable product was [K(DME)_4_][UO_2_(N″)_3_]. On the other hand, when the bis-amide precursor **4** was reduced with KC_8_ and 2.2.2-cryptand, the uranyl(V) dimer [K(crypt)]_2_[(UO(μ-O)(N″)_2_)_2_] (**5**) was obtained in low yields (Scheme 2). The isolation of **5** is rather challenging, due to its inherent instability and the concomitant formation of the peroxo byproduct [K(crypt)]_2_[(UO_2_(N″)_2_)_2_(μ-O_2_)] (**6**). When the same reduction was attempted in the presence of 18-crown-6, the uranyl(V) ate-complex [(UO_2_(N″)_2_)(μ-Cl)(K(18C6))_2_] (**7**) was obtained; its formation was likely due to the presence of KCl in the starting material. Therefore its preparation was purposely attempted by reacting **4** with an equimolar ratio of KCl and 18-crown-6, which resulted in the formation of **7** in moderate yields (Scheme 2). All compounds were thoroughly characterized via spectroscopic and analytical techniques, with the exception of **5** and **6**: the former is a highly unstable compound which readily decomposes above −30 °C, while the latter was never isolated as an analytically pure species and has very low solubility in most laboratory solvents.

In comparison with the diamagnetic precursors **1** and **2**, the ^1^H NMR spectrum of **3-crypt** displays a broadening of the spectral lines, which is in agreement with the presence of a paramagnetic metal center. Two resonances are identified for the SiMe_3_ protons (δ_H_ = −8.47 and −4.77 ppm), displaying a noticeable line broadening (δ_H_ = −8.47 ppm, ν_1/2_ = 60 Hz; δ_H_ = −4.75 ppm, ν_1/2_ = 324 Hz). Additionally, three broad resonances each integrating for 24 protons are present in the downfield region of the spectrum (δ_H_ = 6.72 ppm, ν_1/2_ = 24 Hz; δ_H_ = 8.17 ppm, ν_1/2_ = 36 Hz; δ_H_ = 8.71 ppm, ν_1/2_ = 40 Hz). It is noteworthy that **3-crypt** displays a relatively high instability in solution at room temperature. After 48 h, the decomposition is particularly enhanced and visible in the ^1^H NMR spectrum, in conjunction with a significant shift of the 2.2.2-cryptand signals and the appearance of several other decomposition peaks (see SI, Figure S11).

The ^13^C{^1^H} NMR spectrum of **3-crypt** displays characteristic resonances for the SiMe_3_ groups (δ_C_ = 6.39 and 7.09 ppm) and CH_2_ fragments belonging to the cryptand macrocycle (δ_C_ = 58.66, 72.64, and 75.90 ppm). ^1^H and ^13^C{^1^H} spectra of **3–18C6** are very similar to those of **3-crypt**, with different signals accounting for the presence of a different sequestering agent (18c6) and DME. Additionally, one signal was observed in the ^29^Si{^1^H} NMR spectra of both **3-crypt** and **3–18C6**, resonating at −103.12 and −115.49 ppm respectively.

The ^1^H NMR spectrum of **7** displays two broad signals for the SiMe_3_ protons (δ_H_ = −8.52 ppm, ν_1/2_ = 55 Hz) and CH_2_ protons of the crown (δ_H_ = −51 ppm, ν_1/2_ = 51 Hz), with additional diamagnetic impurities similar to those observed for **3-crypt**. The corresponding signals are also observed in the ^13^C{^1^H} NMR spectrum, with peaks resonating at 6.24 (SiMe_3_) and 72.15 (18-crown-6). Unlike for **3-crypt** and **3–18C6**, no clear signal was visible in the ^29^Si{^1^H} NMR. Line broadening is typically observed in the ^1^H and ^13^C NMR spectra of U(V) species. This is also detected in the ^1^H NMR spectra of uranyl(V) species with various supporting ligands^48−51^ and bis-imido analogues, while ^13^C data is rarely reported.^52−54^ Interestingly, full width half maxima of the ^1^H NMR signals of **3-crypt** (ν_1/2_ range = 24–324 Hz) are significantly narrower than those observed in bis-imido complexes [U^V^(NDipp)_2_(bipy^R2^)_2_(X)] (Dipp = 2,6-^i^Pr_2_C_6_H_2_; bipy^R2^ = 4,4′-dialkyl-2,2′-bipyridine; R= Me, ^t^Bu; X = Cl, Br, I) (ν_1/2_ range = 154–2701 Hz).^53^

Despite their instability in solution, we were able to determine the magnetic moment, μ_eff_, of **3-crypt** and **3–18C6** at room temperature via the Evans method.^55^ These were measured at 2.45 μ_B_ and 2.67 μ_B_ respectively, thus falling within the range expected for monometallic U(V)^56^ and close to the predicted magnetic moment of the U^5+^ free ion (μ_eff_ = 2.54 μ_B_).^57^ Noticeably, the magnetic moment values obtained for **3-crypt** and **3–18C6** are higher than those of other uranyl(V) species measured with similar methods, such as [K(18-crown-6][UO_2_(salan-^t^Bu_2_)(py)] and [K(18-crown-6][UO_2_(salophen-^t^Bu_2_)(py)K] (μ_eff_ = 2.14–2.25 μ_B_) reported by Mazzanti and co-workers^50^—though it is noteworthy that the values reported by these authors are significantly lower than those obtained with solid state magnetic susceptibility measurements (2.57–2.60 μ_B_).

### Structural Characterization

**Figure 1:**
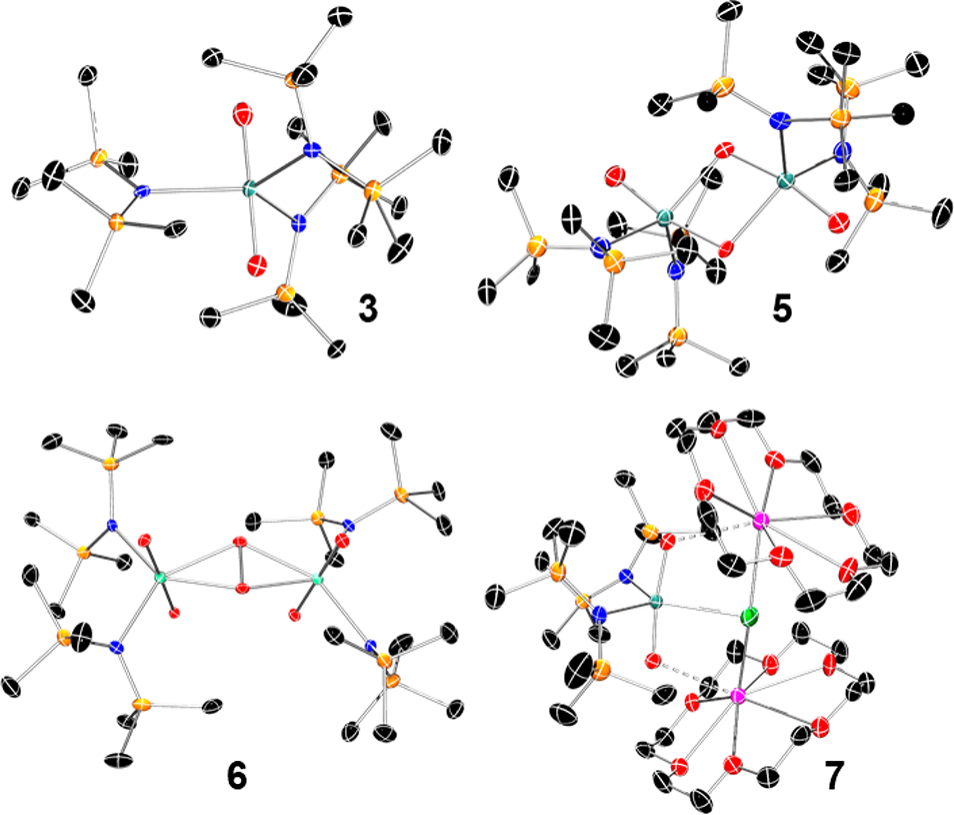
Molecular structures of **3-crypt**, **5**, **6**, and **7** determined by SC-XRD. Thermal ellipsoids are shown at 50% probability for all structures. Hydrogen atoms and outer-sphere cations are omitted for clarity. Black, blue, red, orange, green, magenta and aqua represent carbon, nitrogen, oxygen, silicon, chlorine, potassium and uranium, respectively.

The identity of these complexes was established through single-crystal X-ray diffraction (SC-XRD) experiments, which demonstrated the formulation of the anionic [UO_2_N″_3_]^2–^ fragment with two cationic [K(L)]^+^ counterions to balance the overall charge in **3-crypt** and **3–18C6** and a trigonal bipyramid geometry around the metal center, close to that observed for precursors **1**(34) and **2** (Figure 1 and S37–S39).^34^ The U=O distances of the [U^V^O_2_]^+^ fragment in **3-crypt** [1.829(3)–1.843(3) Å and **3–18C6** [1.853(3)–1.855(3) Å] are longer than those previously reported for monomeric [U^V^O_2_]^+^ species [1.736–1.821 Å] (Tables S4–S5) and may be a reflection of the strong sigma donating ability of the amide ligands.^21,25^ The [U^V^O_2_]^+^ fragment is perfectly linear in all polymorphs, together with a near perfect trigonal planar arrangement around the equatorial coordination plane. Slight elongations of the U–N distances are also observed in **3-crypt** [2.430(3)–2.476(4) Å] with respect to the [U^VI^O_2_]^2+^ precursors **1** and **2-crypt** [2.310(4)–2.333(4) Å]. This trend is in-line with changes in the ionic radii of the uranium ion.^58^ In the case of **7**, the U=O distances are statistically identical with those of **3-crypt** and **3–18C6**[1.846(5)–1.848(5) Å], while the U–N bond lengths are shorter [2.409(6)–2.412(6) Å]. Interestingly, the O=U=O deviates slightly from linearity [171.8°(2)] owing to the interaction between K cations and the uranyl(V) unit.

Single crystals of **5** reveal two [UO_2_N″_2_]^+^ fragments joined through a CCI, thereby forming an oxo-bridged dimer with diamond-shaped [UO_2_]_2_ core [U···O = 2.327(13) and 2.330(13) Å] (Figure 1). Such a conformation is rare but has been previously observed in [U^V^O_2_]^+^ species.^17^ Analogously to **3**, the U–O distances in **5** are elongated [1.868(13)–1.959(13) Å] with respect to its [U^VI^O_2_]^2+^ precursor, **4**, and both O=U=O units in the dimer are significantly bent [168.5(7) and 168.8(7)]. Additionally, the distances of the terminal U–O interactions [1.940(13) and 1.959(13) Å] are significantly shorter than those bridging between the two [U^V^O_2_]^+^. In contrast to previously reported examples, in which the CCI was favored by additional interactions of the actinyl fragment,^25,29,59^ the formation of the diamond-shaped core in **5** is unsupported.

### Electronic Structure Investigation and Analysis

**Figure 2:**
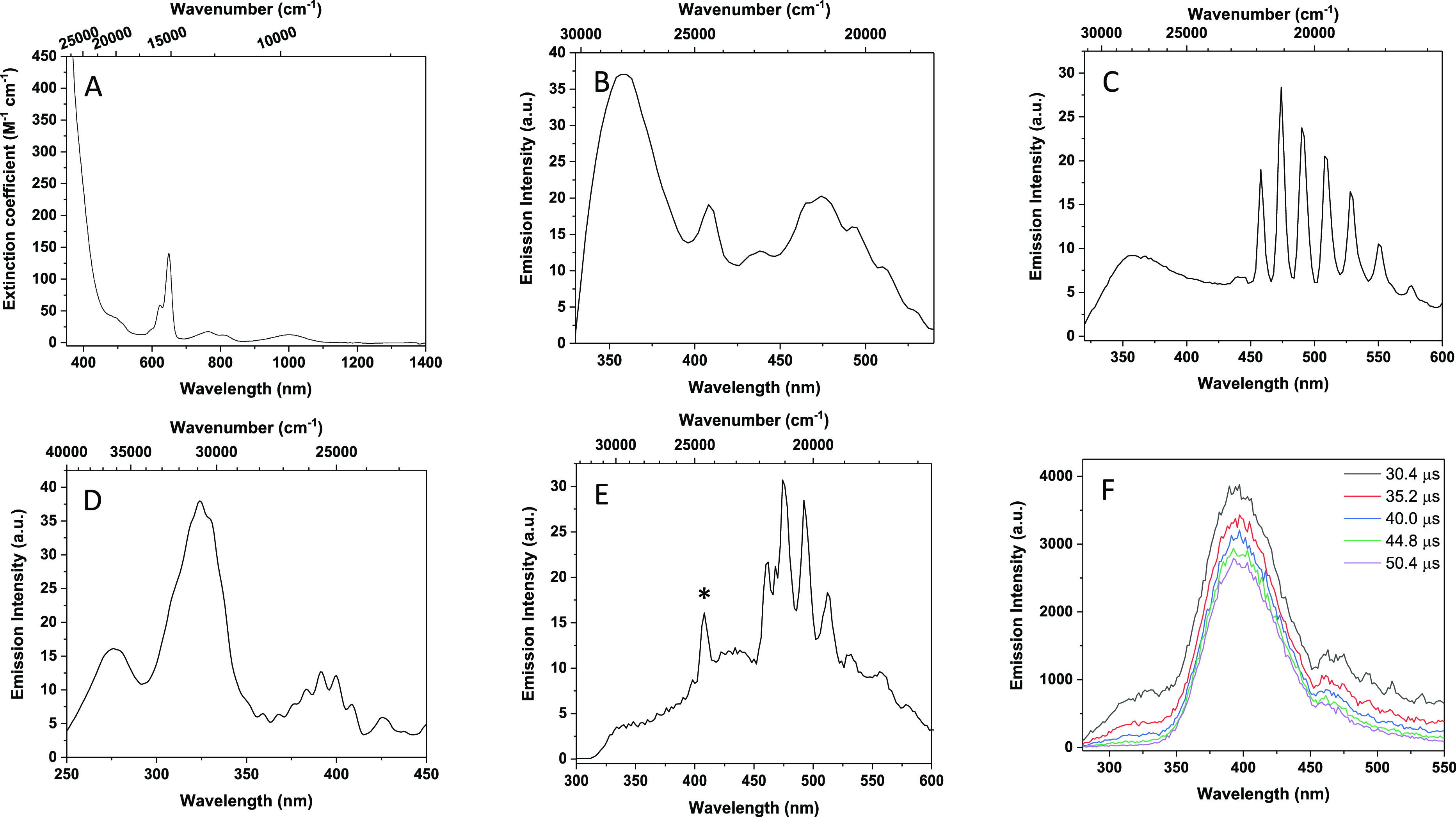
Representative spectra of **3-crypt**: (A). Electronic UV–vis–nIR absorption spectrum (2.9 mM, DME). (B) Corrected steady state emission spectrum recorded (295 K, 2-Me-THF, λ_exc_ = 280 nm). (C) Corrected steady state emission spectrum recorded at 77 K (frozen 2-Me-THF, λ_exc_ = 300 nm). (D) Corrected excitation spectrum recorded at 77 K (frozen 2-Me-THF, λ_em_ = 475 nm). (E) Corrected steady state emission spectrum of ground powder recorded (295 K, λ_exc_ = 280 nm). * denotes scattered light. (F) Spectrally sliced time-resolved emission spectra (Figure S51) of powdered **3-crypt** (295 K, 230 nm excitation, recorded over a 400 μs time domain).

**Figure 3:**
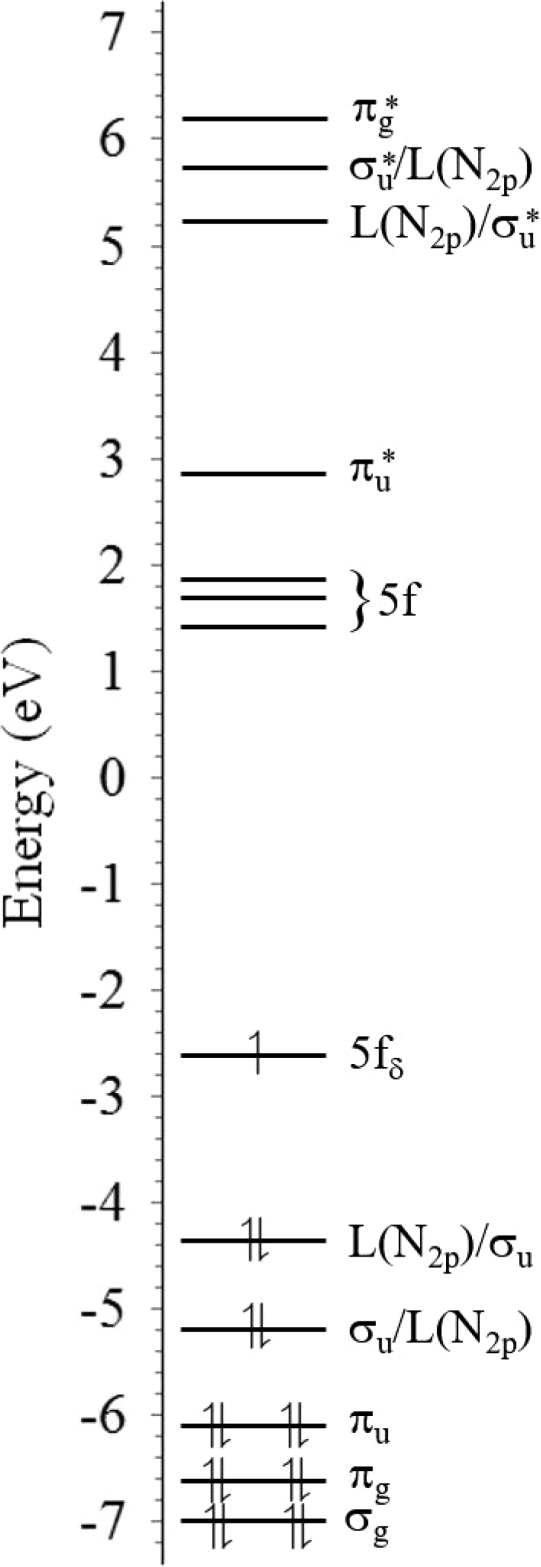
Computed MO diagram of **3-crypt** in the ground state. For clarity, ligand orbitals are not explicitly shown. The principal excitation promotes an electron from the σ_u_/L(N_2p_) to the 5fδ_u_ orbital(s) (labeled as 5f above, leaving an unpaired electron in the σ_u_, 5f δ_δ_, and 5fδ_u_ orbitals).

The UV–vis–nIR absorption spectra of **3-crypt** in DME further demonstrate the presence of pentavalent uranyl (Figure 2A). The spectrum shows a broad, intense feature centered at υ_max_ = 22 000 cm^–1^ extending to higher energies, plus a series of sharper electronic transitions at approximately 15 600, 13 300, 12 500, 10 000, and 1440 cm^–1^ (λ_max_ = 641 (ε = 145 M^–1^ cm^–1^)), 752 (ε = 20 M^–1^ cm^–1^), 800 (ε = 15 M^–1^ cm^–1^), 1000 (ε = 15 M^–1^ cm^–1^) and 6944 (ε = 145 M^–1^ cm^–1^) nm) at lower energies (Figure S41 and S42 (SI)). These transitions are assigned as admixtures of 5f ← U=O_(yl)_, 5f ← amide, intraligand, intra 5f, 6d ← 5f, and 7s ← 5f transitions by comparison with the calculated excitations in **3**-**crypt** (see Figure S80 and Table S6, SI for full assignments). In comparison with experimental findings on related systems^30^ and theoretical calculations for the bare [UO_2_]^+^ ion,^61,64−69^ the excitations can be approximately assigned as transitions involving the σ_u_φ_u_δ_u_ (15 600 cm^–1^), σ_u_^2^π*_u_ (13 300 cm^–1^), σ_u_^2^π*_u_/σ_u_^2^φ_u_/σ_u_^2^δ_u_ (12 500 cm^–1^/10 000 cm^–1^) electronic configurations (Figure 3).

Solvatochromism of **3-crypt** is further evidence the charge-transfer character of absorptions in the visible region: dissolution in pyridine converges the energies of the visible and nIR absorption bands that span the 16 000 to 9000 cm^–1^ range (625–1110 nm, see Figure S42 (SI)).

The diagnostic spectroscopic features of [U^V^O_2_]^+^ were further defined via vibrational spectroscopy, including the first reported Raman spectra for well-defined molecular pentavalent uranyl species. Solid-state Raman spectra of **1** and **2-crypt** have been previously reported with U=O_(yl)_ symmetric stretching modes at 802 and 809 cm^–1^, respectively. A progression is often observed with the archetypal uranyl(VI) σ_u_ ← δ_u_/φ_u_LMCT emission (←λ_max_ ∼ 510 nm). In the solid-state Raman spectrum of **3-crypt** several signals are observed (Figure S25), including the equivalent U=O_(yl)_ symmetric stretching mode at 753 cm^–1^ in THF solution, with a shoulder peaking between 682 and 697 cm^–1^ (this feature is resolved in **3–18C6** at 727 cm^–1^). The shift of the ν_1_(U=O_(yl)_) symmetric stretch between [U^VI^O_2_]^2+^ and [U^V^O_2_]^+^ species is consistent with a decrease in the nuclear charge at uranium. This effect can also be quantified by calculating the relative stretching and interaction force constants (*k*_1_ and *k*_12_).^34,70,71^ The uranyl(VI) precursors **2–18C6** and **2-crypt** have stretching force constants of 6.85 and 6.94 mdyn Å^–1^ respectively. As expected, the force constants decrease for **3–18C6** and **3-crypt** and are measured at 5.66 and 5.53 mdyn Å^–1^, respectively.^72^ These experimental spectra are well-reproduced by DFT calculations (geometry optimization and vibrational analysis, Figure S81).

This vibrational analysis sets the stage for the detailed understanding of the photoluminescence spectra.^62,63^ Prior to this work, observation of uranyl(V) species has thus far been limited to in situ studies of species generated from uranyl(VI).^31,32^ In both instances, emission spectroscopy revealed broad featureless emission bands centered at 405 and 440 nm respectively following 255 nm excitation. with luminescence lifetimes attributed to uranyl(V) carbonate emission of 140 μs at 153 K and at pH 2.4, for the aqua ion 1.1 μs at 298 K (compared to 0.9 μs for the equivalent uranyl(VI) species also present in the solution at room temperature).

Very recently, Mazzanti and co-workers reported the emission properties of a water-soluble uranyl(V) dipicolinic acid (dpa) complex [K(2.2.2.crypt)]_2*n*_{[KUO_2_(dpa)_2_]}.^18^ in this system, the emission spectrum exhibits a broad profile with two maxima at 404 and 459 nm following excitation at 360 nm, with corresponding excitation peaks for the 404 nm emission band at 335 and 360 nm. Upon excitation at 459 nm, the second maxima is shifted to lower energy. At low temperatures (77 K), it was observed that the emission profile becomes well resolved with seven distinguishable vibrationally resolved lines in addition to lower intensity vibrationally resolved emission up to 550 nm; the excitation spectra also exhibit four sharp lines centered at ca. 360 nm. The decay kinetics showed bi- or multiexponential behavior, with the longest component resolved as 11 μs. These data are broadly in agreement with those observed for **3-crypt** reported herein.

The photophysical properties of the uranyl(VI) tris amide precursor, **2-crypt**, [K(2.2.2-cryptand)][UO_2_{N(SiMe_3_)_2_}_3_]are shown in Figures S56–S60. The principal peak positions in the emission spectrum are typical of uranyl(VI) and are 528 nm (18 939 cm^–1^), 550 nm (18 797 cm^–1^), 554 nm (18 182 cm^–1^), 576 nm (17 361 cm^–1^), with vibrational spacings measured as 757 and 821 cm^–1^. Here, the emission maximum is significantly red-shifted (550 nm) compared to aqueous uranyl salts (ca. 510–520 nm) due to the strong amide sigma donors located in the equatorial plane.^33,47^

Continuous-wave UV excitation (230–300 nm) of mM, room temperature DME, THF and 2-Me-THF solutions of **3-crypt** (Figure 2B) results in broad emission spectra, which are comprised of two principal bands centered at 355 and 475 nm (26 170 and 21 050 cm^–1^). In room temperature 2-Me-THF, the 475 nm-feature displays discernible vibrational progression. Upon freezing to a glass at 77 K in 2-Me-THF, this vibrational fine structure becomes pronounced revealing seven vibronic transitions with peak-to-peak separations of 737, 772, 717, 669, 757, and 760 cm^–1^ (average = 735 cm^–1^, Figure 2C). The apparent zero-phonon (*E*_0–0_) transition estimated between the first and second highest energy vibronic transitions at 737 cm^–1^ matches well with the U=O_(yl)_ symmetric stretch measured experimentally by Raman spectroscopy (753 cm^–1^) and predicted computationally (711 cm^–1^) for the modes that possesses the most symmetric U=O_(yl)_ stretching character. However, this vibration also possesses a degree of U–N{(SiMe)_3_}_2_ character, and computational analysis suggests that all of the other vibrations with symmetric stretching character are coupled to other molecular motions (723, 725, 728, and 732 cm^–1^). The amide N–(SiMe)_3_ vibrations contribute to the vibrations at frequencies of 724, 728, and 736 cm^–1^, where the latter is the most pronounced.^60,61^ Therefore, more than one vibrational progression may be contributing to the spectrum.

The excitation spectra of **3-crypt** (frozen 2-Me-THF) monitored at the lowest energy emission maximum (475 nm) reveal three separate excitation regions centered at 275, 324, and 390 nm (Figure 2D), whereas an excitation band at 220 nm is responsible for the emission at 355 nm. In the excitation spectrum, the lower energy absorptions appear to exhibit vibrational fine structure. This feature is particularly pronounced in the lowest energy excitation band (390 nm), with six measurable maxima (frequency difference between the two lowest energy peaks is 490 cm^–1^, average peak-to-peak separation, 533 cm^–1^). These features are reminiscent of the vibrationally resolved LMCT absorptions exhibited in many [U^VI^O_2_]^2+^ compounds, but here may, in principle, arise from the multiple different excitations predicted in this region (Figure S80 and Table S6).

The luminescence lifetimes recorded at the emission maxima (475 nm) of **3-crypt** in a frozen glass at 77 K (Figure 2C and Figure S44, Table 1) are biexponential with the short component resolved as 1.02 μs (25%), and the longer one at 8.22 μs (75%). This observation implies that there are several excited states contributing to the observed emission (as supported by theory). At room temperature (295 K), the corresponding lifetimes of the 475 nm band are much shorter (2.1 and 9.5 ns) and could only be measured accurately following pulsed picosecond excitation (375 nm).

**Table 1:** Summary of Lifetime Data for **2-Crypt**, **3-Crypt**, **5**, and **7**^a^

complex	λ_ex_ (nm)	λ_em_ (nm)	τ_1_ (μs)/(%)	τ_2_ (μs)/(%)	τ_3_ (μs)/(%)	χ^2^
**2-crypt**^b^	250	550	5.9	40.8	176.7	1.6
			(34)	(37)	(27)	
**3-crypt**^b^	280	320	3.7	24.0	186.4	1.3
			(56)	(44)	(29)	
	280	360	4.3	77.5	153.3	1.2
			(12)	(51)	(36)	
	280	492	5.2	41.2	204.1	1.0
			(11)	(31)	(58)	
	280	580	6.9	44.2	198.7	1.3
			(11)	(36)	(53)	
**3-crypt**^c^	300	420	1.7	7.1	–	1.2
			(47)	(53)		
	300	475	1.02	8.22	–	1.3
			(25)	(75)		
**5**^b^	230	370	109.5	–	–	1.3
			(100)			
	230	440	130.1	–	–	1.2
			(100)			
	230	550	5.2	57.4	160.5	1.0
			(1)	(9)	(90)	
**5**^c^	260	430	1.0	8.2	–	2.1
			(22)	(78)		
	260	535	55.1	148.0	–	1.1
			(28)	(72)		
	260	590	51.9	139.2	–	1.3
			(27)	(73)		
**7**^c^	325	480	11.0	102.9	–	1.1
			(10)	(90)		
	325	550	45.6	202.5	–	1.2
			(78)	(32)		

a Estimated error ± 10%.

b Sample measured at 295 K in the solid state.

c Sample measured at 77 K in a frozen 2-Me-thf glass.

To forestall contribution of any dynamic exchange and speciation effects, the optical properties of solid-state **3-crypt** were examined in detail. The steady-state spectrum of **3-crypt** as a powdered sample (295 K), (Figure 2E) shows a similar emission profile to those recorded in fluid solution and in a frozen glass, with overlapping broad features (λ_em_ = 320, 420, and 475 nm). Again, the emission band at 475 nm appears to exhibit vibrational progression, where the estimated *E*_0–0_ is 772 cm^–1^ (average = 743 cm^–1^). The kinetic profiles were also investigated by time-resolved emission spectroscopy (TRES, Figure 2F, S51). Notably, as a function of delay time, the emission profile of the 475 nm band resolves into two components with peak maxima at 400 and 475 nm. The longest-lived of these emission profiles is centered at 475 nm and exhibits biexponential decay behavior (τ_1_ = 4.31 μs (12%) and τ_2_ = 127 μs (88%)), whereas the emissive feature at 400 nm is vibrationally broadened and is also modeled with a biexponential decay function (τ_1_ = 5.56 μs (2%), τ_2_ = 117 μs (82%)).

By analogy with reported experimental and theoretical data of uranyl(V)^19,20,64,73^ and neptunyl(VI)^47^ 5f^1^ species (where the emission with An = O_(yl)_ to actinide charge transfer character has been observed at 405, 440, and 438 nm respectively in experiments), the equivalent lowest energy U ← U=O_(yl)_ LMCT transition is expected to lie in the UV and be blue-shifted with respect to that in uranyl(VI). In a complex of *pseudo D*_3*h*_ symmetry, as in **2** (L) and **3** (L), this transition is predicted to be formally Laporte-forbidden but may be relaxed in part with respect to the free ion of higher symmetry (*D*_∞*h*_) due to the removal of the inversion symmetry by the inclusion of the equatorial ligands. For comparison, in the neptunyl(VI) complex [NpO_2_(TPIP)_2_(Ph_3_PO)] (TPIP = tetraphenylimidodiphoshinate),^47^ the visible LMCT emission centered at 438 nm also exhibits vibrational fine structure that corresponds to a N–P vibration. The electronic excitations responsible for this emission were shown by analogous calculations to be combination of Np ← TPIP and Np ← O_(yl)_LMCT charge transfer.

**Figure 4:**
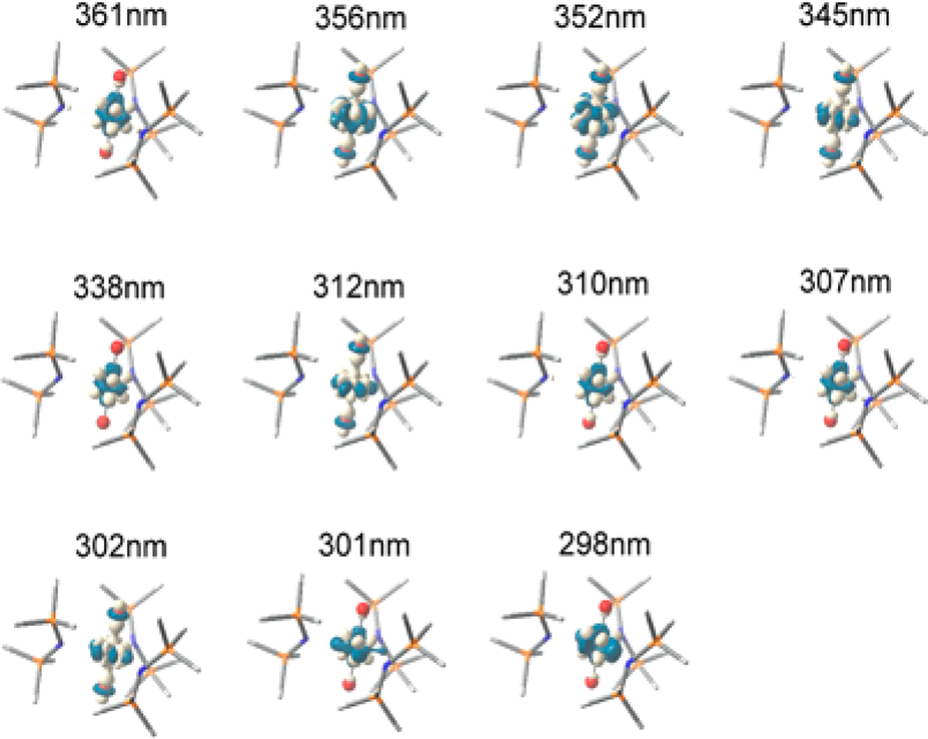
Plot of density differences between the ground and excited state for the **3-crypt**. There are transitions with O_(yl)_-f character at 356, 352, 345, 312, and 302 nm. The light regions indicate charge depletion and the blue areas charge accumulation.

To investigate the origin of the emission in **3-crypt** further, TD-DFT calculations were performed using linear response theory in the absence of point group symmetry, and a total of 100 excited states were evaluated. In preliminary studies to benchmark methods on free uranyl including hybrid-GGA (e.g., PBE0, B3LYP) and Coulomb-attenuated hybrids (e.g., CAM-B3LYP) with wave functions based methods (CASPT2) indicate little variation with respect to functional,^74−79^ and hence PBE0 was selected in an effort to minimize dependence of the simulation data on parameters not optimized for the system of interest. From a computational perspective, f-f transitions were assigned on the basis of visual inspection of electron density difference plots between ground and excited states (Figure 4).

Indeed, for the bare uranyl(VI) ion in the gas phase, our DFT simulations (PBE0/def2-TZVP) show the triplet state with single occupation of the 5fδ_u_ orbital (a fair approximation to the optically accessible excited state),^80^ whereas for the uranyl(V) cation, the equivalent excitation produces a quartet with occupation of each of the degenerate 5fδ_u_ orbitals. In both cases, the σ_u_ orbital has been deoccupied (Figure 4). The energy difference between these LMCT excitations are calculated as 2.29 eV (18 484 cm^–1^, 541 nm) for uranyl(VI) and 2.82 eV (22 779 cm^–1^, 439 nm) for uranyl(V) consistent with previous observations.^20,31,32,64^

Time-dependent DFT simulations of the excited states of **3-crypt** show numerous excitations between 200–300 nm with oscillator strengths >10^–3^ (Figure 4, Table S6). Of these, a number of excitations with 5f ← O_yl_ character (356, 352, 345, 312, 302 nm) were identified (Figure S80). Energetically, these are not well separated from the intra f-f transitions and oscillator strengths of all are typically ∼10^–4^. Higher energy excitations <45 450 cm^–1^ (220 nm) are characterized as being localized on the amide ligands, while those between 220 and 265 nm are associated with charge-transfer from the amide ligands to the uranium nonbonding 5fδ_u_ orbitals.

Attempts to investigate the luminescence properties of **3–18C6** by excitation into the UV and visible absorption bands (220–650 nm), in both solution (2-Me-THF, DME) and in the solid state were unsuccessful, with no discernible emission or excitation seen even at 77 K. This is in stark contrast to **3-crypt**. The lack of detectable emission under the same conditions in **3–18C6** may suggest that this complex is not as thermally and/or photochemically stable as **3-crypt** and rapidly reacts or decomposes to form a nonemissive (or dark) thermal or photoproduct. Indeed, this difference in stability was observed by ^1^H NMR spectroscopy as noted above. Very similar observations were seen in the luminescence experiments of the uranyl(VI) peroxo complex **6** (Figure S76).

The emission properties of the uranyl(V) complexes **5** and **7**, were also investigated, where we anticipated that any U=O_(yl)_LMCT emission bands in particular would be red-shifted slightly compared to **3-crypt** due to elongation/disruption of the linear uranyl unit.^13^ For dimeric **5**, the spectra are broader than in **3-crypt** in frozen solution and the solid state following UV excitation (280–360 nm), with peak maxima at ca. 370, 410, and 480 nm at 295 and 77 K (Figures S61–S75). The instability of **5** in optically dilute solutions and in the solid state over prolonged acquisition times precluded accurate and full data collection in addition to any data collection in fluid solution at room temperature. However, representative spectra in frozen solution at 77 K and in the solid state (Figures S61–S75) exhibit features attributable to uranyl(V) centered around 470 nm, albeit much broader as expected. There is also evidence for relatively stronger emission at higher energies (ca. 400 nm) in line with that observed for **3-crypt**. Nevertheless, the steady state emission spectra of **5** points toward the fact that higher nuclearity complexes of uranyl(V) may be observable by optical spectroscopy and show a unique spectral and temporal profile, especially when recorded at lower temperatures.

Finally for complex, **7**, the emission spectrum (Figures S77–S79) appears to be a combination of uranyl(V) and uranyl(VI), where typical uranyl(VI) emission bands (ca. 530 nm) displaying vibrational fine structure (*E*_0–0_ = 769 cm^–1^) alongside a broader feature centered at ca. 480 nm. However, the origin of this emission is not clear by examining the excitation spectra, and may be a result of uranyl(VI) LMCT from the equatorial amide/chloride donors or a mixture of uranyl(VI) LMCT and uranyl(V) LMCT emission.

Of note, we observed no emission in the near-infrared region of the electromagnetic spectrum with our current instrumentation by exciting across the UV–visible spectrum (300–850 nm). This result is in contrast to the near-infrared emission observed from NpO2^2+^ in D_2_O and in the polyoxometalate complex [Na_2_(Np^VI^O_2_)_2_(GeW_9_O_34_)_2_]^14-^ reported by Faulkner and co-workers.^72^ Here, emission between 1452 and 1580 nm was observed following ns pulsed laser excitation at 337 nm (N_2_ laser). This difference may be rationalized at least in part, by examining the respective absorption spectra, where there is a larger energy gap between the UV–visible absorptions (neptunyl(VI) LMCT and the polyoxometalate LMCT which acts to sensitize the emission in the complex) and the near-infrared absorption at ca. 1230 nm which is the characteristic absorption and assigned as an interconfigurational 5f-f transition of neptunyl(VI); i.e., they are more energetically well separated. In contrast, in **3-crypt** and the other uranyl(V) complexes reported herein, there are several broad absorptions that lie in the visible region which may preclude effective population of any near-infrared excited states. Future work is directed at examining the luminescence properties of uranyl(V) complexes using higher powered laser excitation and at temperatures below that of liquid N_2_.

## Conclusion

In conclusion, this study reports the first photoluminescence and Raman spectra of well-defined monomeric [U^V^O_2_]^+^ complexes. Experimental and theoretical studies support the assignment of the excited states in [U^V^O_2_]^+^ as principally a quartet, whose origin is an admixture of amide to U(5f) and O_(yl)_ to U(5f) with a unique vibrational progression (with smaller contributions from intra 5f excitations) where the emission band at 475 nm is best described computationally as an open-shell doublet or quartet with a high degree of mixing (compared to the well-defined triplet 5fδ_u_ excited state in uranyl(VI)). Overall, the clear definition of these luminescence steady-state and time-resolved features of the [U^V^O_2_]^+^ moiety in monomeric and dimeric complexes^20,30−32^ should enable detailed studies of [U^V^O_2_]^+^ in biological and engineered environments.^18−20,73,81,82^

## Supporting Information Available

Detailed experimental procedures, spectroscopic analysis (NMR, UV–vis–NIR, IR, Raman), crystallographic studies, additional photoluminescence experiments, discussion, and computational details (PDF)

## Accession Codes

CCDC 1944749–1944759 contain the supplementary crystallographic data for this paper. These data can be obtained free of charge via www.ccdc.cam.ac.uk/data_request/cif, or by emailing data_request@ccdc.cam.ac.uk, or by contacting The Cambridge Crystallographic Data Centre, 12 Union Road, Cambridge CB2 1EZ, UK; fax: +44 1223 336033.
