# Supplementary material for: Photoluminescence of Pentavalent Uranyl Amide Complexes
Source: J Am Chem Soc. 2021 Aug 13;143(33):13184–94. doi: 10.1021/jacs.1c05184 (PMC8397311; doi:10.1021/jacs.1c05184)
Supplement: Supplementary file 1 — ja1c05184_si_001.pdf [file ja1c05184_si_001.pdf]

Photoluminescence of Pentavalent Uranyl Amide Complexes

Fabrizio Ortu, Simon Randall, David J. Moulding, Adam Woodward, Andrew Kerridge, Karsten Meyer, Henry S. La Pierre* and Louise S. Natrajan*

e-mail: la_pierre@chemistry.gatech.edu and Louise.Natrajan@manchester.ac.uk

Supporting Information

Table of Contents

1. NMR data 2
2. IR data..13
3. Raman data 16
4. Crystallographic data 29
5. UV-vis-nIR Absorption Spectroscopy 42
6. Luminescence Spectroscopy 44
7. Calculations 65
8. References 70

1. NMR data

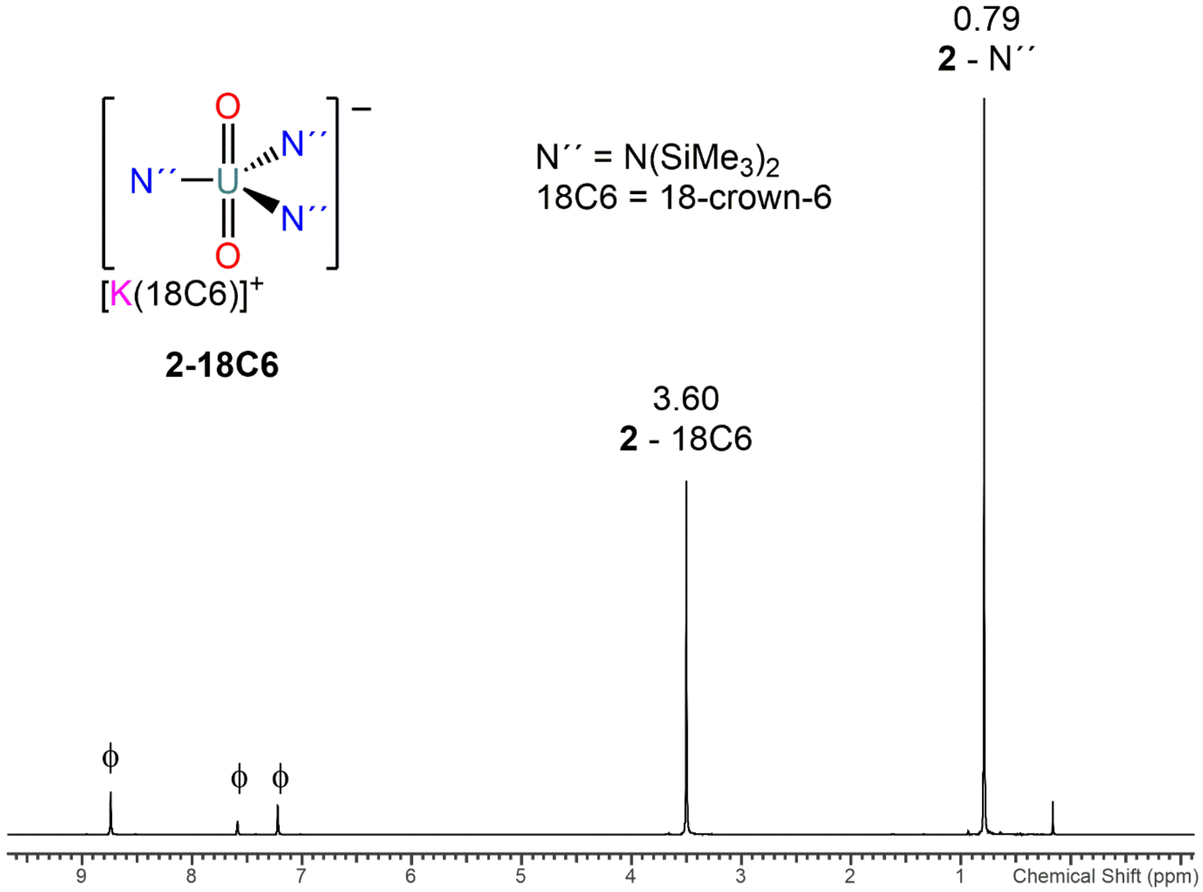

Figure S1. ^{1}H NMR spectrum of **2-18C6** (C_5D_5N, 400 MHz, 293 K). ϕ denotes C_5H_5N.

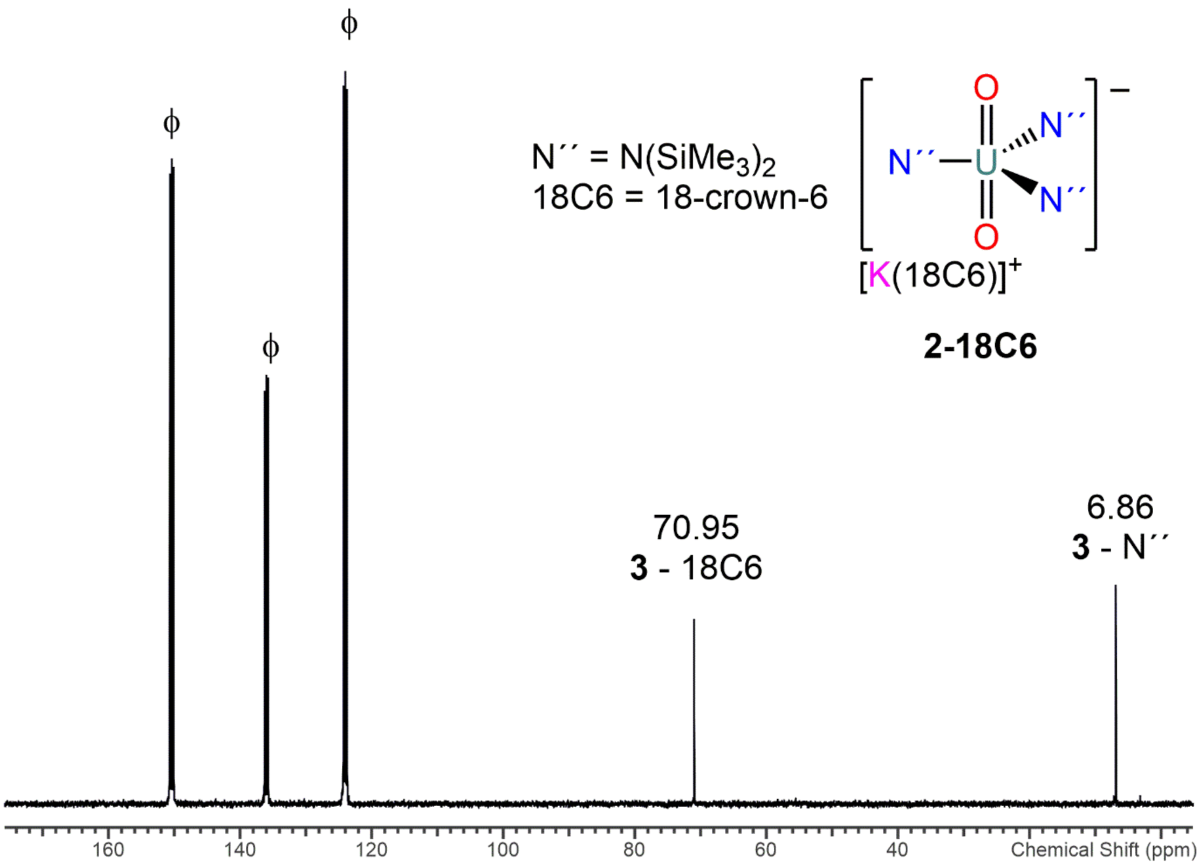

Figure S2. $^{13}C\{^{1}H\}$ NMR spectrum of **2-18C6** (C_5D_5N, 100 MHz, 293 K). ϕ denotes C_5H_5N.

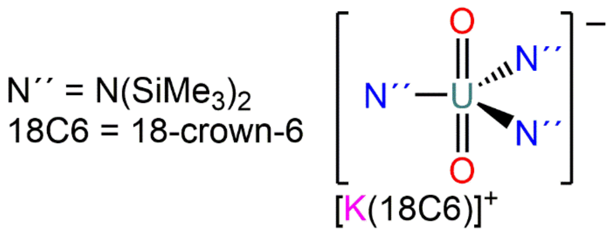

2-18C6

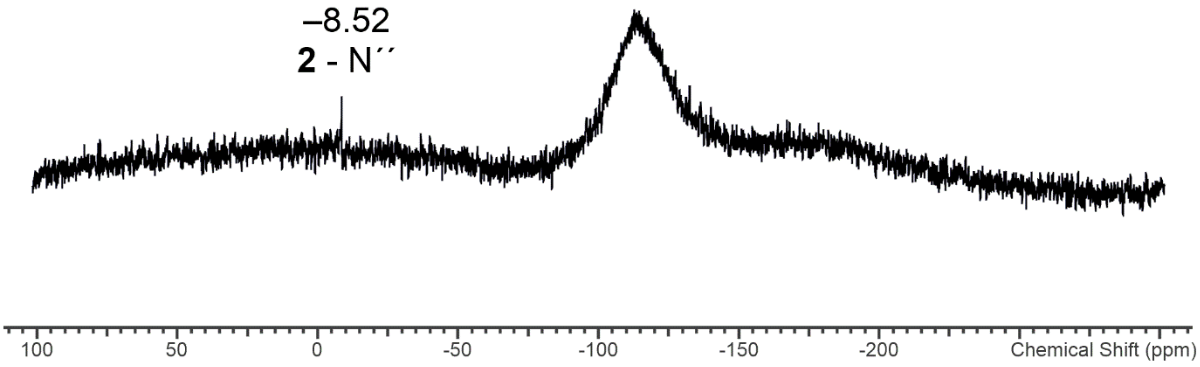

Figure S3. $^{29}Si\{^1H\}$ NMR spectrum of **2-18C6** (C_5D_5N, 400 MHz, 293 K).

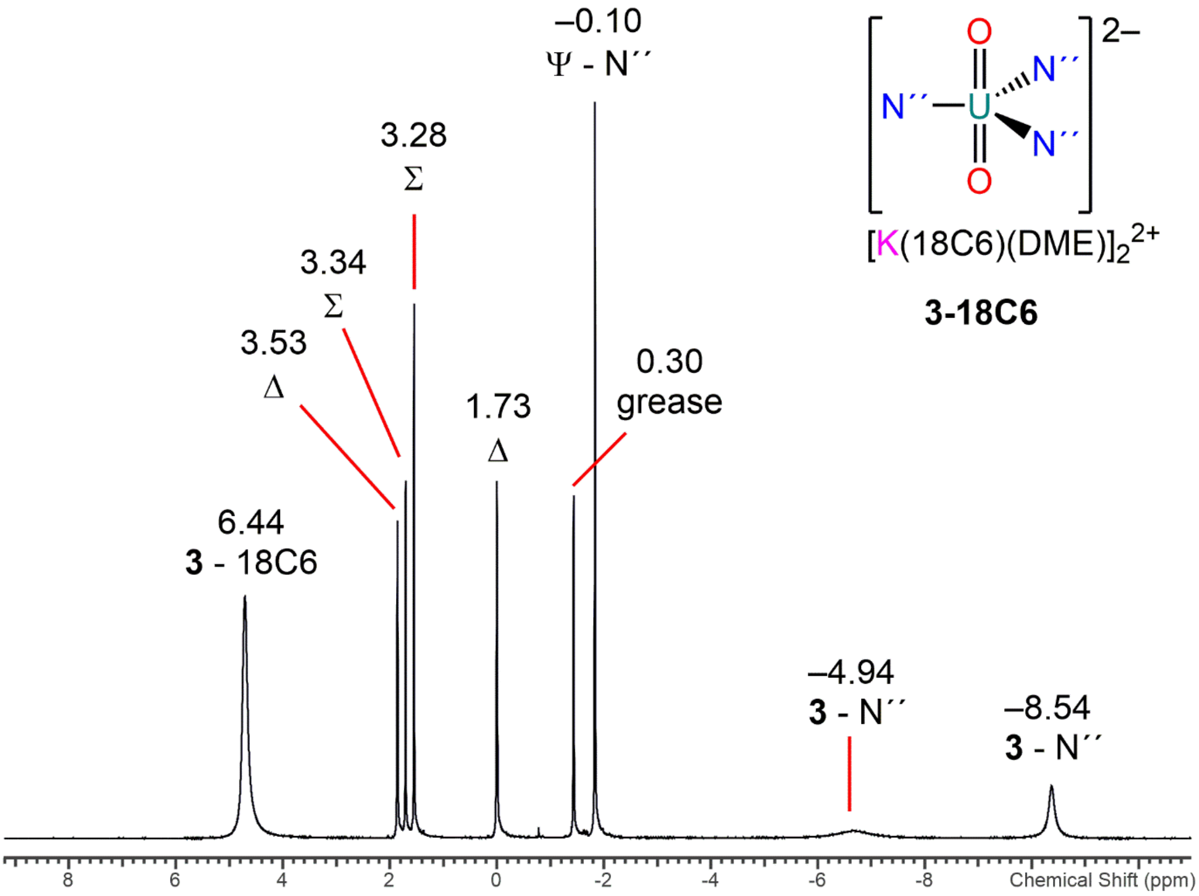

Figure S4. ^{1}H NMR spectrum of **3-18C6** (C_4H_8O, 400 MHz, 293 K). Ψ denotes **1**, Δ denotes THF and Σ denotes DME

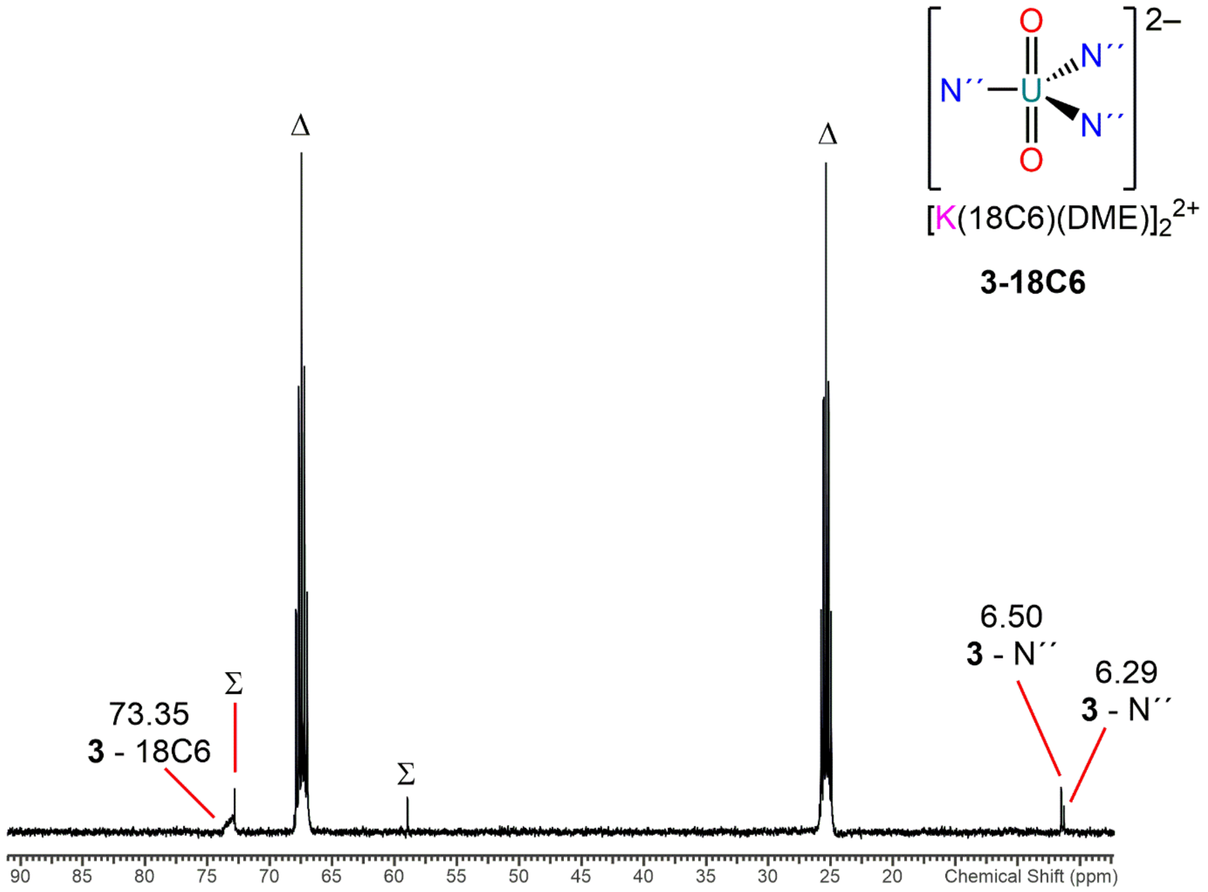

Figure S5. $^{13}C\{^{1}H\}$ NMR spectrum of **3-18C6** (C_4H_8O, 100 MHz, 293 K). Δ denotes THF and Σ denotes DME.

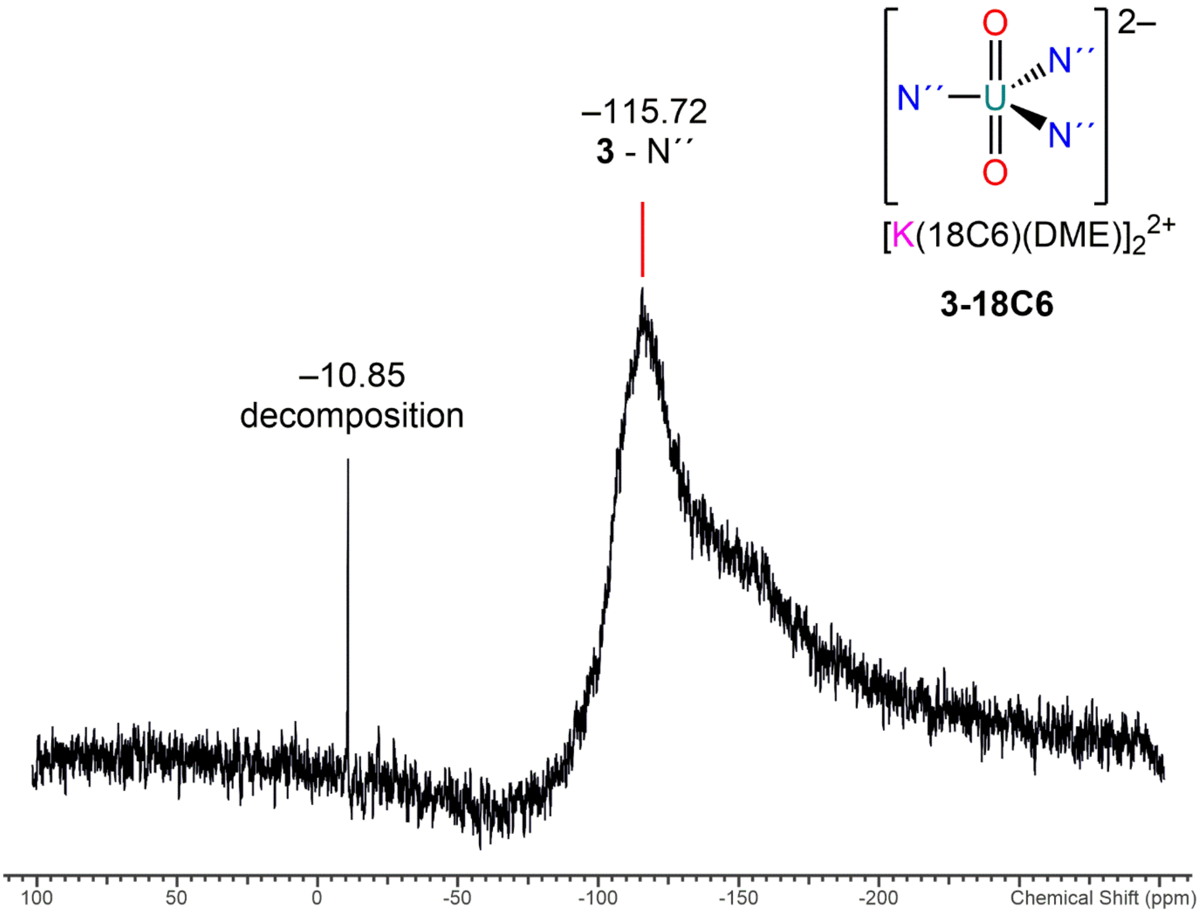

Figure S6. $^{29}Si\{^1H\}$ NMR spectrum of **3-18C6** (C_4H_8O, 79.5 MHz, 293 K).

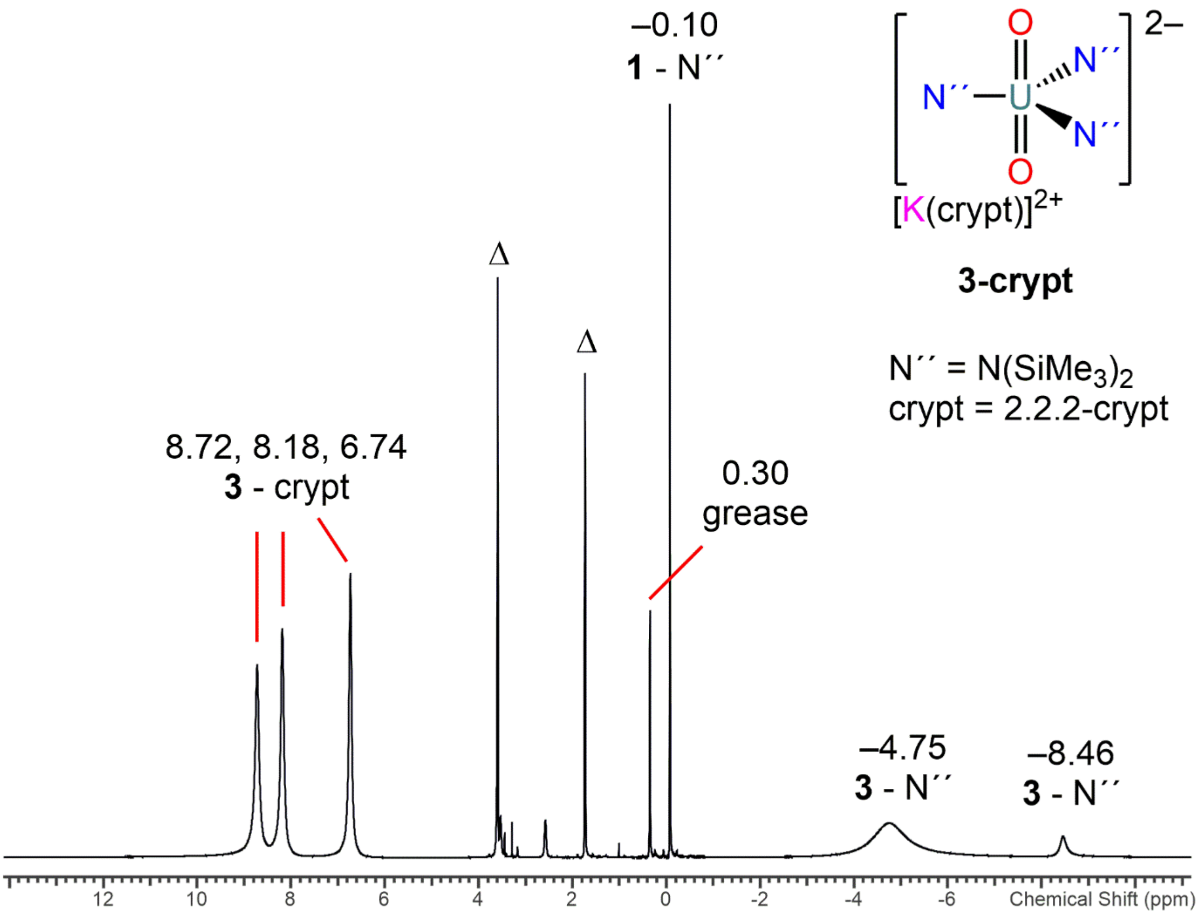

Figure S7. ^{1}H NMR spectrum of **3-crypt** (C_4H_8O, 400 MHz, 293 K). Δ denotes THF.

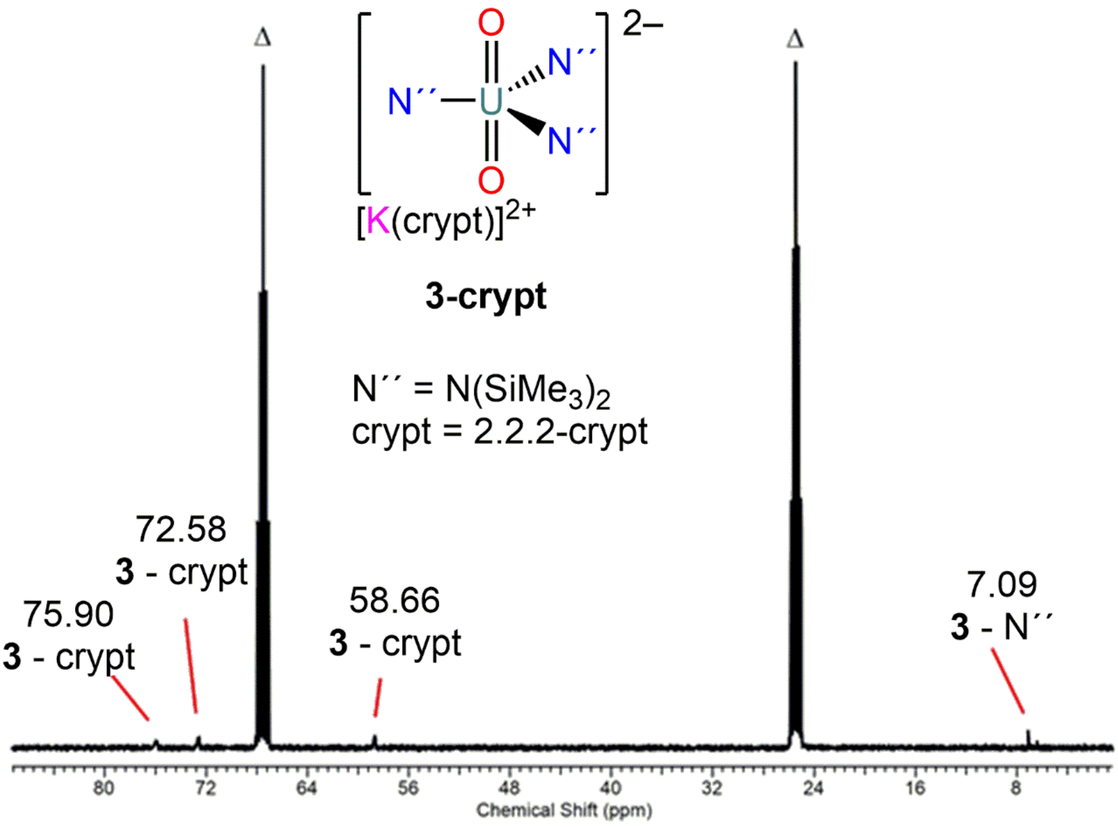

Figure S8. $^{13}C\{^1H\}$ NMR spectrum of **3-crypt** (C_4H_8O, 100 MHz, 293 K). Δ denotes THF.

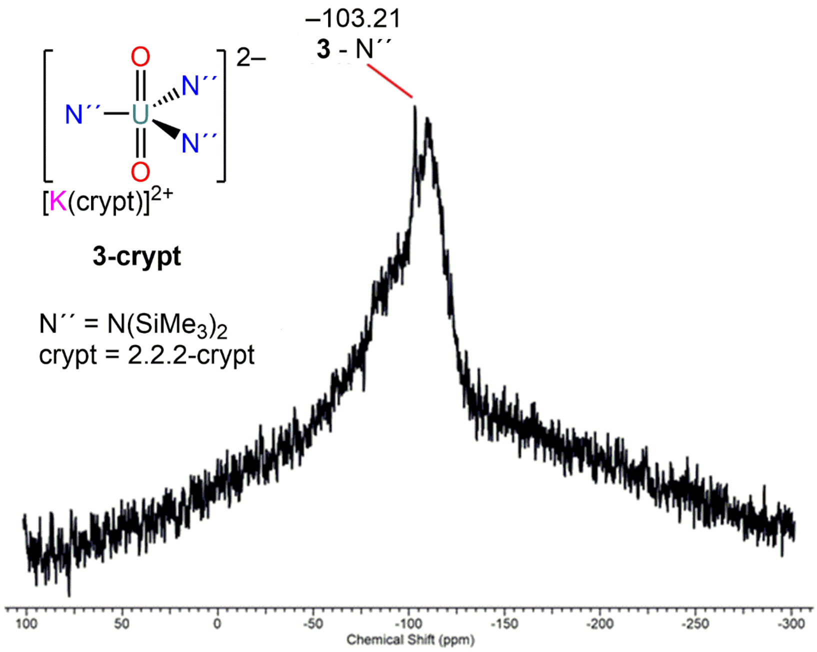

Figure S9. $^{29}Si\{^1H\}$ NMR spectrum of **3-crypt** (C_4H_8O, 79.5 MHz, 293 K).

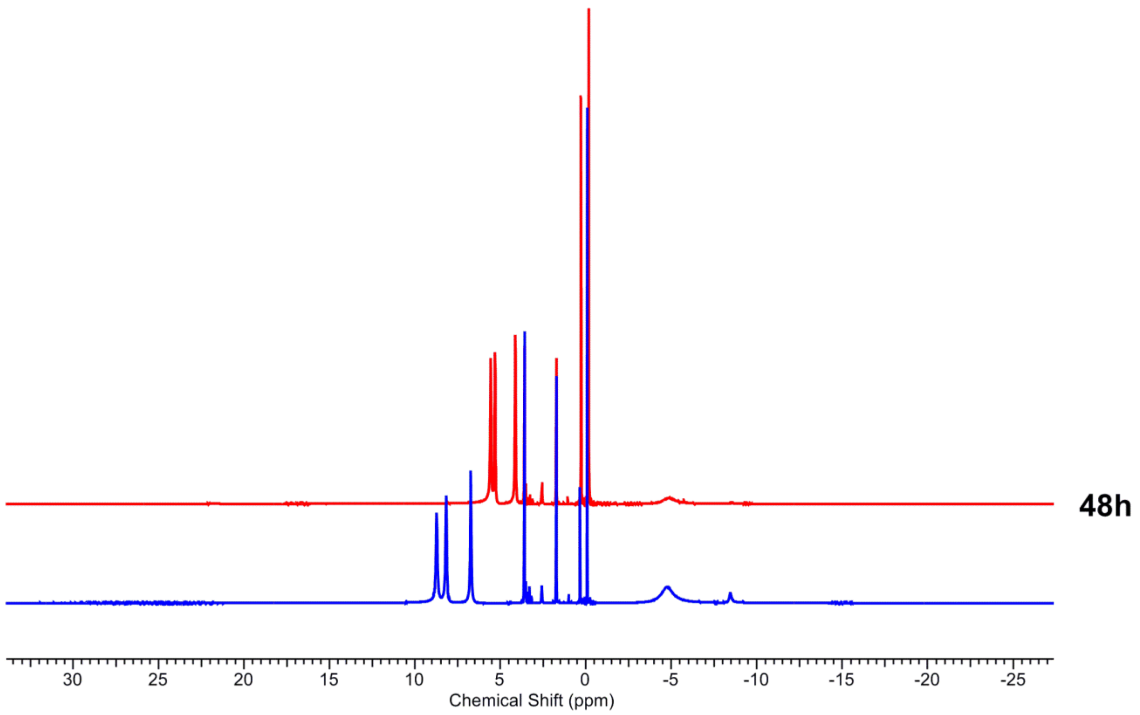

Figure S10. ^{1}H NMR spectrum of **3-crypt** (blue trace) and its decomposition (red trace) after 48h (C_4H_8O, 400 MHz, 293 K).

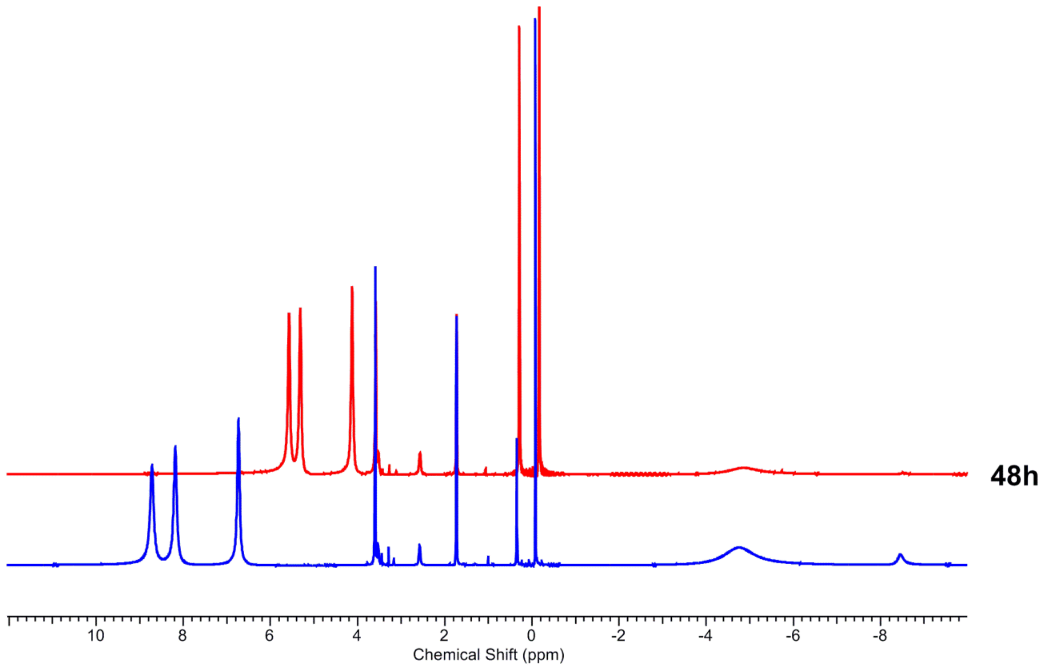

Figure S11. ^{1}H NMR spectrum of **3-crypt** (blue trace) and its decomposition (red trace) after 48h, zoomed in the region between 11 and –9 ppm (C_4H_8O, 400 MHz, 293 K).

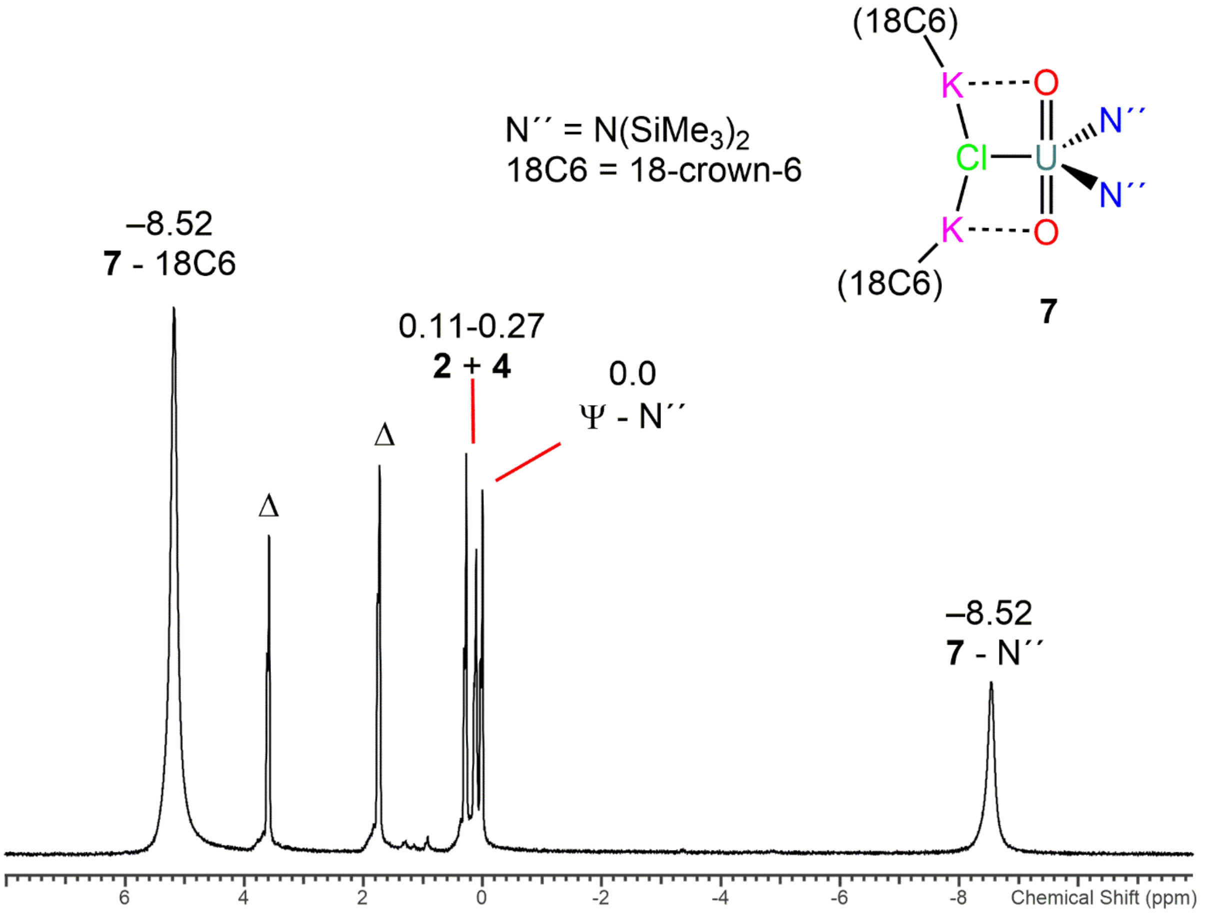

Figure S12. ^{1}H NMR spectrum of **7** (C_4H_8O, 400 MHz, 293 K). Ψ denotes $K[N(SiMe_3)_2]$, Δ denotes THF.

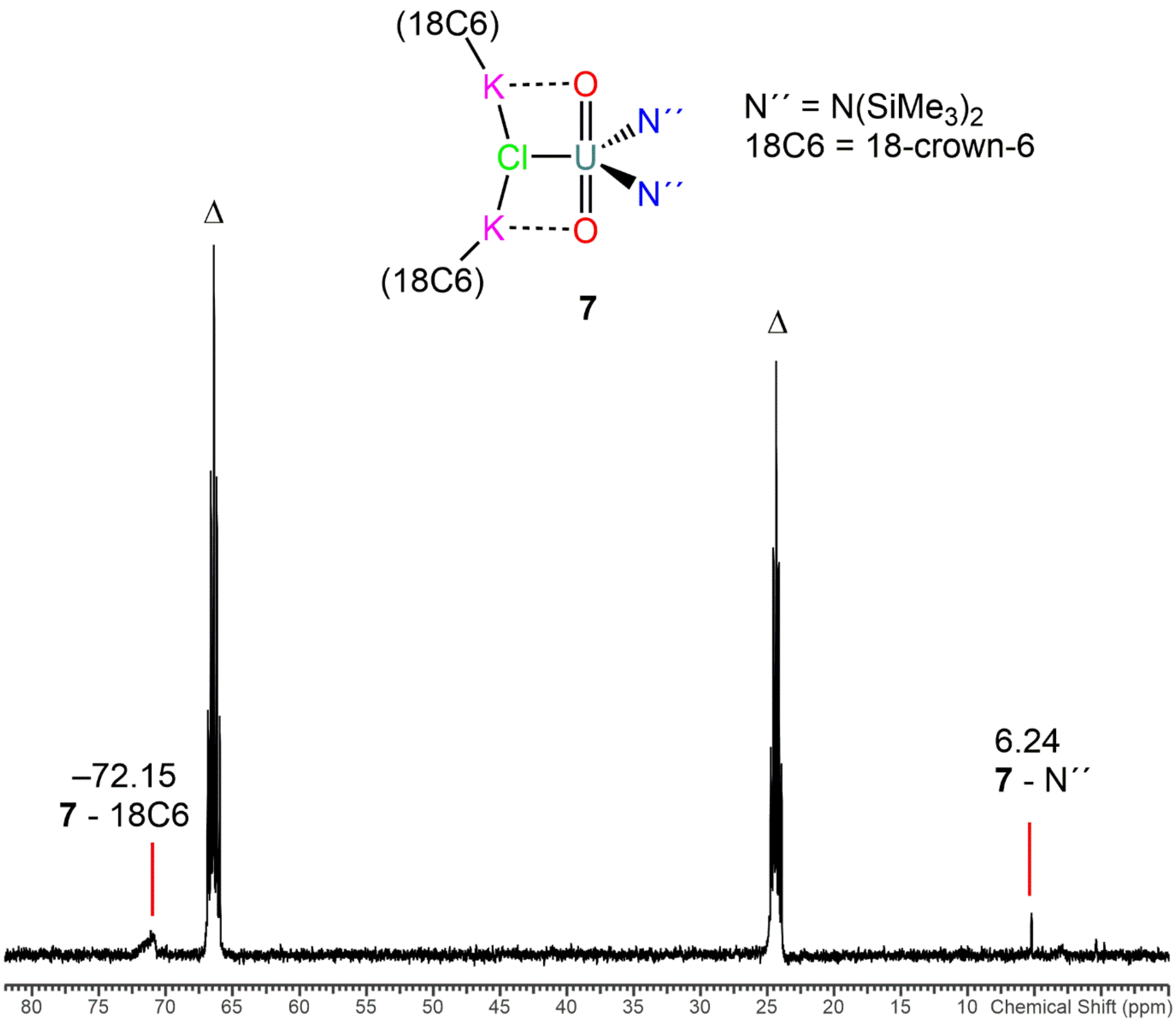

Figure S13. $^{13}C\{^{1}H\}$ NMR spectrum of **7** (C_4H_8O, 100 MHz, 293 K). Δ denotes THF.

2. IR data

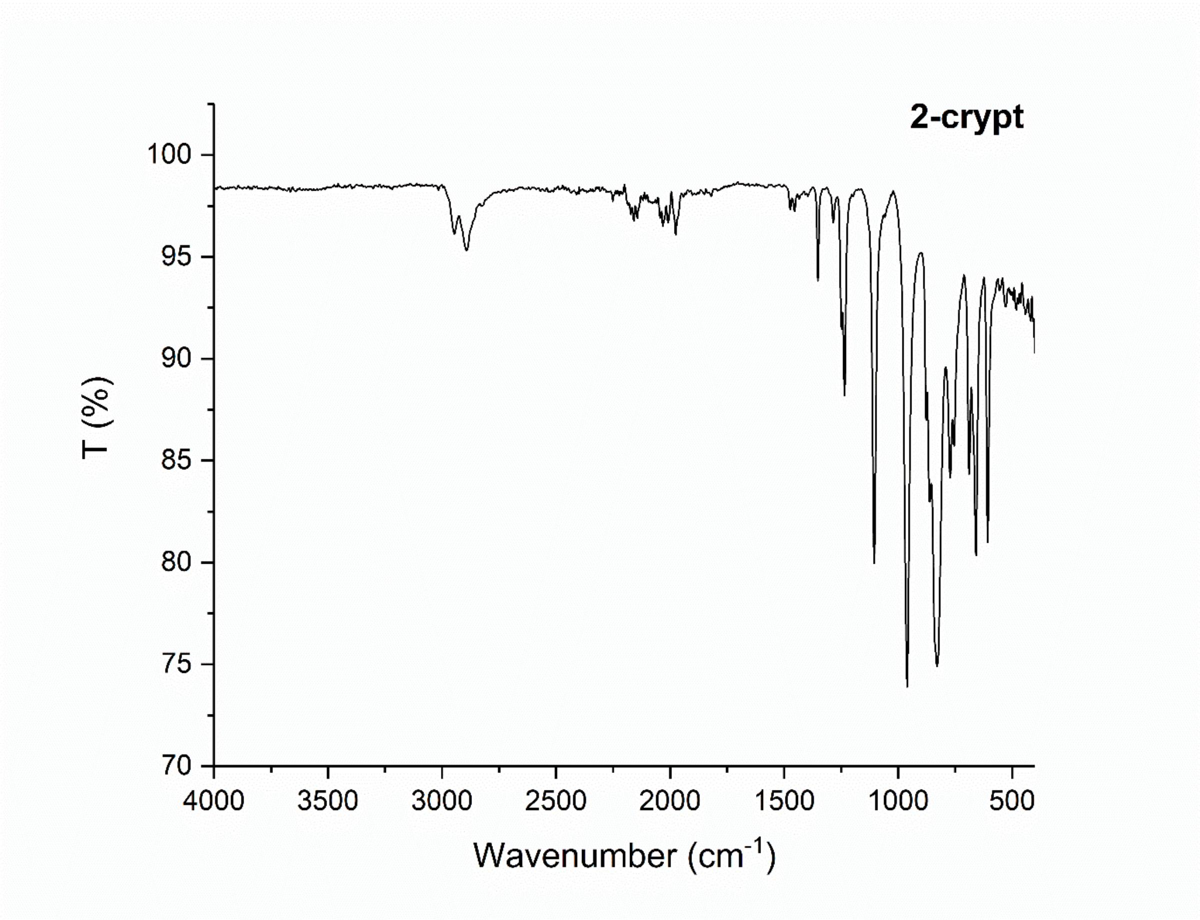

Figure S14. ATR-IR spectrum of **2-18C6**.

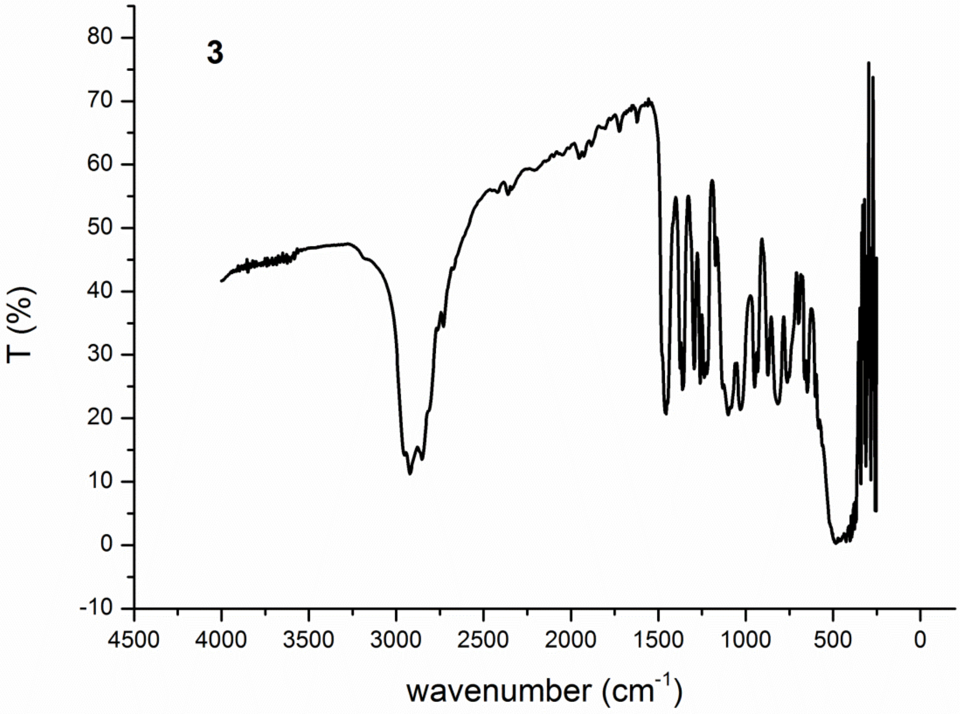

Figure S15. FTIR spectrum of **3-crypt** (KBr disc in Nujol mull).

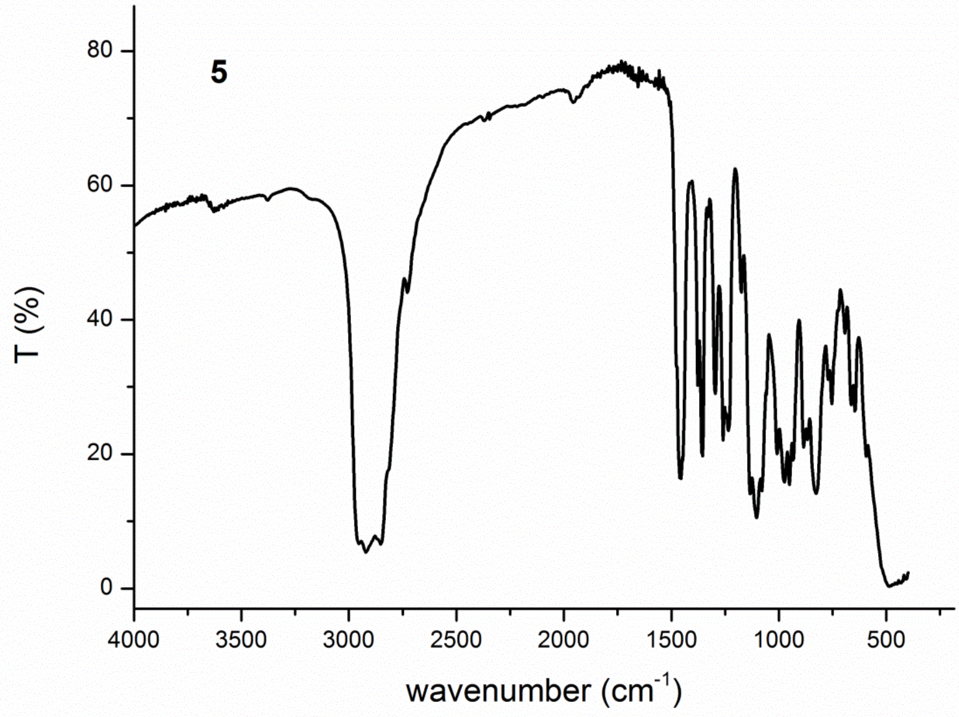

Figure S16. FTIR spectrum of **5** (KBr disc in Nujol mull).

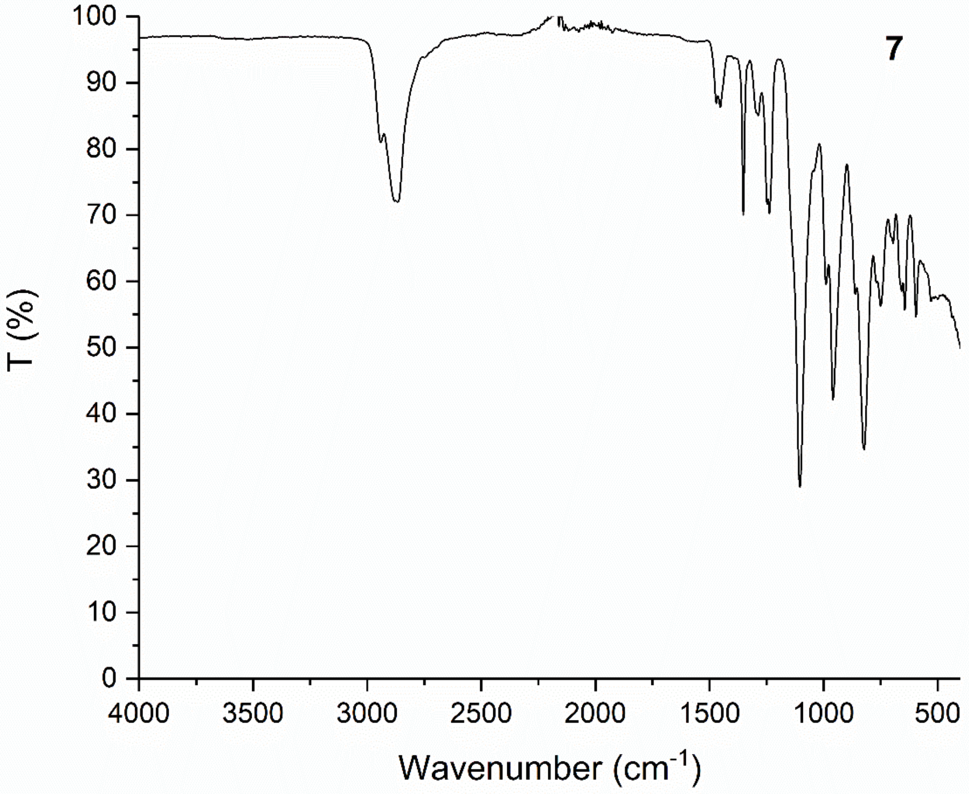

Figure S17. ATR-IR spectrum of **7**.

3. Raman data

Raman data for **1** was previously reported.[1] We recorded a new spectrum in THF solution revealing an apparent doublet at 819-828 cm^{-1} (Figure S18 and S19); a similar feature is also present in the THF solution spectrum recorded for **2-crypt**, displaying a peak at 815 cm^{-1} (Figure S22 and S23). On the other hand, in the case of the uranyl(V) congener **3-crypt**, two weak signals were detected at 753 and 829 cm^{-1} in THF solution (Figure S27 and S28); **3-crypt** has very limited stability in solvent media, therefore the stretch at 829 cm^{-1} could be assigned to the presence of decomposed uranyl(VI) material. Upon exposing a solid sample of **3-crypt** to air, all the features present in its spectrum (Figure S25) disappear and an intense signal is observed at 770 cm^{-1} (Figure S26), which is consistent with the presence of an oxidised uranyl(VI) species (Figure S20). To our knowledge, the spectra of **3-crypt** (Figure S25, S27 and S28), **3-18C6** (Figure S24) and **7** (Figure S29) are the first reported Raman spectra for a discrete and well-defined pentavalent uranyl species.

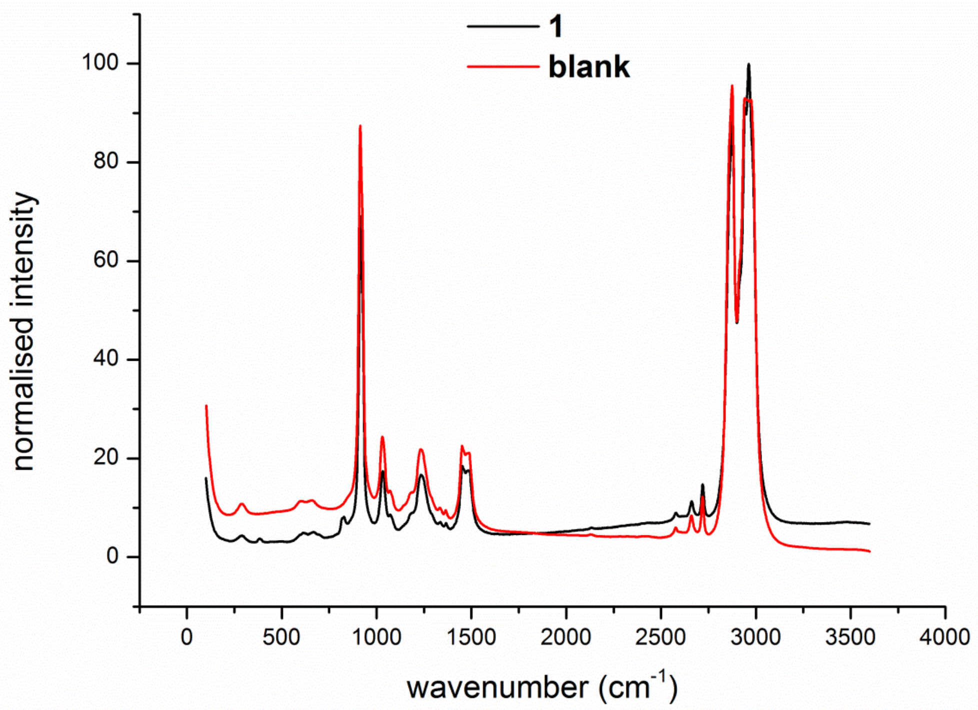

Figure S18. Raman spectrum of **1** (10mM in THF solution) with THF blank. The spectrum was acquired using a 532 nm laser, 2400 g/mm grating, 1 s exposure and 10 accumulations.

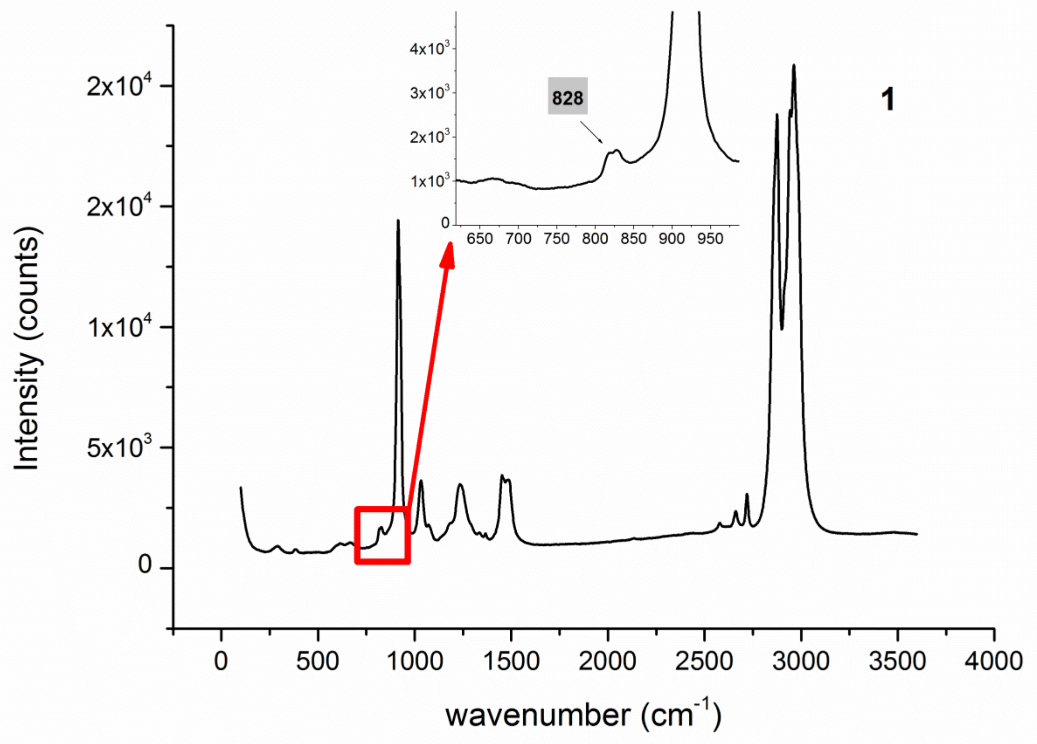

Figure S19. Raman spectrum of **1** (10mM in THF solution). The spectrum was acquired using a 532 nm laser, 2400 g/mm grating, 1 s exposure and 10 accumulations.

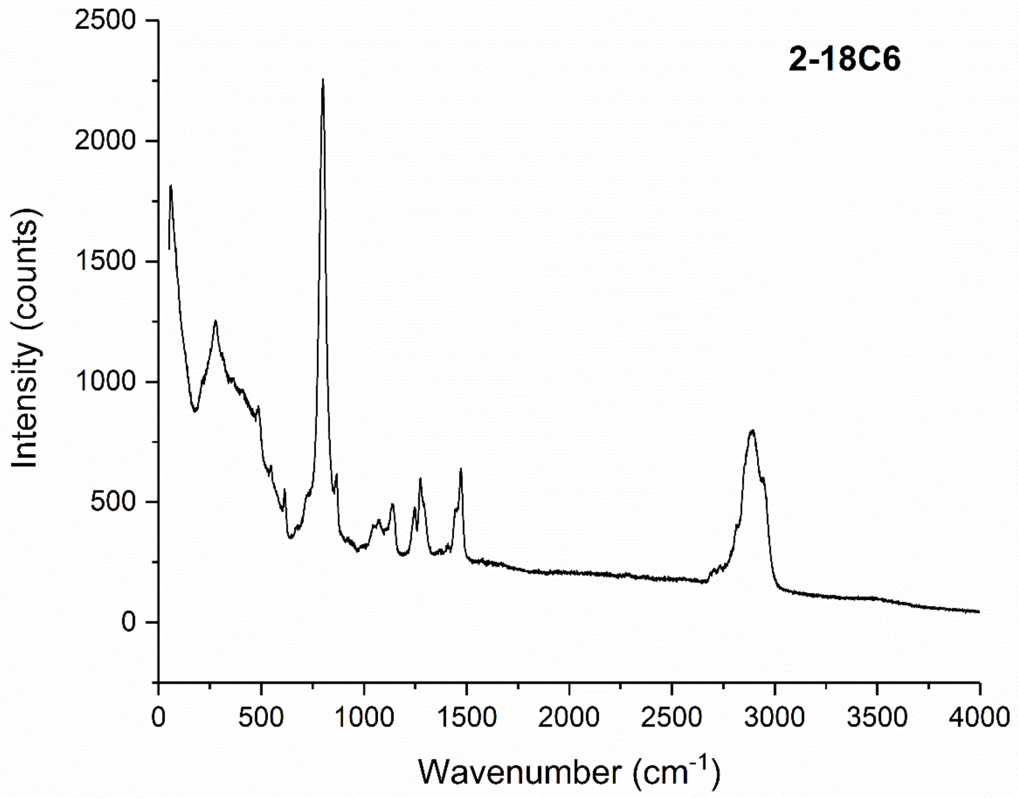

Figure S20. Raman spectrum of **2-18C6** (solid sample, quartz slide). The spectrum was acquired using a 638 nm laser, 1200 g/mm grating, 5 s exposure and 10 accumulations.

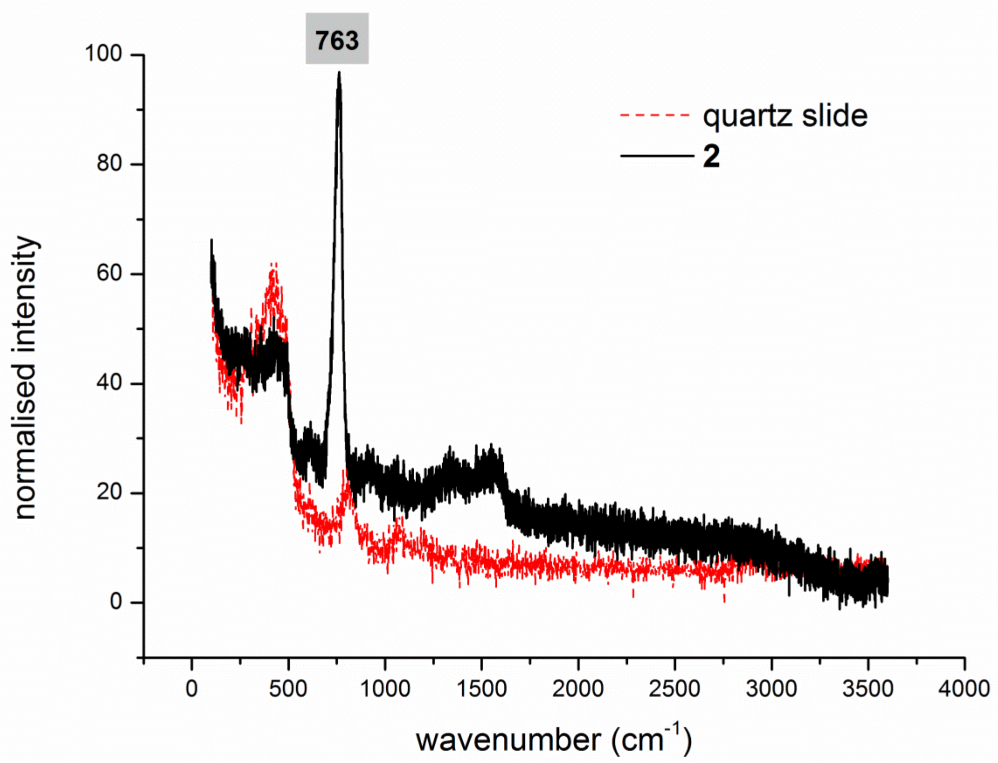

Figure S21. Raman spectrum of **2-crypt** (solid sample, quartz slide). The spectrum was acquired using a 532 nm laser, 2400 g/mm grating, 1 s exposure and 10 accumulations.

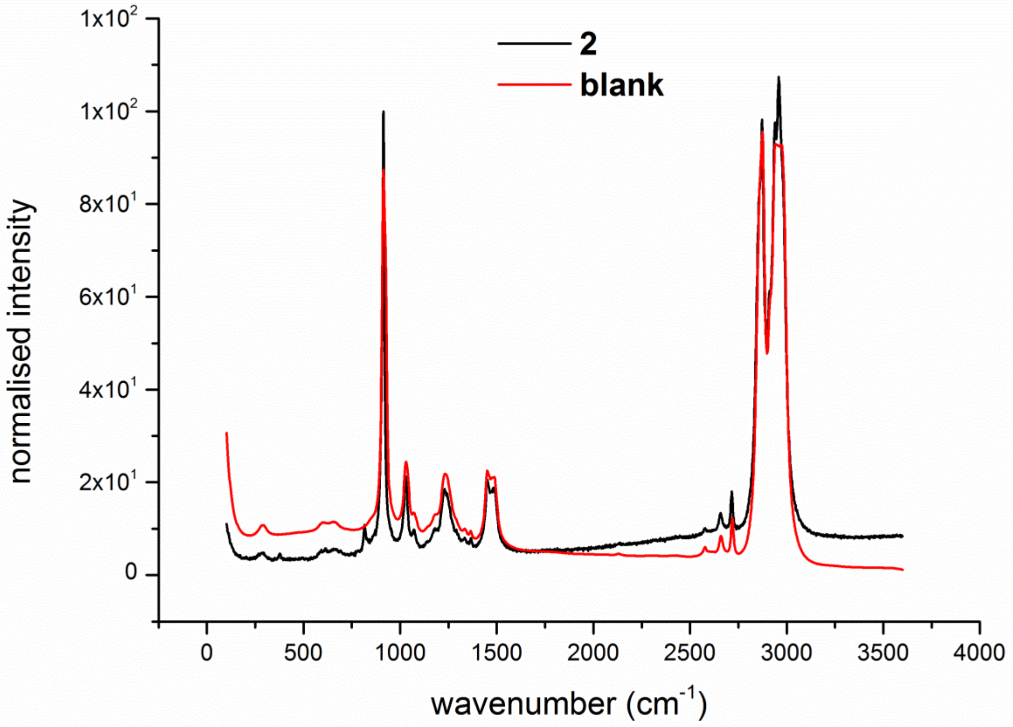

Figure S22. Raman spectrum of **2-crypt** (10mM in THF solution) with THF blank. The spectrum was acquired using a 532 nm laser, 2400 g/mm grating, 1 s exposure and 10 accumulations.

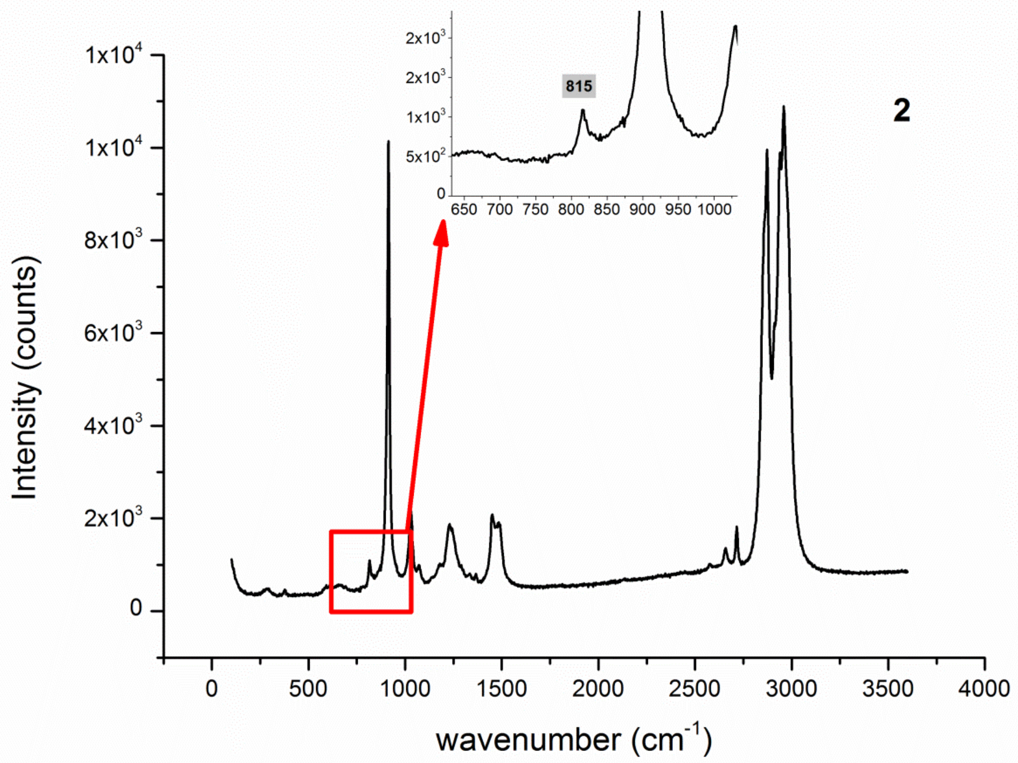

Figure S23. Raman spectrum of **2-crypt** (10mM in THF solution). The spectrum was acquired using a 532 nm laser, 2400 g/mm grating, 1 s exposure and 10 accumulations.

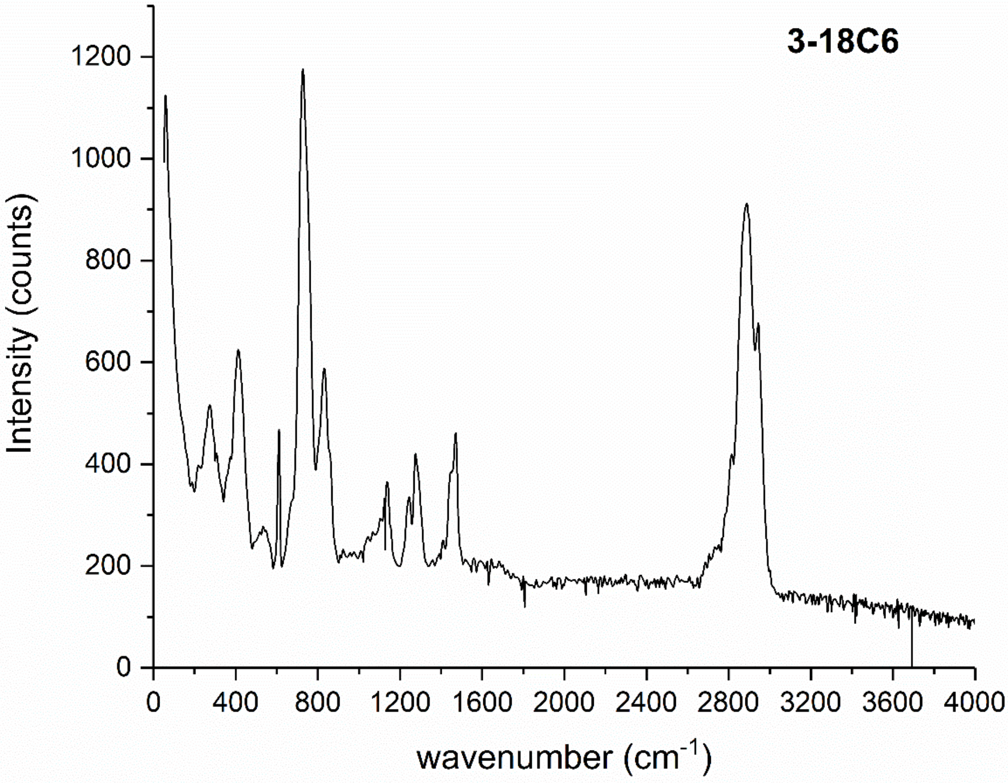

Figure S24. Raman spectrum of **3-18C6** (solid sample, quartz slide). The spectrum was acquired using a 638 nm laser, 1200 g/mm grating, 10 s exposure and 10 accumulations.

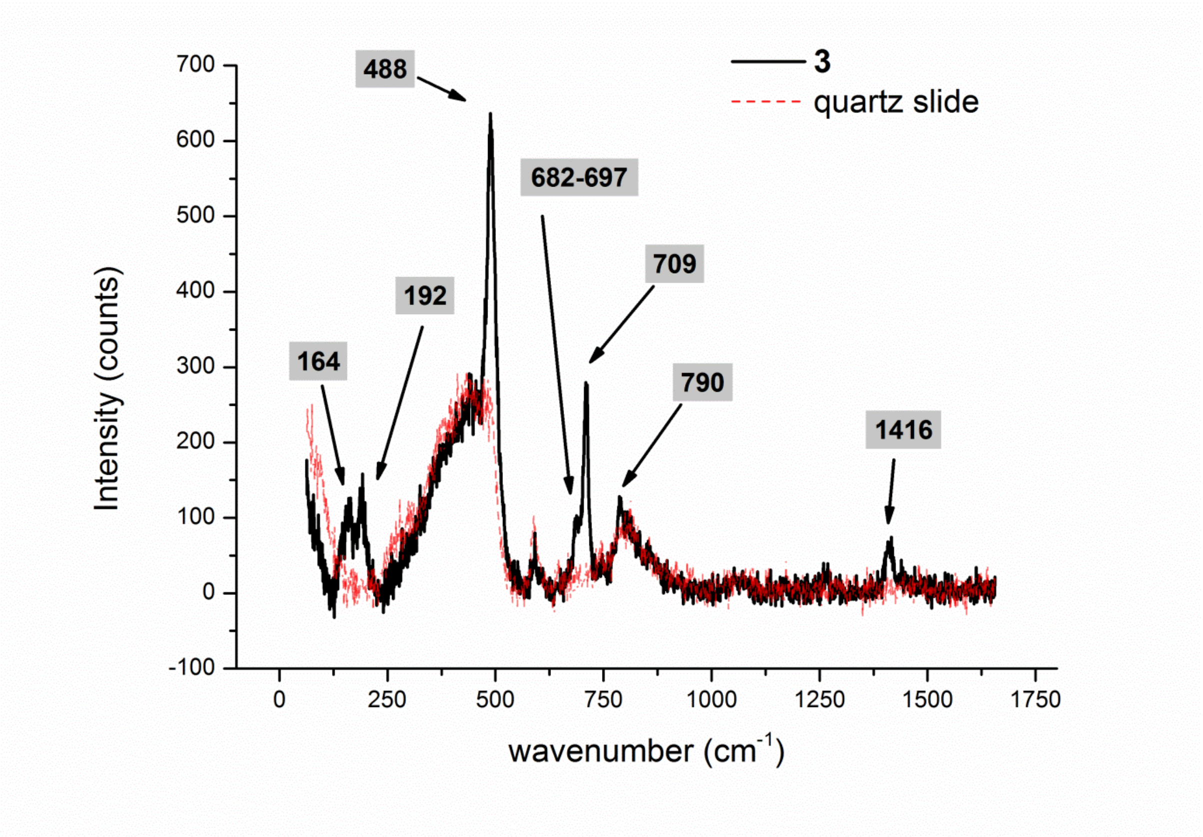

Figure S25. Raman spectrum of **3-crypt** (solid sample, quartz slide). The spectrum was acquired using a 532 nm laser, 1200 g/mm grating, 12 s exposure and 10 accumulations.

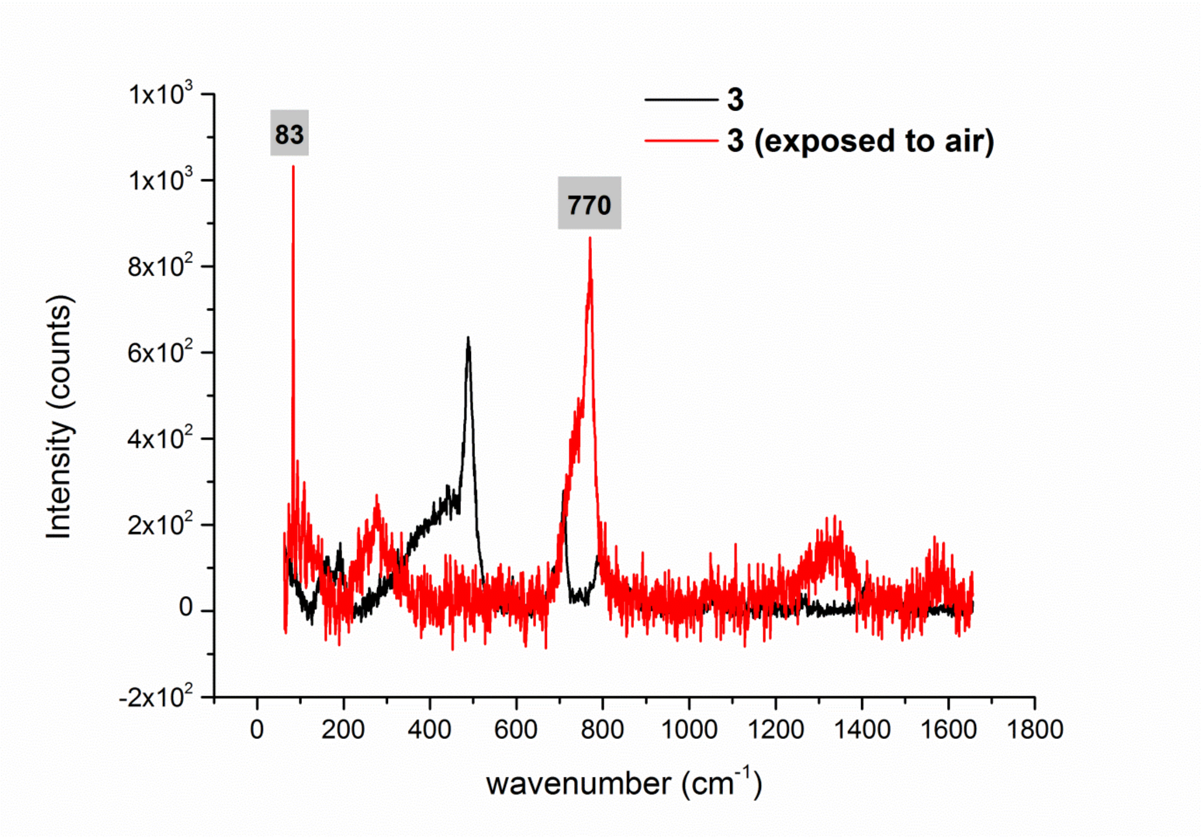

Figure S26. Raman spectrum of **3-crypt** after exposure to air (solid sample, quartz slide). The spectrum was acquired using a 532 nm laser, 1800 g/mm grating, 60 s exposure and 1 accumulation.

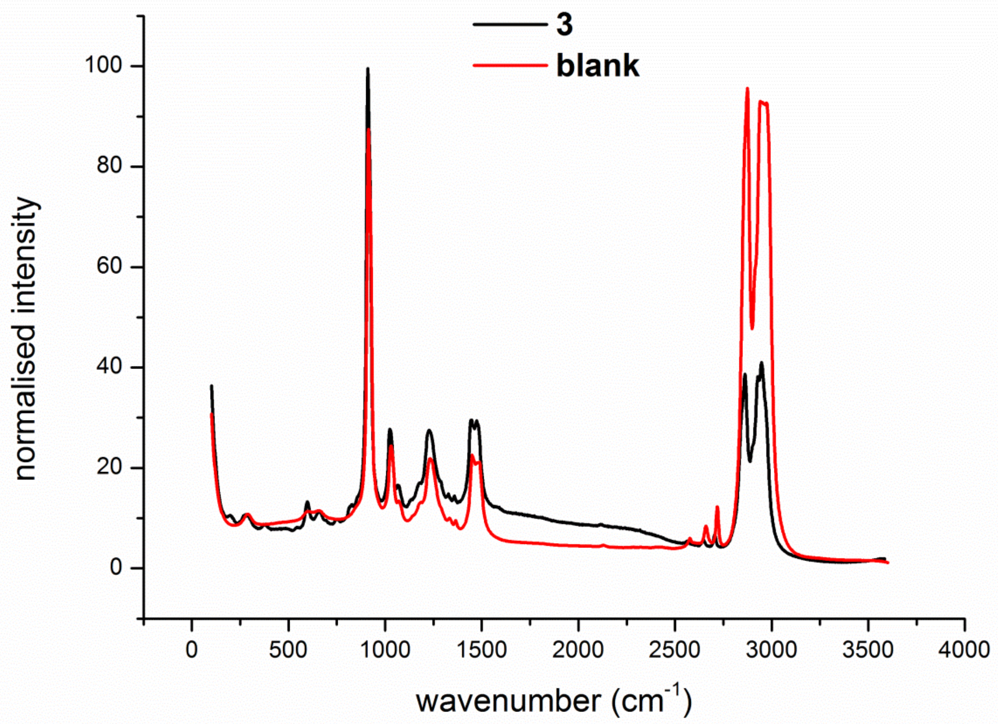

Figure S27. Raman spectrum of **3-crypt** (50 mM in THF solution) with THF blank. The spectrum was acquired using a 532 nm laser, 2400 g/mm grating, 10 s exposure and 20 accumulations.

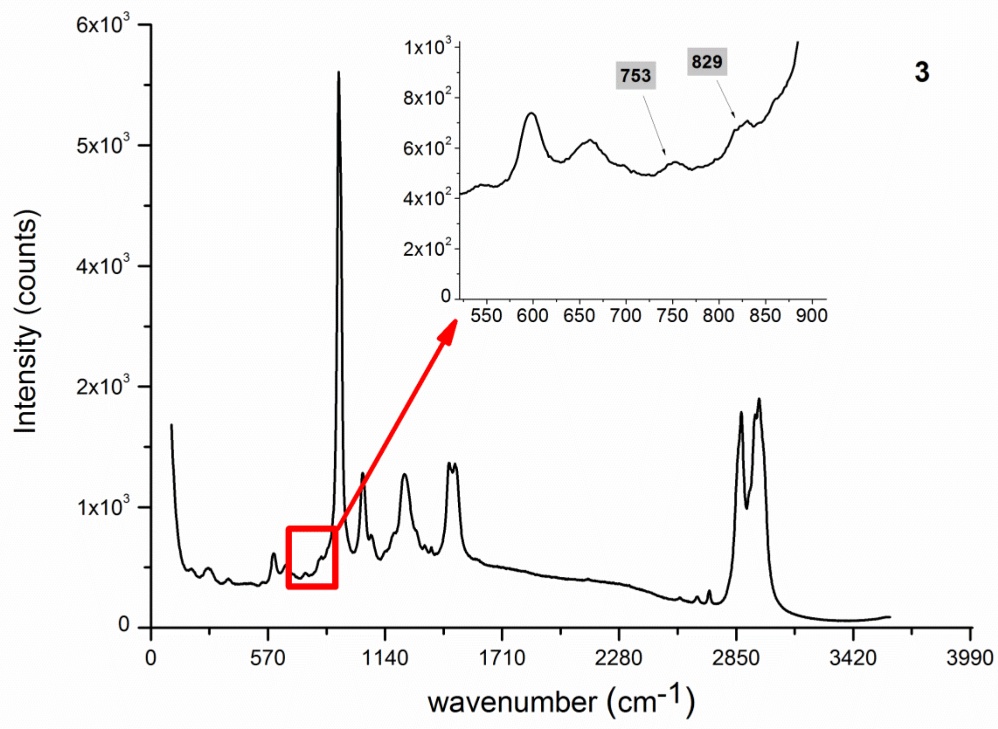

Figure S28. Raman spectrum of **3-crypt** (50 mM in THF solution). The spectrum was acquired using a 532 nm laser, 1200 g/mm grating, 10 s exposure and 20 accumulations.

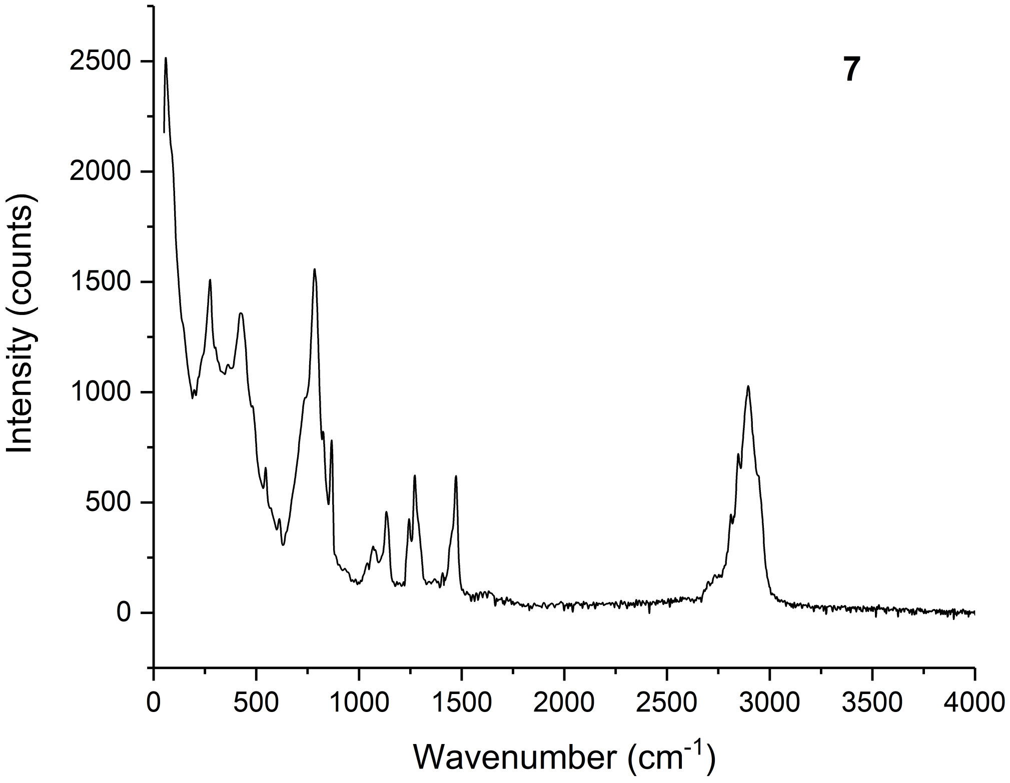

Figure S29. Raman spectrum of **7** (solid sample, quartz slide). The spectrum was acquired using a 638 nm laser, 1200 g/mm grating, 20 s exposure and 10 accumulations.

4. Crystallographic data

General methods

The crystal data for complexes **2-18C6-CHCl$_3$**, **2-18C6-THF**, **2-18C6-Tol**, **2-crypt-DME**, **2-crypt-Tol**, **3-crypt**, **3-crypt-DME**, **5**, **6** and **7** are compiled in Tables S1-5. Crystals of **2-18C6-CHCl$_3$**, **2-18C6-THF**, **2-18C6-Tol**, **2-crypt-DME**, **2-crypt-Tol**, **3-crypt**, **5-7** were examined using an Agilent Supernova diffractometer, equipped with CCD area detector and mirror-monochromated Mo Kα radiation (λ = 0.71073 Å). Crystals of **3-18C6** were examined using a Rigaku XtalLab AFC11 diffractometer, equipped with CCD area detector and mirror-monochromated Mo Kα radiation (λ = 0.71073 Å). Crystals of **3-crypt-DME** were examined using an Xcalibur Oxford Diffraction diffractometer, equipped with CCD area detector and mirror-monochromated Mo Kα radiation (λ = 0.71073 Å). Intensities were integrated from data recorded on 1° or 0.3° (**2-crypt-Tol**, **3-crypt-DME** and **5**) frames by ω rotation. Cell parameters were refined from the observed positions of all strong reflections in each data set. A Gaussian grid face-indexed absorption correction with a beam profile was applied.[2] The structures were solved variously by direct and heavy atom methods using SHELXS,[3] SHELXT[4] or Superflip (**3-crypt-DME**)[5] and were refined by full-matrix least-squares on all unique F^2 values,[3] with anisotropic displacement parameters for all non-hydrogen atoms, and with constrained riding hydrogen geometries; U_{iso}(H) was set at 1.2 (1.5 for methyl groups) times U_{eq} of the parent atom. The largest features in final difference syntheses were close to heavy atoms and were of no chemical significance. CrysAlisPro[2] was used for control and integration, and SHELX[3] was employed through OLEX2[6] for structure solution and refinement. ORTEP-3[7] and POV-Ray[8] were employed for molecular graphics. CCDC 1944749-1944759 contain the

supplementary crystal data for this article. These data can be obtained free of charge from the Cambridge Crystallographic Data Centre via www.ccdc.cam.ac.uk/data_request/cif.

Table S1. Crystallographic data for new polymorphs of **2-crypt**.

	**2-crypt-DME**	**2-crypt-Tol**	**2-18C6-CHCl$_3$**	**2-18C6-THF**	**2-18C6-Tol**
Formula	$C_{40}H_{100}KN_5O_{10}Si_6U$	$C_{57}H_{114}KN_5O_8Si_6U$	$C_{31}H_{79}Cl_3KN_3O_8Si_6U$	$C_{38}H_{94}KN_3O_{10}Si_6U$	$C_{37}H_{86}KN_3O_8Si_6U$
Fw	1256.91	1443.21	1173.99	1198.83	1146.75
cryst size, mm	0.09 x 0.18 x 0.26	0.15 x 0.15 x 0.18	0.25 x 0.26 x 0.46	0.25 x 0.39 x 0.44	0.15 x 0.17 x 0.32
crystal syst	monoclinic	trigonal	monoclinic	monoclinic	monoclinic
space group	$P2_1/n$	$R32$	$P2_1/c$	$C2/c$	$P2_1/c$
a, Å	12.8876(5)	18.9654(4)	12.1044(2)	24.9859(7)	15.2448(4)
b, Å	23.5867(12)	18.9654(4)	20.7648(4)	15.2204(4)	16.2455(4)
c, Å	20.9639(8)	36.1662(6)	21.7370(4)	17.0218(15)	23.2188(8)
α, °	90	90	90	90	90
β, °	103.830(4)	90	95.032(2)	108.589(3)	102.482(3)
γ, °	90	120	90	90	90
V, Å^3	6187.8(5)	11265.7(6)	5442.4(2)	9135.6(3)	5614.4(3)
Z	4	6	4	4	4
ρ_{calcd}, g mL	1.349	1.276	1.433	1.298	1.357
μ, mm^{-1}	2.854	2.359	3.379	2.874	3.135
$F(000)$	2600	4500	2384	2472	2352
no. of reflections (unique)	28023(11312)	36081(7034)	28749(12514)	13740(6980)	24252(12847)
S^a	1.03	1.05	1.02	1.03	1.00
$R_1(wR_2)$ ($F^2 > 2\sigma(F^2)$)	0.0628(0.1017)	0.0295(0.0876)	0.0305(0.0717)	0.0293(0.0635)	0.0470(0.0777)
R_{int}	0.080	0.034	0.028	0.025	0.045
min./max. diff map, Å^{-3}	−0.86, 1.22	−0.50, 1.25	–0.88, 1.23	–0.58, 0.74	–0.89, 1.26

^{a} Conventional $R = \Sigma||F_o| - |F_c||/\Sigma|F_o|$; $R_w = [\Sigma w(F_o^2 - F_c^2)^2/\Sigma w(F_o^2)^2]^{1/2}$; $S = [\Sigma w(F_o^2 - F_c^2)^2/\text{no. data} - \text{no. params})]^{1/2}$ for all data.

Table S2. Crystallographic data for **3-crypt**, **3-crypt-DME** and **3-18C6.**

	**3-crypt**	**3-crypt-DME**	**3-18C6**
Formula	$C_{54}H_{126}K_2N_7O_{14}Si_6U$	$C_{122}H_{287}K_4N_{14}O_{35}Si_{12}U$	$C_{50}H_{122}K_2N_3O_{18}Si_6U$
Fw	1582.38	3480.18	1538.27
cryst size, mm	0.09 x 0.11 x 0.14	0.48 x 0.60 x 1.17	0.01 x 0.01 x 0.01
crystal syst	monoclinic	triclinic	monoclinic
space group	$P2_1/n$	$P{-}1$	$P2_1/n$
a, Å	15.4763(5)	17.3637(2)	15.0503(2)
b, Å	20.3621(6)	20.2872(3)	19.8235(3)
c, Å	25.1249(6)	26.6771(4)	25.7902(4)
α, °	90	104.2892(13)	90
β, °	93.017(3)	94.9503(12)	97.613(1)
γ, °	90	100.0024(12)	90
V, Å^3	7906.6(4)	8885.6(2)	7626.67(19)
Z	4	2	4
ρ_{calcd}, g mL	1.329	1.301	1.340
μ, mm^{-1}	2.304	2.060	2.389
$F(000)$	3300	3650	3204
no. of reflections (unique)	32578(14450)	123910(49771)	88654(18900)
S^a	1.03	1.12	1.03
$R_1(wR_2)$ ($F^2 > 2\sigma(F^2)$)	0.0628(0.1017)	0.0365(0.0981)	0.0483(0.1001)
R_{int}	0.080	0.033	0.066
min./max. diff map, Å^{-3}	−0.86, 1.22	−1.21, 4.93	−1.70, 2.23

[a] Conventional $R = \Sigma||F_o| - |F_c||/\Sigma|F_o|$; $R_w = [\Sigma w(F_o^2 - F_c^2)^2/\Sigma w(F_o^2)^2]^{1/2}$; $S = [\Sigma w(F_o^2 - F_c^2)^2/\text{no. data} - \text{no. params})]^{1/2}$ for all data.

Table S3. Crystallographic data for **5**, **6** and **7**.

	**5**	**6**	**7**
Formula	$C_{60}H_{144}K_2N_8O_{16}Si_8U$	$C_{64}H_{152}K_2N_8O_{20}Si_8U$	$C_{40}H_{94}ClK_2N_2O_{16}Si_4U$
Fw	2012.80	2132.92	1323.21
cryst size, mm	0.09 x 0.16 x 0.22	0.11 x 0.16 x 0.33	0.11 x 0.18 x 0.23
crystal syst	monoclinic	monoclinic	orthorhombic
space group	$P2_1$	$P2_1/c$	$Pna2_1$
a, Å	12.3018(3)	15.0999(5)	21.4837(6)
b, Å	26.0487(6)	19.9619(6)	12.3630(4)
c, Å	14.7671(4)	33.5263(10)	23.4722(6)
α, °	90	90	90
β, °	99.686(2)	98.683(3)	90
γ, °	90	90	90
V, Å^3	4664.6(2)	9989.8(5)	6234.3(3)
Z	2	4	4
ρ_{calcd}, g mL	1.433	1.419	1.410
μ, mm^{-1}	3.715	3.476	2.913
F(*000*)	2044	4352	2716
no. of reflections (unique)	41613(18109)	50569(18252)	30070(12357)
S^a	1.00	1.00	1.00
$R_1(wR_2)$ ($F^2 > 2\sigma(F^2)$)	0.0662(0.1661)	0.0644(0.0893)	0.0404(0.0626)
R_{int}	0.0651	0.095	0.057
min./max. diff map, Å^{-3}	−3.85, 2.17	–1.51, 1.51	–0.54, 0.87

[a] Conventional $R = \Sigma||F_o| - |F_c||/\Sigma|F_o|$; $R_w = [\Sigma w(F_o^2 - F_c^2)^2/\Sigma w(F_o^2)^2]^{1/2}$; $S = [\Sigma w(F_o^2 - F_c^2)^2/\text{no. data} - \text{no. params})]^{1/2}$ for all data.

Table S4. Selected bond distances (Å) and angles (°) for **2-7**.

	**2-crown**	**3-18C6**	**3-crypt**	**3-crypt-DME**	**5**	**6**	**7**
U=O	1.773(4)-<br>1.804(4)	1.851(2)<br>1.855(2)	1.829(3)<br>1.843(3)	1.856(2)-<br>1.859(2)	1.845(13)<br>1.960(14)	1.776(5)<br>1.795(5)	1.846(5)-<br>1.848(5)
U−N	2.310(4)-<br>2.315(4)	2.448(3)-<br>2.466(3)	2.430(3)-<br>2.457(4)	2.455(3)-<br>2.476(3)	2.36(2)-<br>2.42(2)	2.358(6)-<br>2.376(6)	2.409(6)-<br>2.412(6)
O=U=O	179.51(16)	178.61(12)	178.06(12)	179.45(10)<br>179.52(9)	168.5(7)<br>168.8(7)	178.8(2)<br>179.6(2)	171.8(2)

Table S5. Selected bond distances (Å) and angles (°) for **5** and **6**.

	**5**	**6**
U=O	1.942(12), 1.960(14)<br>1.845(13), 1.866(12)	1.776(5)-1.794(5)
U−N	2.37 (2), 2.42(2)<br>2.36(2), 2.42(2)	2.358(6)-2.376(6)
O=U=O	168.8(7)<br>168.5(7)	179.6(2)<br>178.8(2)
U···O	2.324(13)<br>2.327(13)	

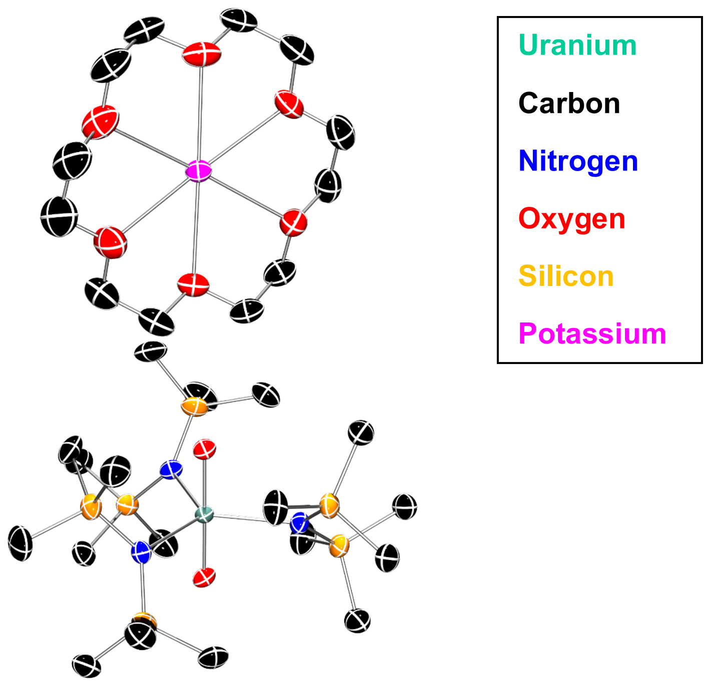

Figure S30: Molecular structure of **2-18C6-CHCl$_3$** with ellipsoids set at 50% probability level; hydrogens and lattice solvent ($CHCl_3$) have been omitted for clarity.

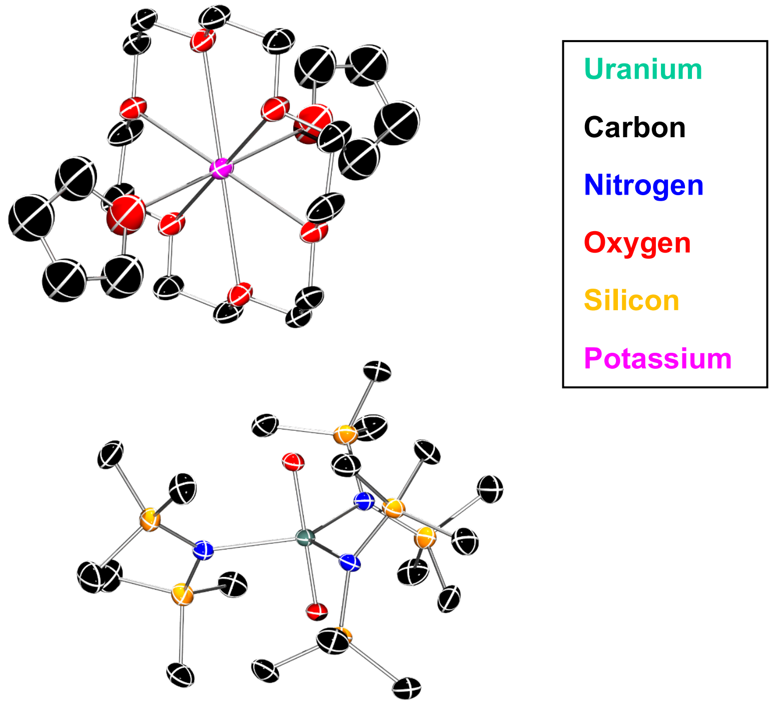

Figure S31: Molecular structure of **2-18C6-THF** with ellipsoids set at 50% probability level; hydrogens have been omitted for clarity.

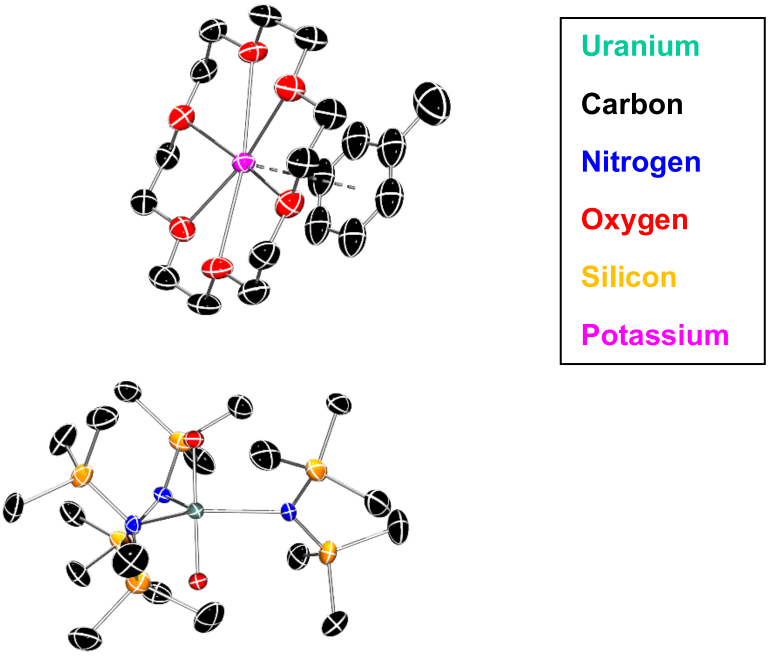

Figure S32: Molecular structure of **2-18C6-Tol** with ellipsoids set at 50% probability level; hydrogens have been omitted for clarity.

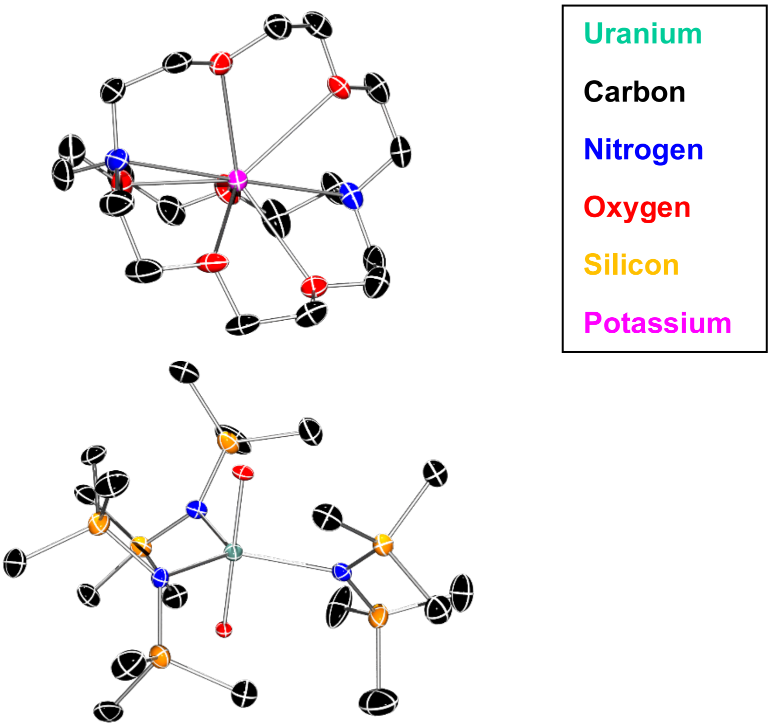

Figure S33: Molecular structure of **2-crypt-DME** with ellipsoids set at 50% probability level; hydrogens and lattice solvent (DME) have been omitted for clarity.

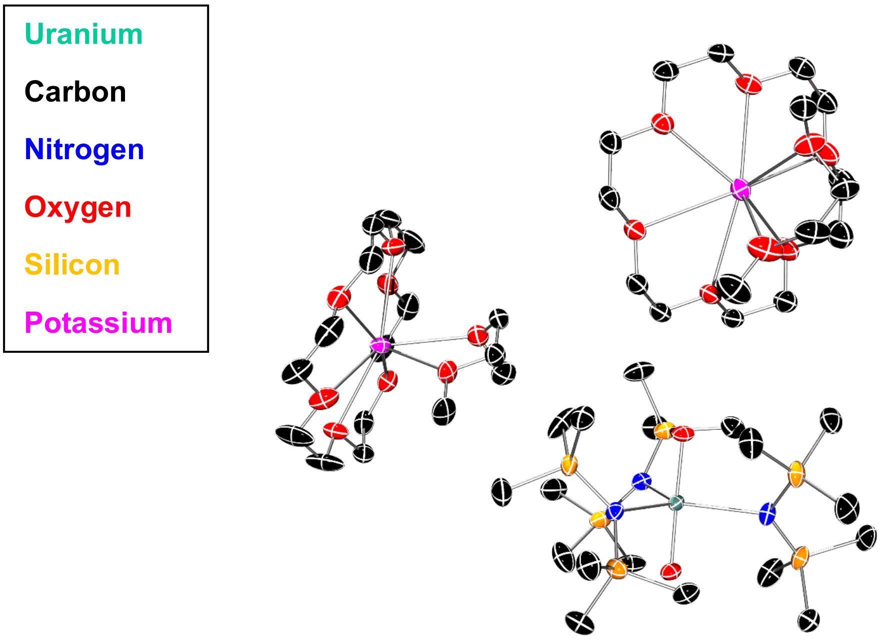

Figure S34: Molecular structure of **3-18C6** with ellipsoids set at 50% probability level; hydrogens have been omitted for clarity.

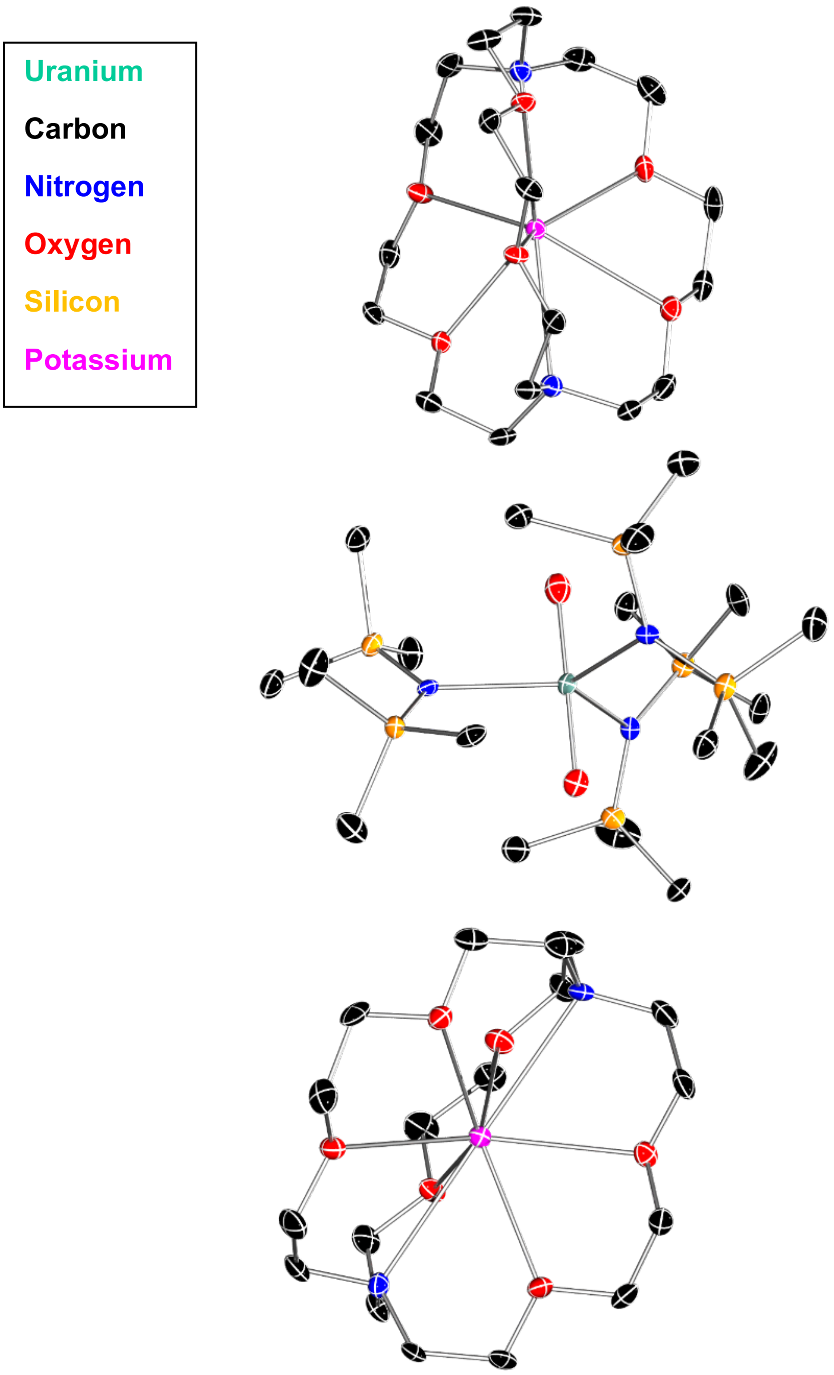

Figure S35: Molecular structure of **3-crypt** with ellipsoids set at 50% probability level; hydrogens have been omitted for clarity.

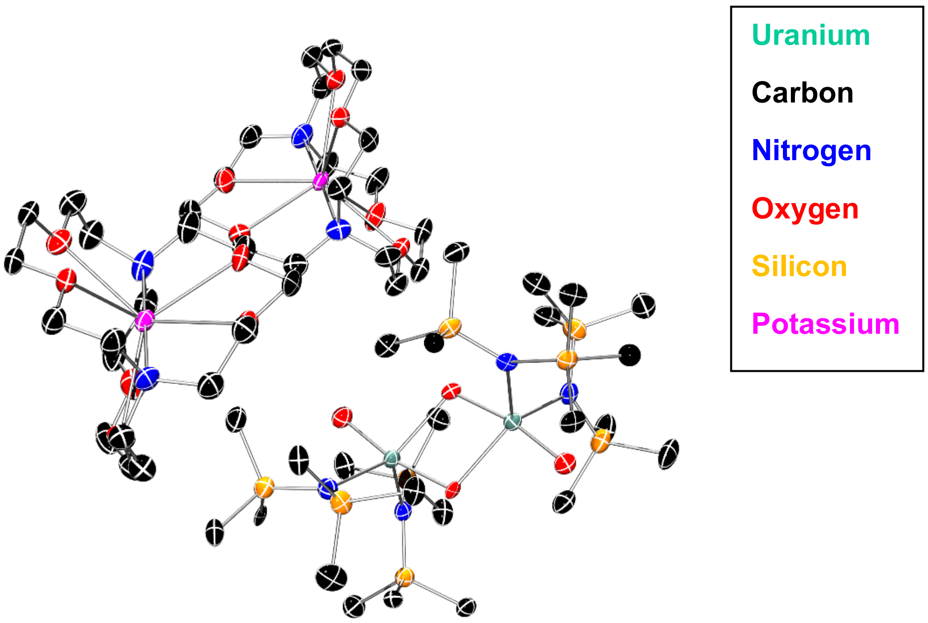

Figure S36: Molecular structure of **5** with ellipsoids set at 50% probability level; hydrogens have been omitted for clarity.

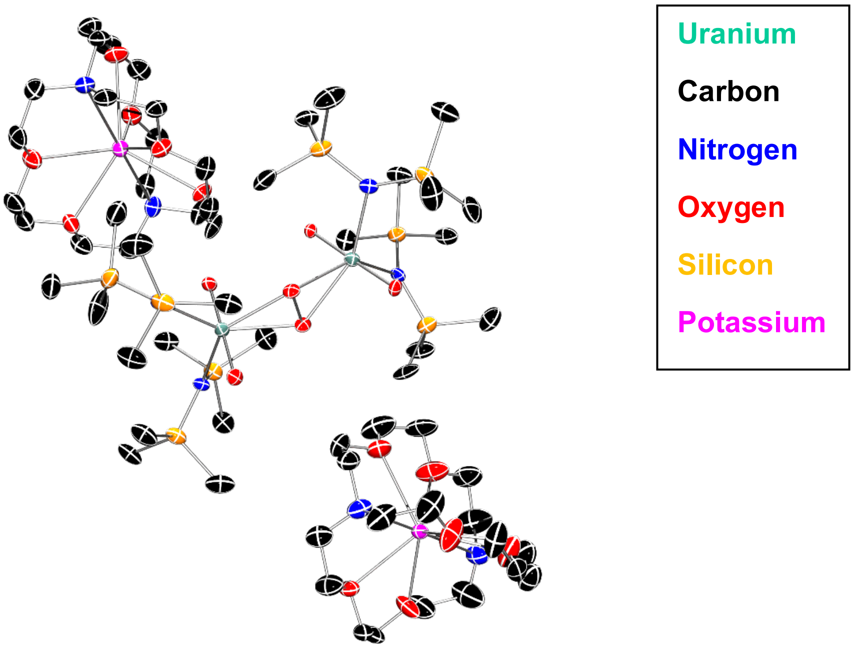

Figure S37: Molecular structure of **6** with ellipsoids set at 50% probability level; hydrogens have been omitted for clarity.

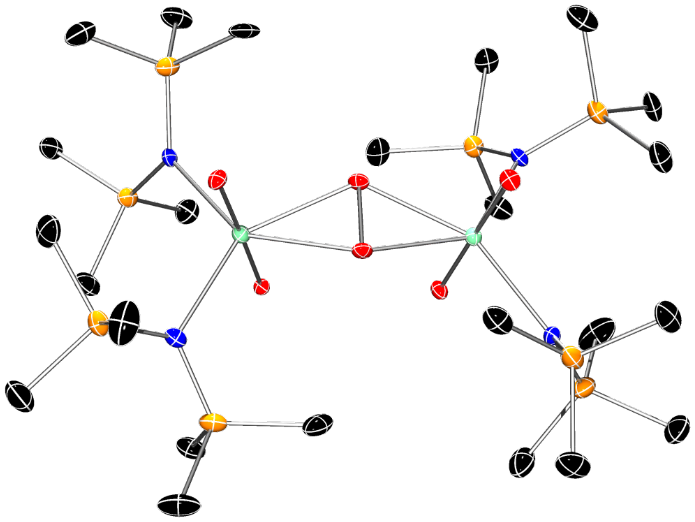

Figure S38: Molecular structure of **6** with ellipsoids set at 50% probability level; hydrogens and [K(2.2.2-cryptand)]$^{+}$ fragments have been omitted for clarity.

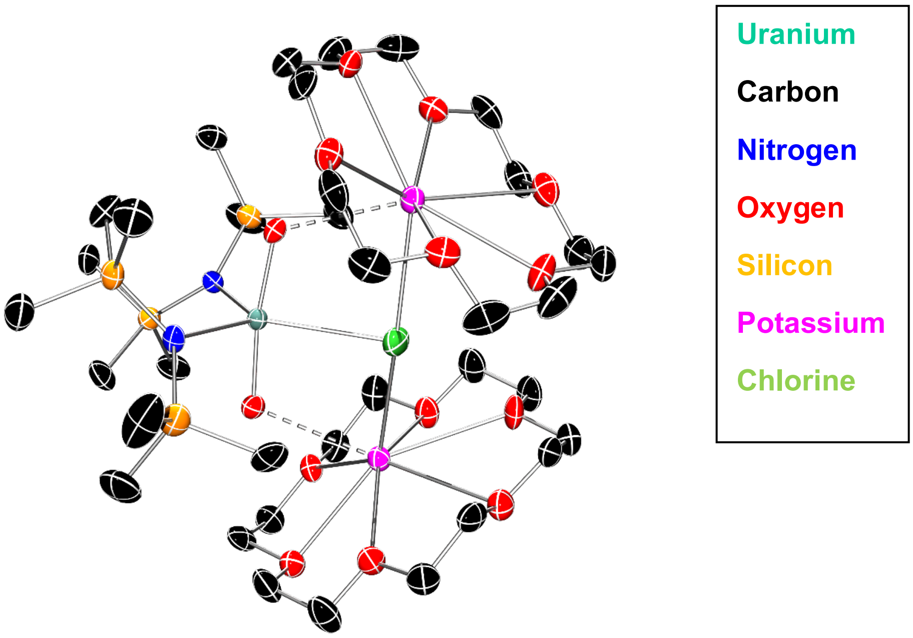

Figure S39: Molecular structure of **7** with ellipsoids set at 50% probability level; hydrogens have been omitted for clarity.

5. UV-vis-nIR Absorption Spectroscopy

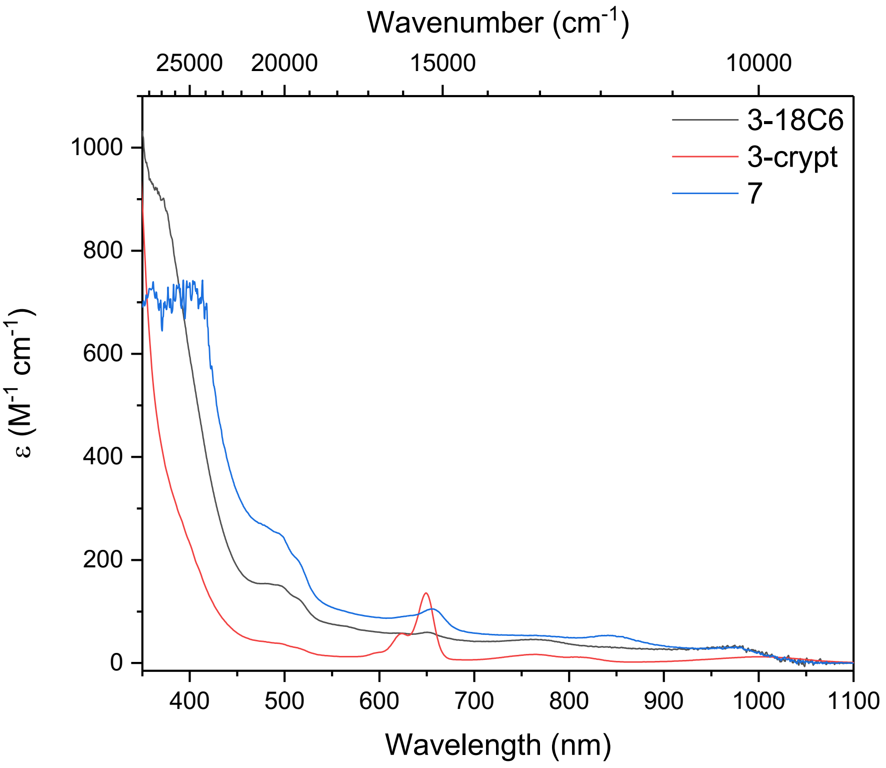

Figure S40: Electronic UV-vis-nIR absorption spectra of **3-18C6** (black trace), **3-crypt** (red trace) and **7** (blue trace) recorded in THF solution.

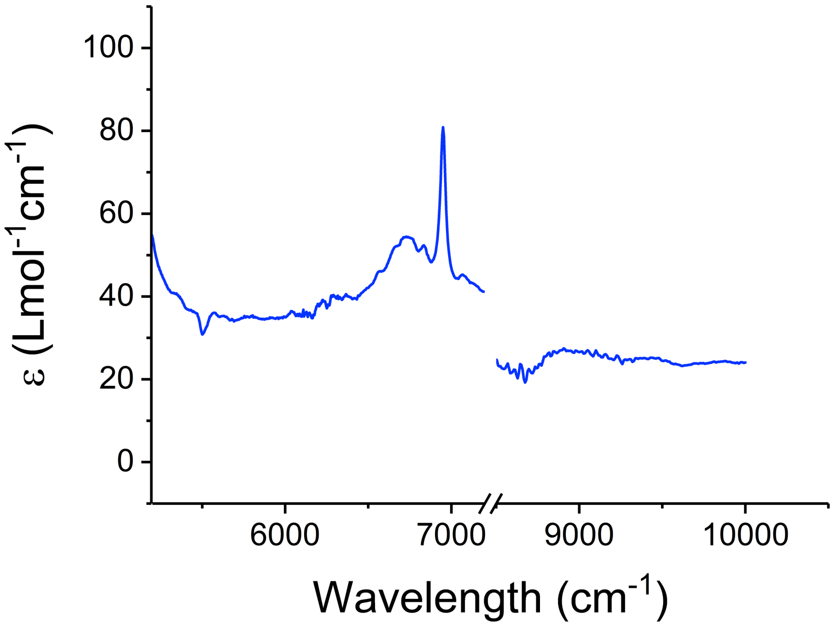

Figure S41. nIR absorption spectrum of **3-crypt** recorded in DME solution.

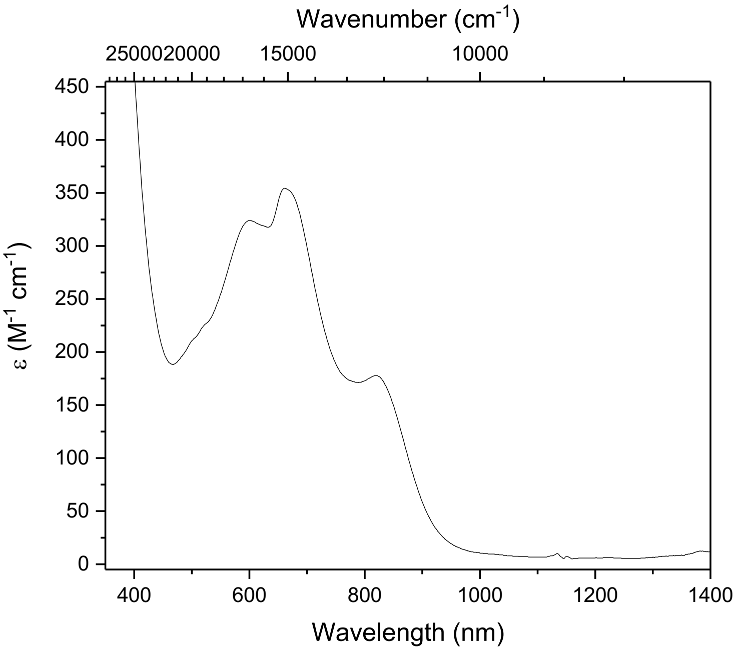

Figure S42. UV-vis-nIR absorption spectrum of **3-crypt** recorded in pyridine solution.

6. Luminescence Spectroscopy

Steady state emission and excitation spectra were recorded on an Edinburgh Instruments FP920 Phosphorescence Lifetime Spectrometer in Young's tap appended 1 cm quartz cuvettes equipped with a 450 W steady state xenon lamp, a 5 W pulsed xenon microsecond flash lamp and interchangeable EPL picosecond pulsed diode lasers as excitation sources, a red sensitive photomultiplier in Peltier (air cooled) housing (Hamamatsu R928P) and a liquid nitrogen cooled near infra-red photomultiplier (Hammamatsu) as detection sources. All spectra are reported corrected using the excitation and emission correction files provided with the instrument software. Frozen solution measurements were performed in Young's tap NMR tubes immersed in liquid nitrogen in a finger dewar and powdered samples were sandwiched between two 1 cm^2 quartz slides, sealed around the edges using vacuum grease. Lifetime data were recorded following excitation with either a 5 W pulsed xenon microsecond flash lamp or an EPL diode laser (375 nm) using time correlated single photon counting (PCS900 plug-in card for fast photon counting). Lifetimes were determined by tail fit on the data obtained and quality of fit judged by minimization of reduced χ-squared and residuals squared either using the instrument software or using the Origin software package. Unless otherwise stated, all spectral data presented were reproducible over at least three independently prepared samples from three separate syntheses over the 10^{-3} to 10^{-4} M concentration range.

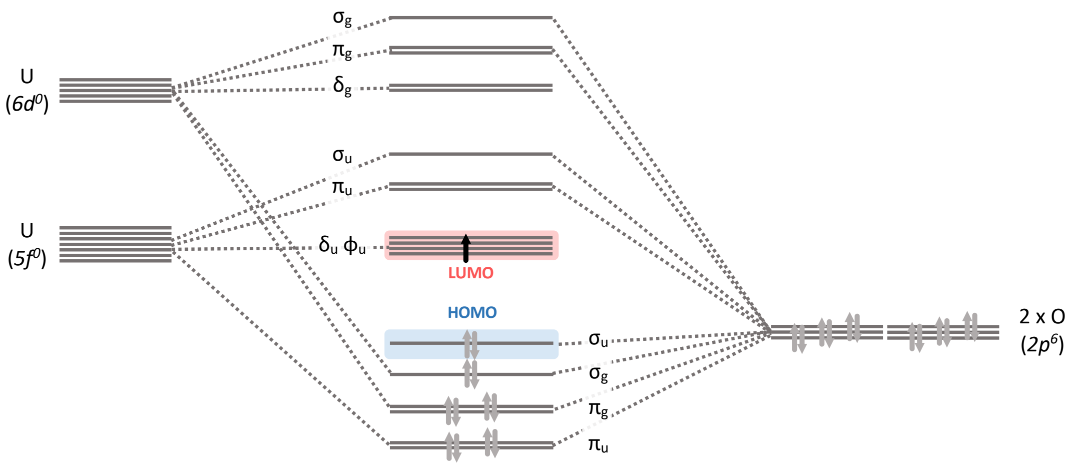

Figure S43. Molecular orbital (MO) diagram of the uranyl(VI) UO_2^{2+} unit in $D_{\infty}h$ point symmetry, redrawn according to Denning.[9] Note that the unoccupied MO's are near degenerate and their ordering is somewhat arbitrary in coordination compounds.

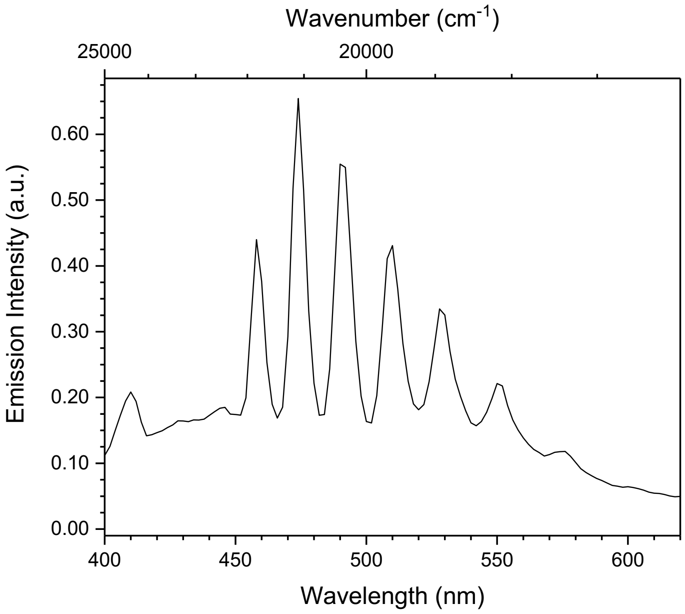

Figure S44. Emission spectrum of **3-crypt** from 400-600 nm following 280 nm excitation, focusing on the vibrationally resolved feature, 2-Me-THF, 77 K.

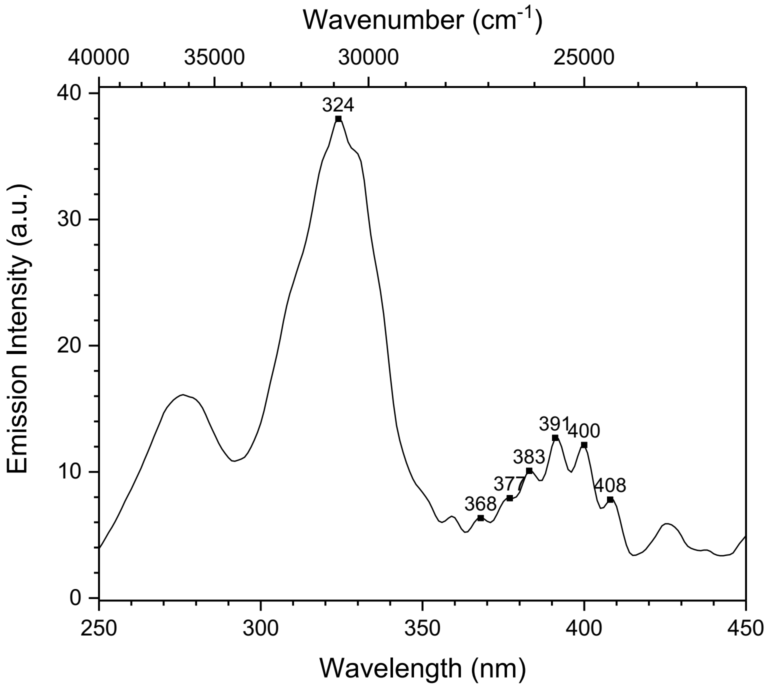

Figure S45. Excitation spectrum of **3-crypt** (475 nm emission) with peaks picked, 2-Me-THF, 77 K.

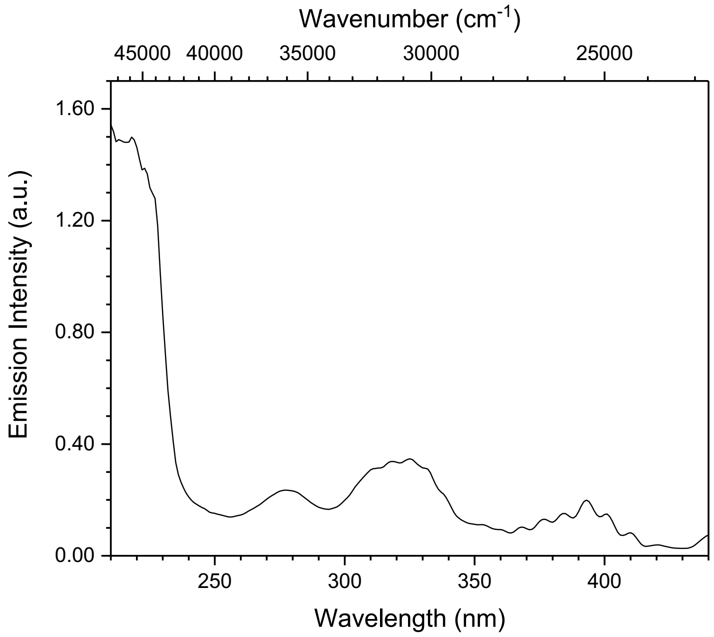

Figure S46. Full excitation spectrum of **3-crypt** (491 nm emission), 2-Me-THF, 77 K

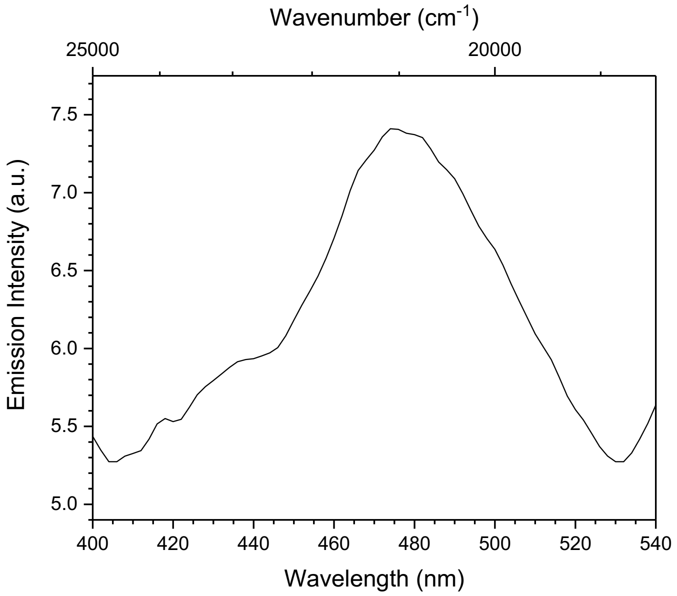

Figure S47. Expanded emission spectrum of **3-crypt** following 280 nm excitation, 2-Me-THF, 295 K. Note that the cropped feature from 530 nm onwards is due to the onset of double the excitation frequency.

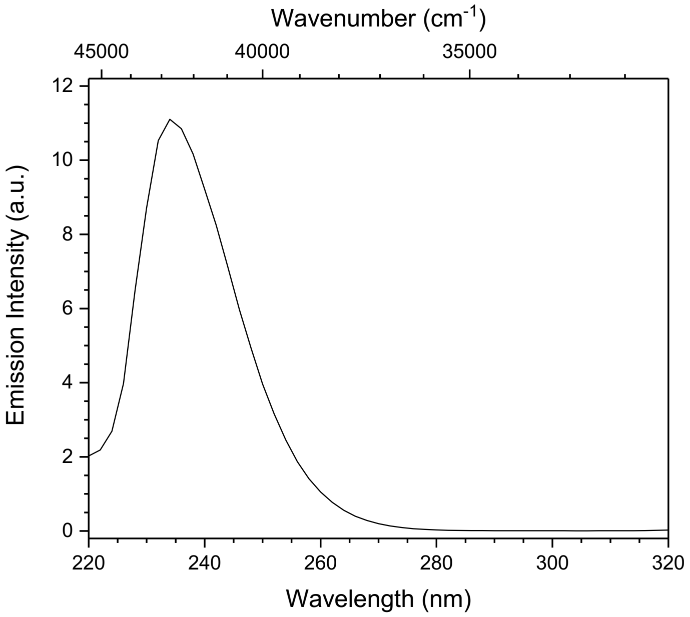

Figure S48. Excitation spectrum of **3-crypt** (360 nm emission) in 2-Me-THF at 295 K.

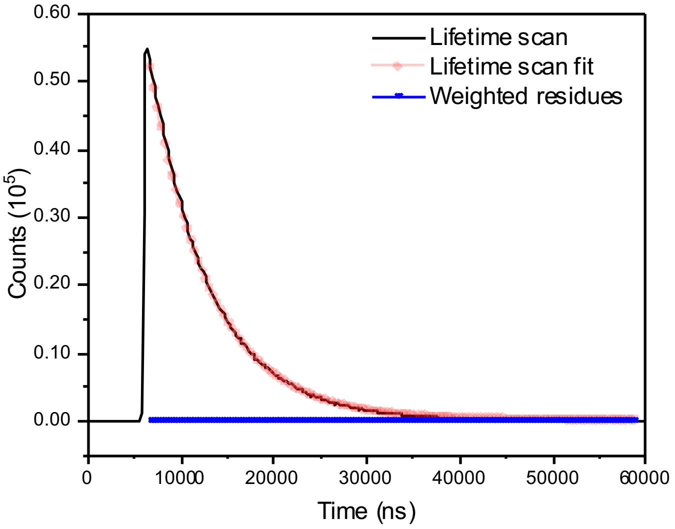

Figure S49. Fitted kinetic trace of the decay profile at 475 nm in **3-crypt** following 300 nm excitation, 2-Me-THF, 77 K.

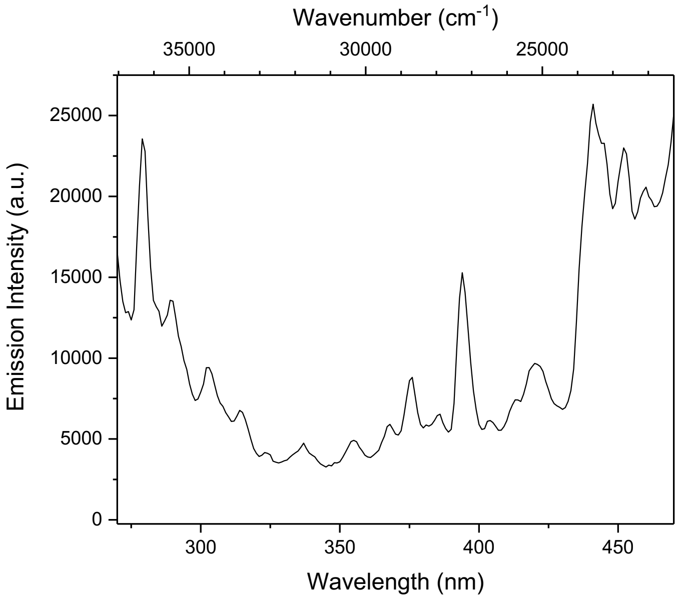

Figure S50. Excitation spectrum of powdered **3-crypt** recorded at 491 nm emission, 295 K. Note that the spectrum is dominated by scatter from the solid sample.

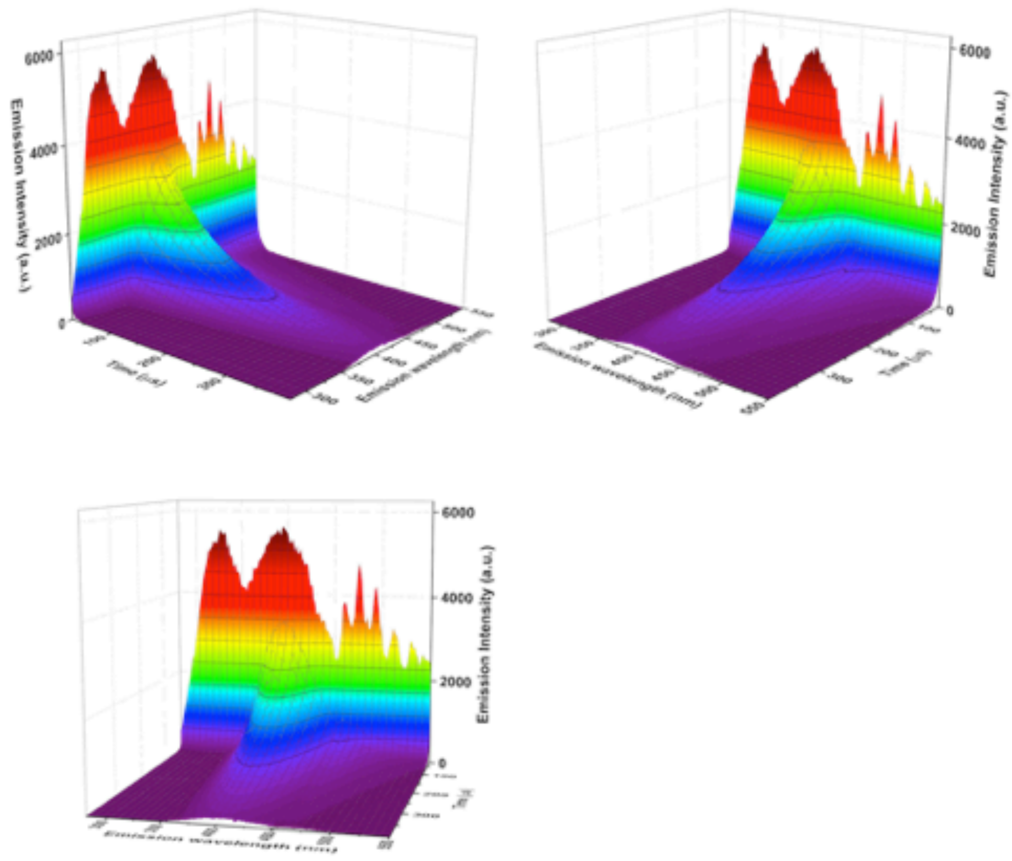

Figure S51. Time resolved emission spectra (showing different views and the long, weaker tail of the emission at 470 nm) of powdered **3-crypt** following 230 nm excitation at 295 K.

λ_{em} = 320 nm λ_{em} = 400 nm λ_{em} = 470 nm

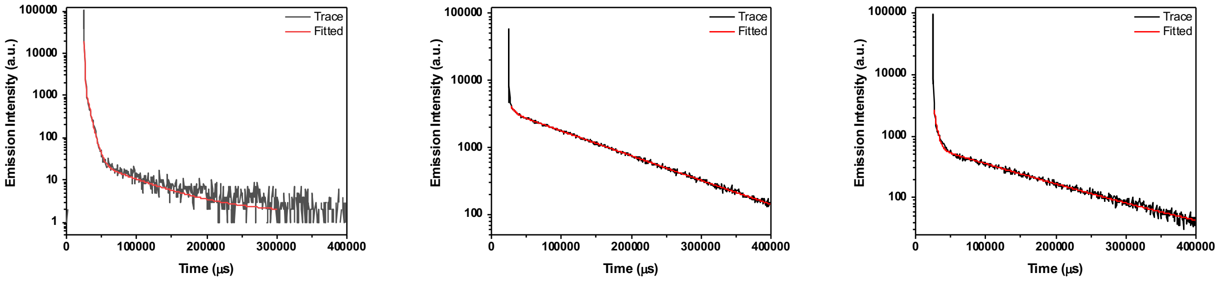

τ_1 = 0.53 μs (76%)
τ_2 = 5.38 μs (21%)
τ_3 = 63.9 μs (3%)
X^2 = 1.273

τ_1 = 5.56 μs (2%)
τ_2 = 117 μs (98%)
X^2 = 0.971

τ_1 = 4.31 μs (12%)
τ_2 = 127 μs (88%)
X^2 = 1.903

Figure S52. Representative fitted kinetic traces of powdered **3-crypt** at 320 nm emission, 400 nm emission and 470 nm emission following 230 nm excitation at 295 K. No significant improvement in fit was observed when a triexponential fit was applied to the data at 400 nm and 470 nm emission, whereas mono-exponential fits were not satisfactory for all kinetic traces and applying a biexponential fitting procedure to the data at 320 nm was unsatisfactory.

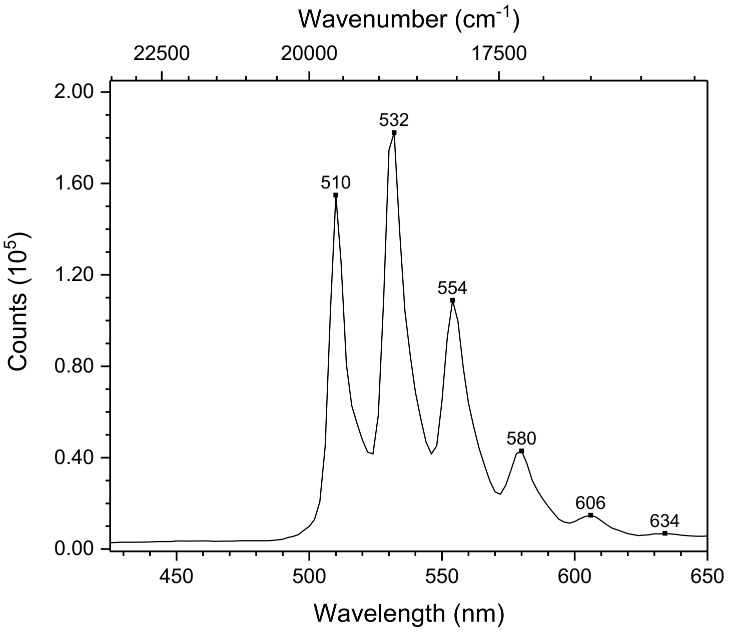

Figure S53. Emission spectrum of **3-crypt** after exposure to air and oxidation to uranyl(VI) following 280 nm excitation, 2-Me-THF, 77 K. The peak positions are 510 nm (19608 cm^{-1}), 532 nm (18797 cm^{-1}), 554 nm (18382 cm^{-1}), 580 nm (17241 cm^{-1}), 606 nm (16502 cm^{-1}) and 634 (15773 cm^{-1}); E_{0-0} = 811 cm^{-1}.

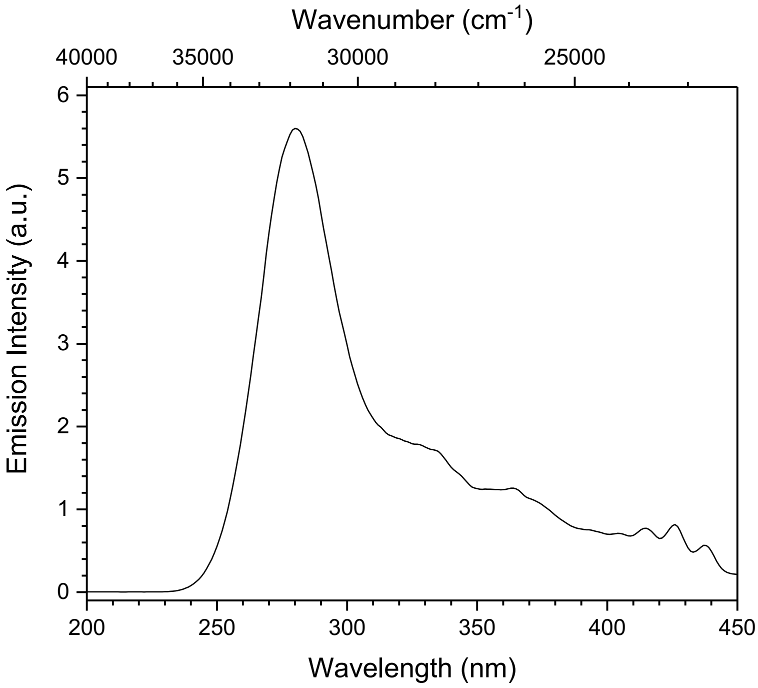

Figure S54. Excitation spectrum of **3-crypt** after exposure to air and oxidation to uranyl(VI) recorded at the emission at 532 nm, 2-Me-THF, 77 K. Note the characteristic vibrationally resolved uranyl(VI) U=O LMCT absorption centred at 425 nm.

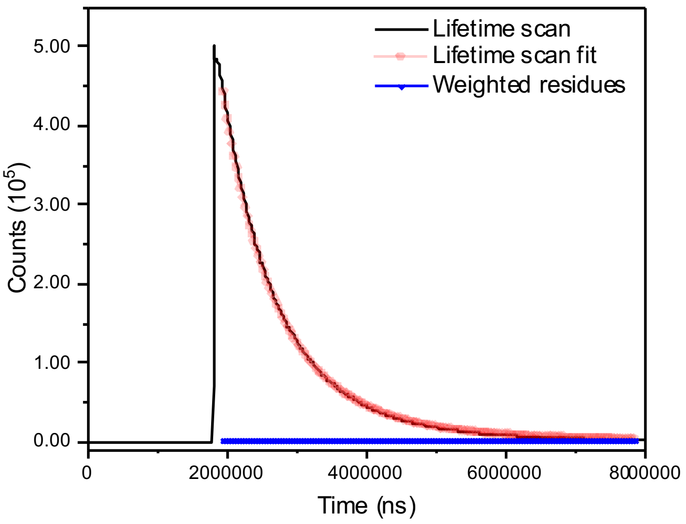

Figure S55. Fitted kinetic trace of the decay profile at 532 nm in **3-crypt** following exposure to air, 280 nm excitation, 2-Me-THF, 77 K.

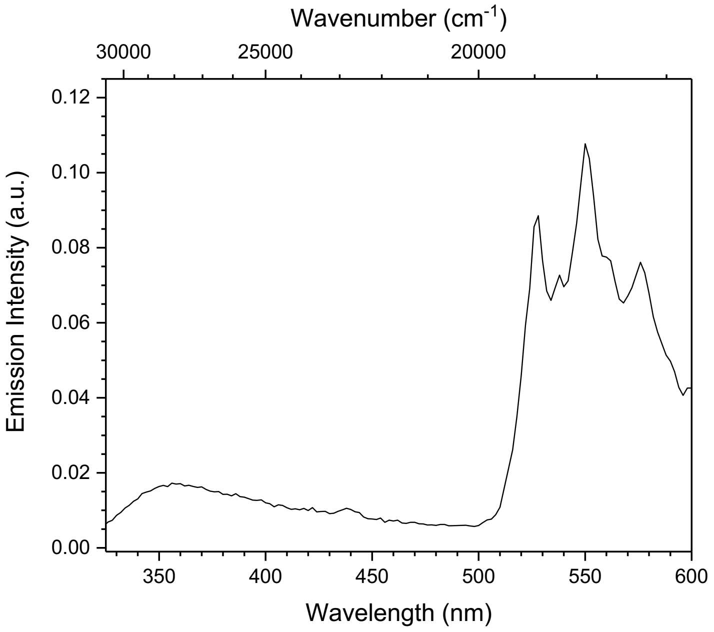

Figure S56. Emission spectrum of **2-crypt**, [K(2.2.2-cryptand)][UO_2{N($SiMe_3$)$_2$}$_3$] following 300 nm excitation, 2-Me-THF, 77 K. The principle peak positions are 528 nm (18939 cm^{-1}), 550 nm (18797 cm^{-1}), 554 nm (18182 cm^{-1}), 576 nm (17361 cm^{-1}), with vibrational spacings measured as 757 cm^{-1} and 821 cm^{-1}. Note that the emission maximum is considerably red shifted (550 nm) compared to aqueous uranyl salts (ca. 510 - 520 nm) due to the strong amide sigma donors located in the equatorial plane and the feature centered at 360 nm is attributed to residual equatorial amide to uranium LMCT emission.

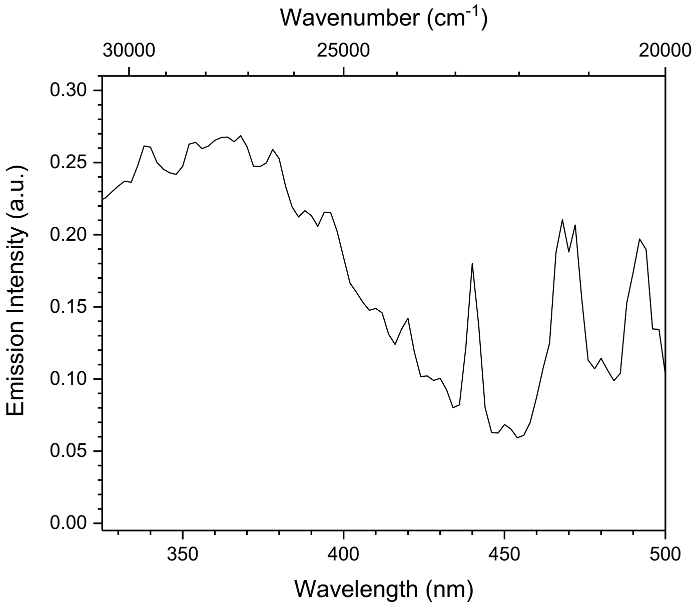

Figure S57. Excitation spectrum of **2-crypt**, [K(2.2.2-cryptand)][UO_2{N($SiMe_3$)$_2$}$_3$] recorded at the emission maximum (550 nm), 2-Me-THF, 77 K.

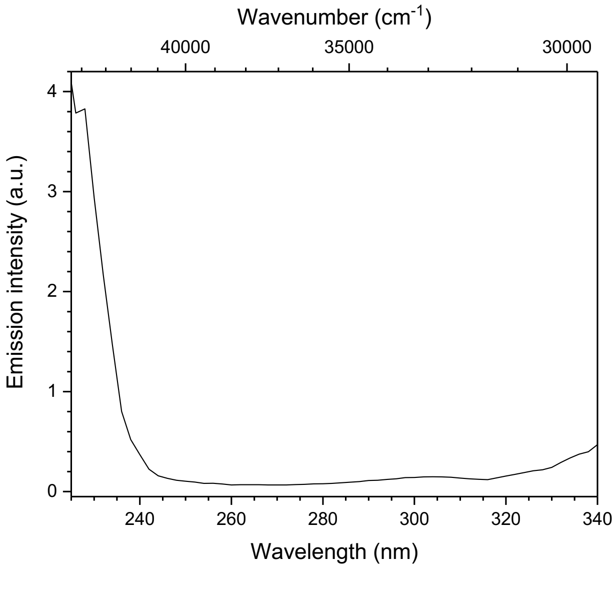

Figure S58. Excitation spectrum of **2-crypt**, [K(2.2.2-cryptand)][UO_2{N($SiMe_3$)$_2$}$_3$] recorded at 360 nm emission, 2-Me-THF, 77 K.

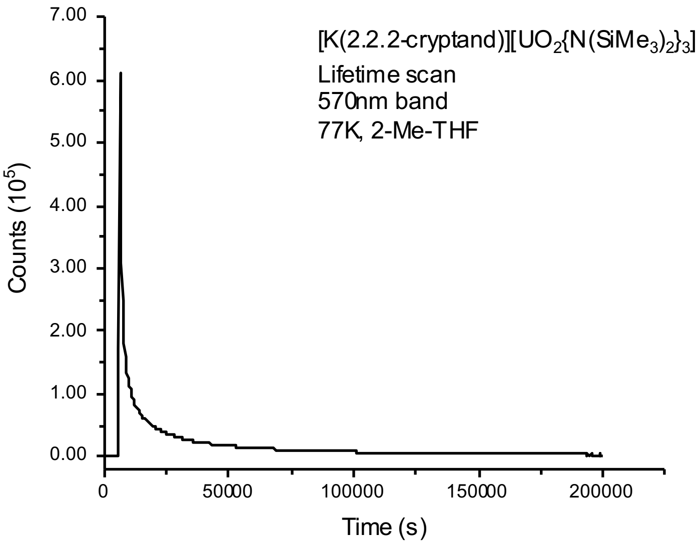

Figure S59. Kinetic trace of **2-crypt**, [K(2.2.2-cryptand)][UO_2{N($SiMe_3$)$_2$}$_3$] recorded at 570 nm emission, following 300 nm excitation, 2-Me-THF, 77 K.

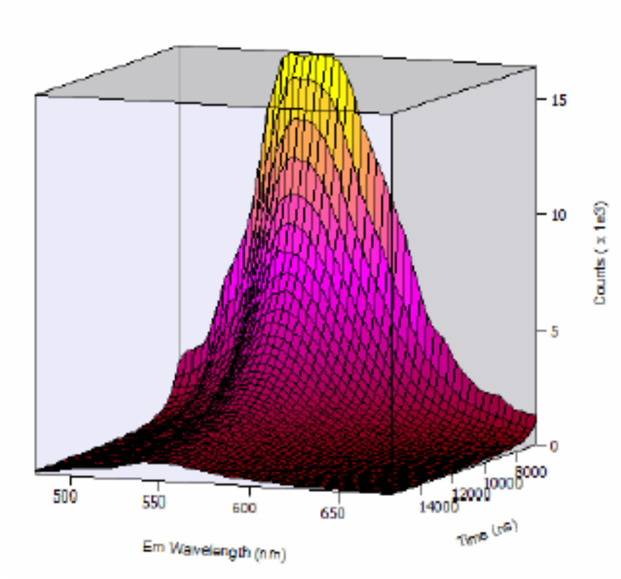

Figure S60. Left, time resolved emission spectrum of powdered **2-crypt** following 250 nm excitation, 295 K.

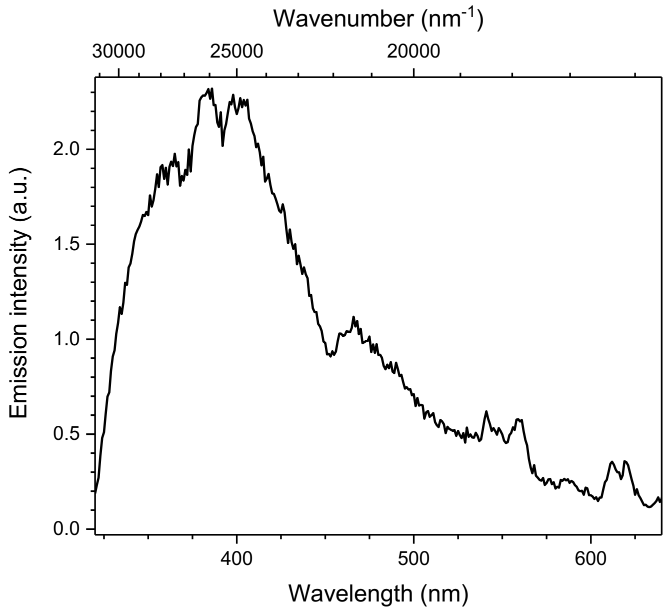

Figure S61. Emission spectrum of **5** following 280 nm excitation, DME, 77 K (315 nm long pass filter)

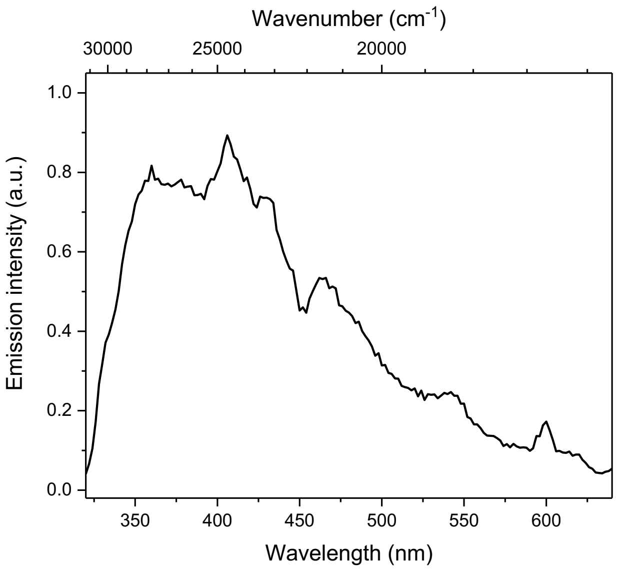

Figure S62. Emission spectrum of **5** following 300 nm excitation, DME, 77 K (315 nm long pass filter).

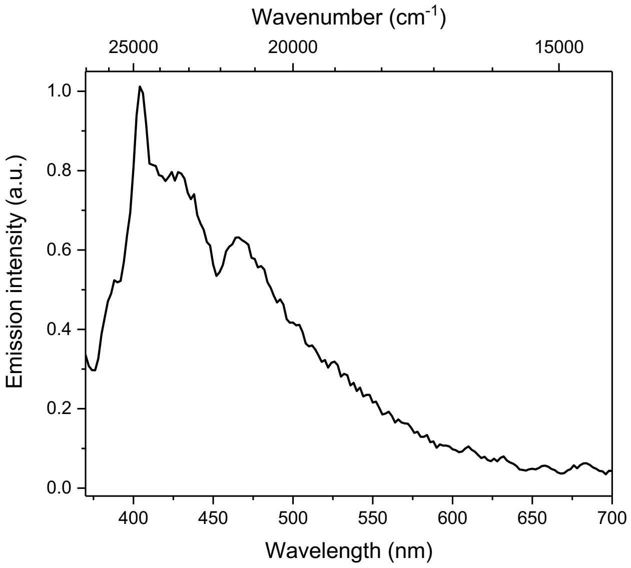

Figure S63. Emission spectrum of **5** following 360 nm excitation, DME, 77 K (375 nm long pass filter).

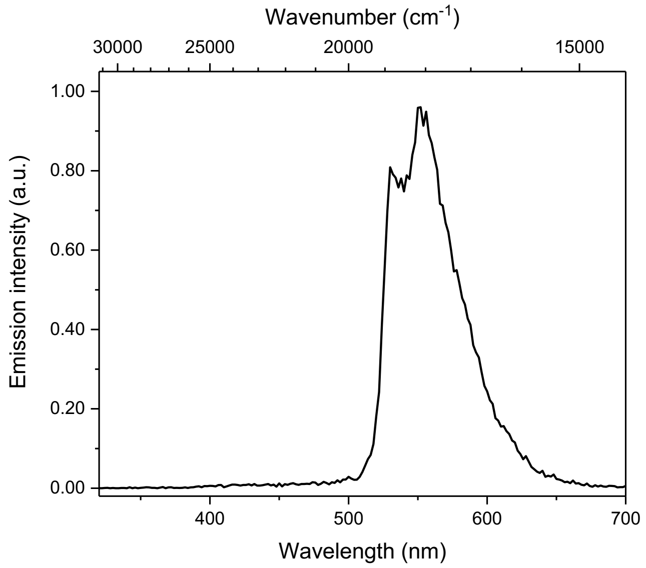

Figure S64. Emission spectrum of **5** after 20 minutes measurements, defrosted and re-frozen, 260 nm excitation, THF, 77 K, showing emission typical of uranyl(VI), suggesting decomposition/oxidation.

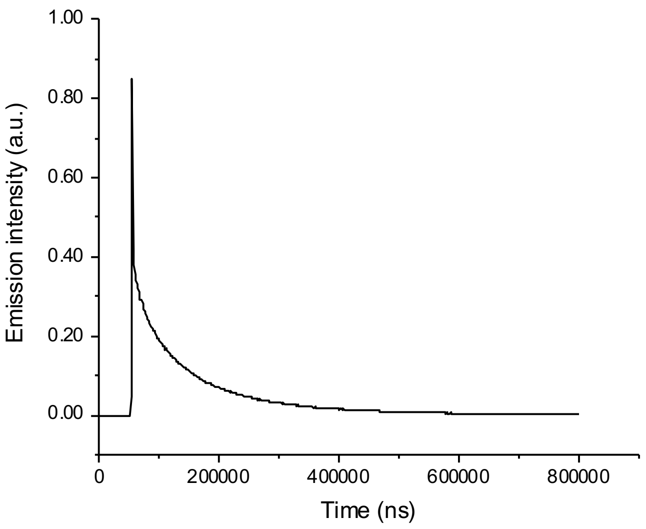

Figure S65. Kinetic trace of **5** at 535 nm emission following 260 nm pulsed excitation, DME, 77 K.

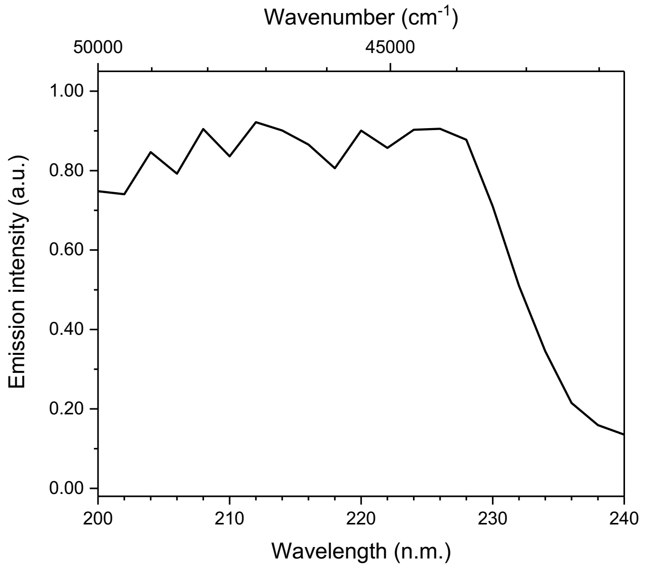

Figure S66. Excitation spectrum of **5** recorded at 520 nm emission, THF, 77 K

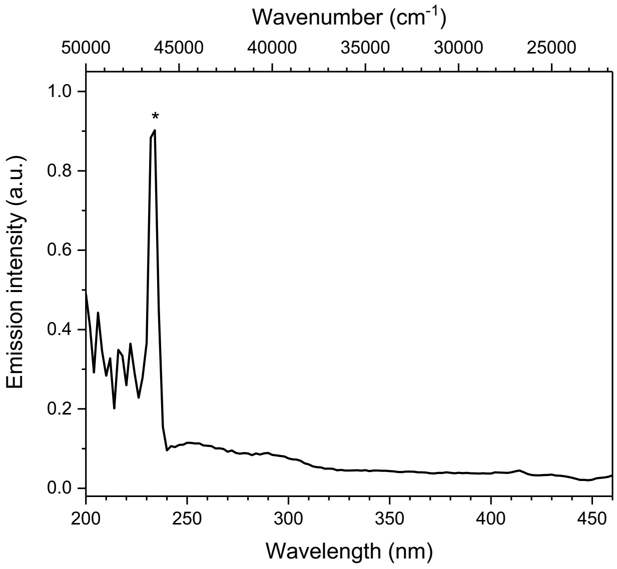

Figure S67. Excitation spectrum of **5** recorded at 470 nm emission, DME, 77 K (* denotes scattered light at double the emission frequency).

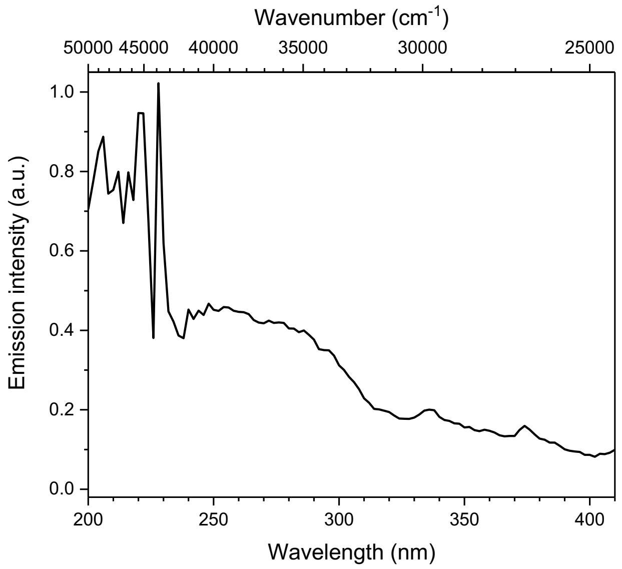

Figure S68. Excitation spectrum of **5** recorded at 420 nm emission, DME, 77 K.

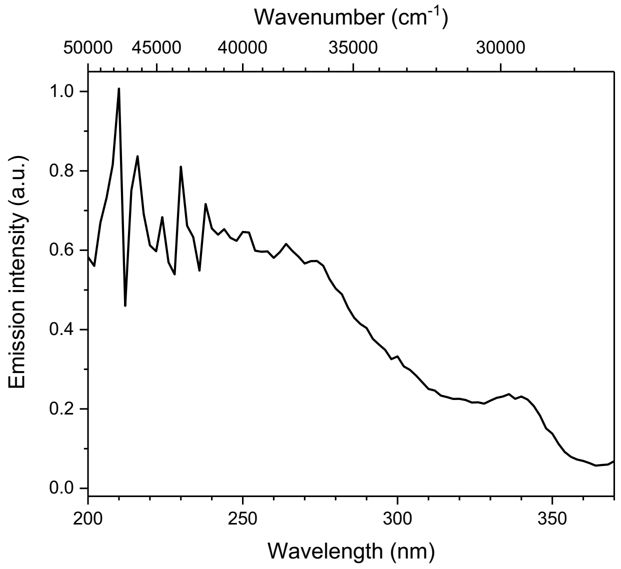

Figure S69. Excitation spectrum of **5** recorded at 360 nm emission, DME, 77 K.

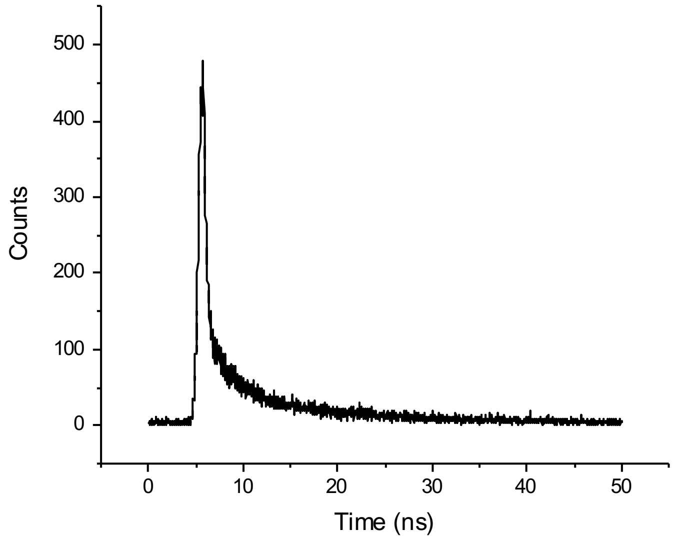

Figure S70. Kinetic trace of **5** at 420 nm emission following 280 nm pulsed excitation, DME, 77 K.

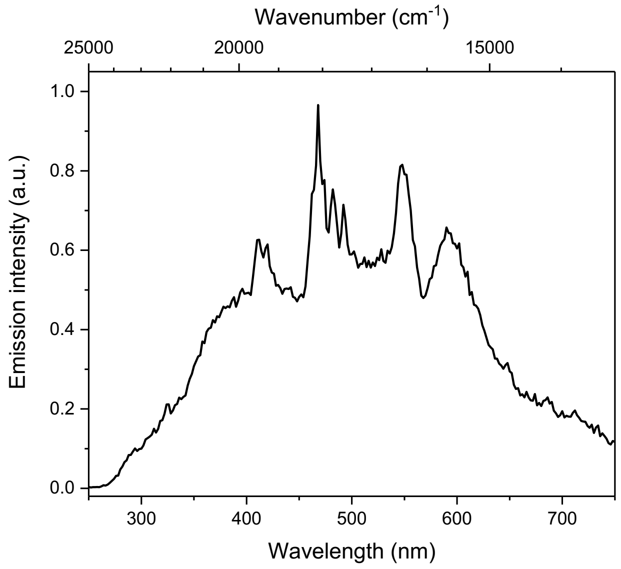

Figure S71. Emission spectrum of powdered **5** following 230 nm excitation, 295 K (245 nm long pass filter). The features after double the excitation wavelength (460 nm) are probably due to second order effects and therefore can be discounted.

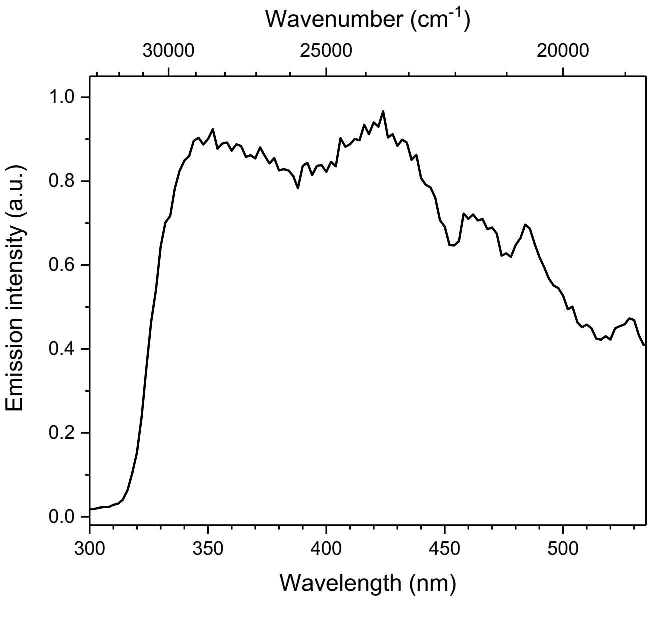

Figure S72. Emission spectrum of powdered **5** following 280 nm excitation, 295 K (315 nm long pass filter). The features after double the excitation wavelength (560 nm)

are likely due to second order effects and therefore have been omitted from the spectrum.

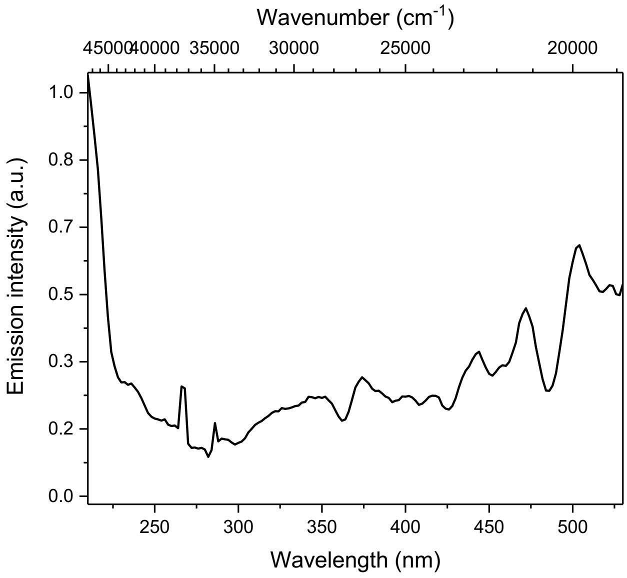

Figure S73. Excitation spectrum of powdered **5**, monitored at 550 nm emission, 295 K.

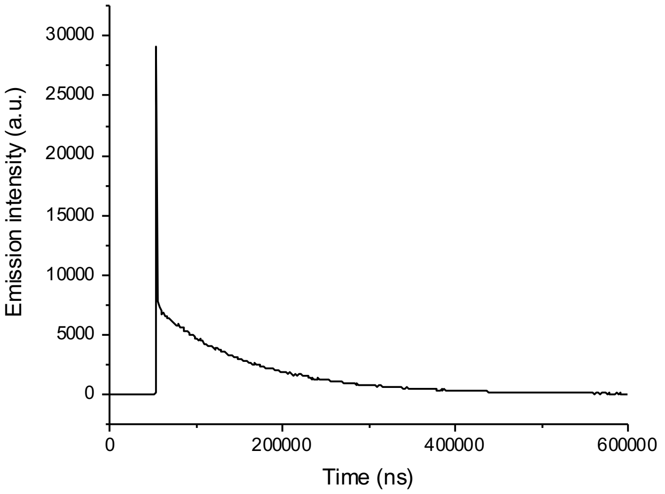

Figure S74. Kinetic trace of powdered **5** at 370 nm emission following 280 nm pulsed excitation, 295 K.

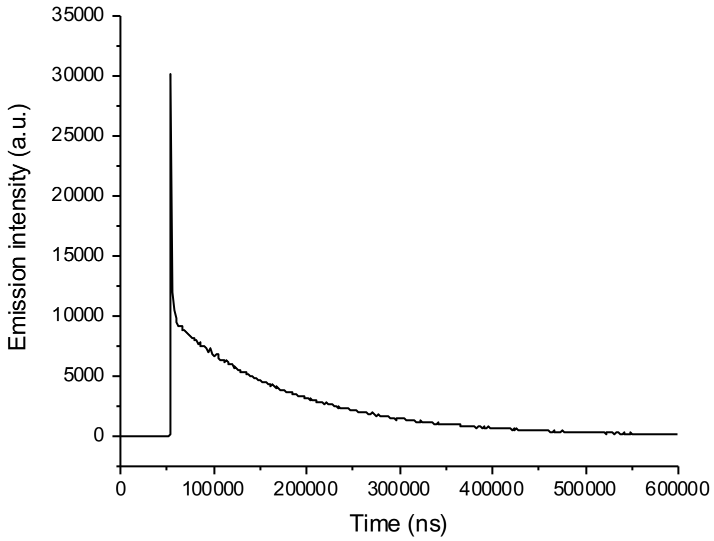

Figure S75. Kinetic trace of powdered **5** at 440 nm emission following 280 nm pulsed excitation, 295 K.

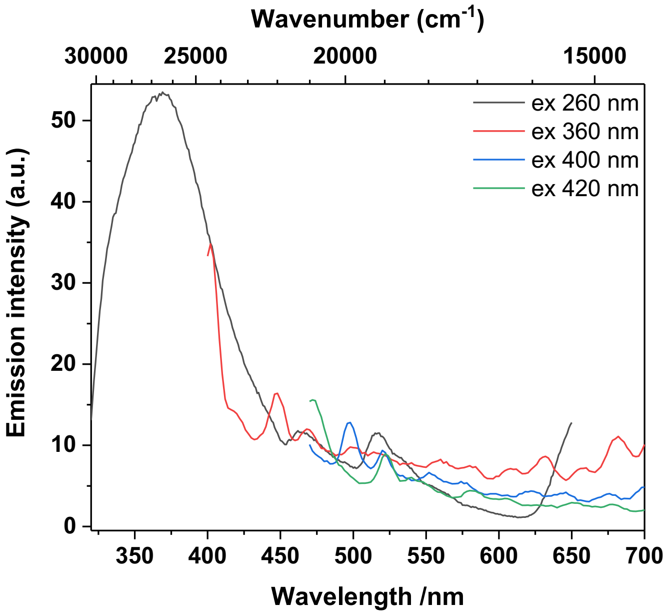

Figure S76. Emission spectra of **6** recorded at varying excitation wavelengths, 260, 360, 400 and 420 nm, 2-Me-thf, 77 K.

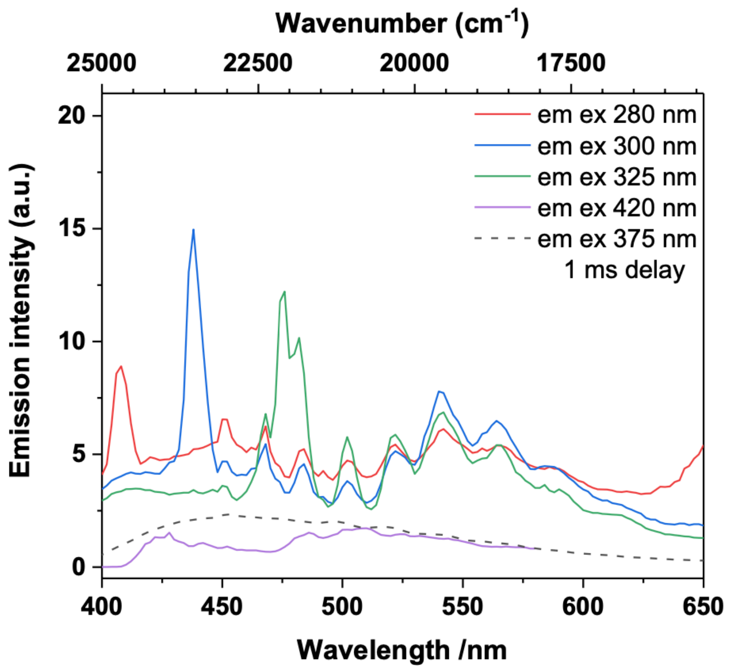

Figure S77. Emission spectra of **7** recorded at different excitation wavelengths (280, 300, 325, 375 and 420 nm), 2-Me-thf, 77 K and with a 1 millisecond (ms) time delay**.**

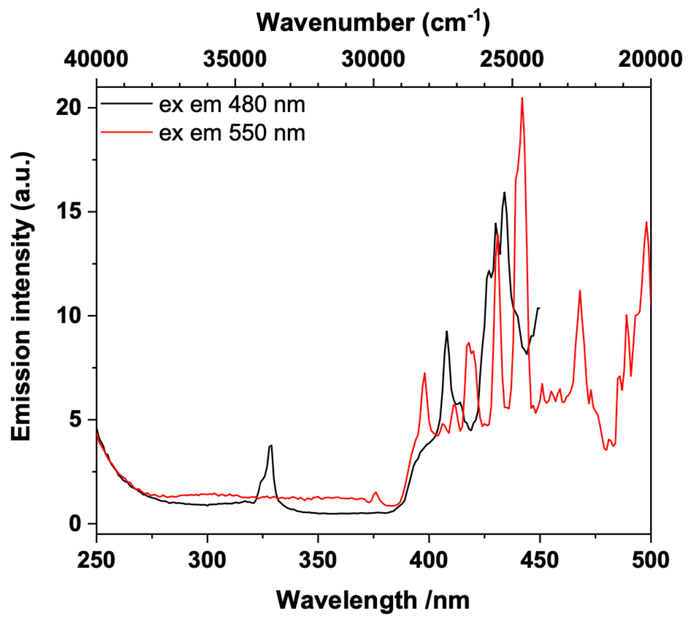

Figure S78. Excitation spectra of **7** recorded at the emission maxima 480 nm and 550 nm, 2-Me-thf, 77 K.

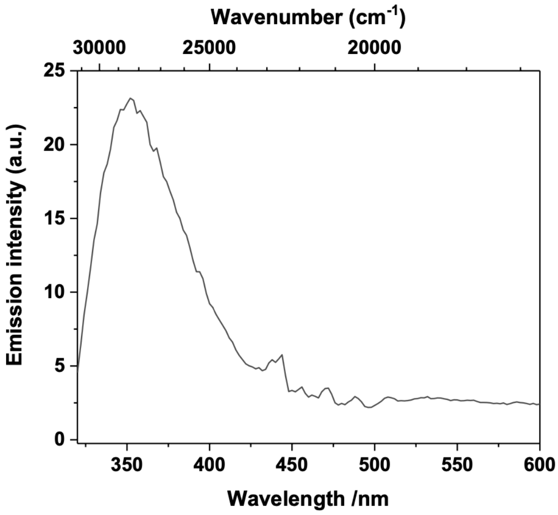

Figure S79. Emission spectrum of powdered **7** recorded at 295 K following 280 nm excitation.

7. Calculations

Calculations were performed using version 6.6 of the TURBOMOLE code,[10] employing the hybrid-GGA PBE0[11,12] exchange correlation functional and Ahlrichs-style basis sets of polarized triple-ξ quality.[13-16] The effects of THF solvation were included via the COSMO implicit solvent model.[17]

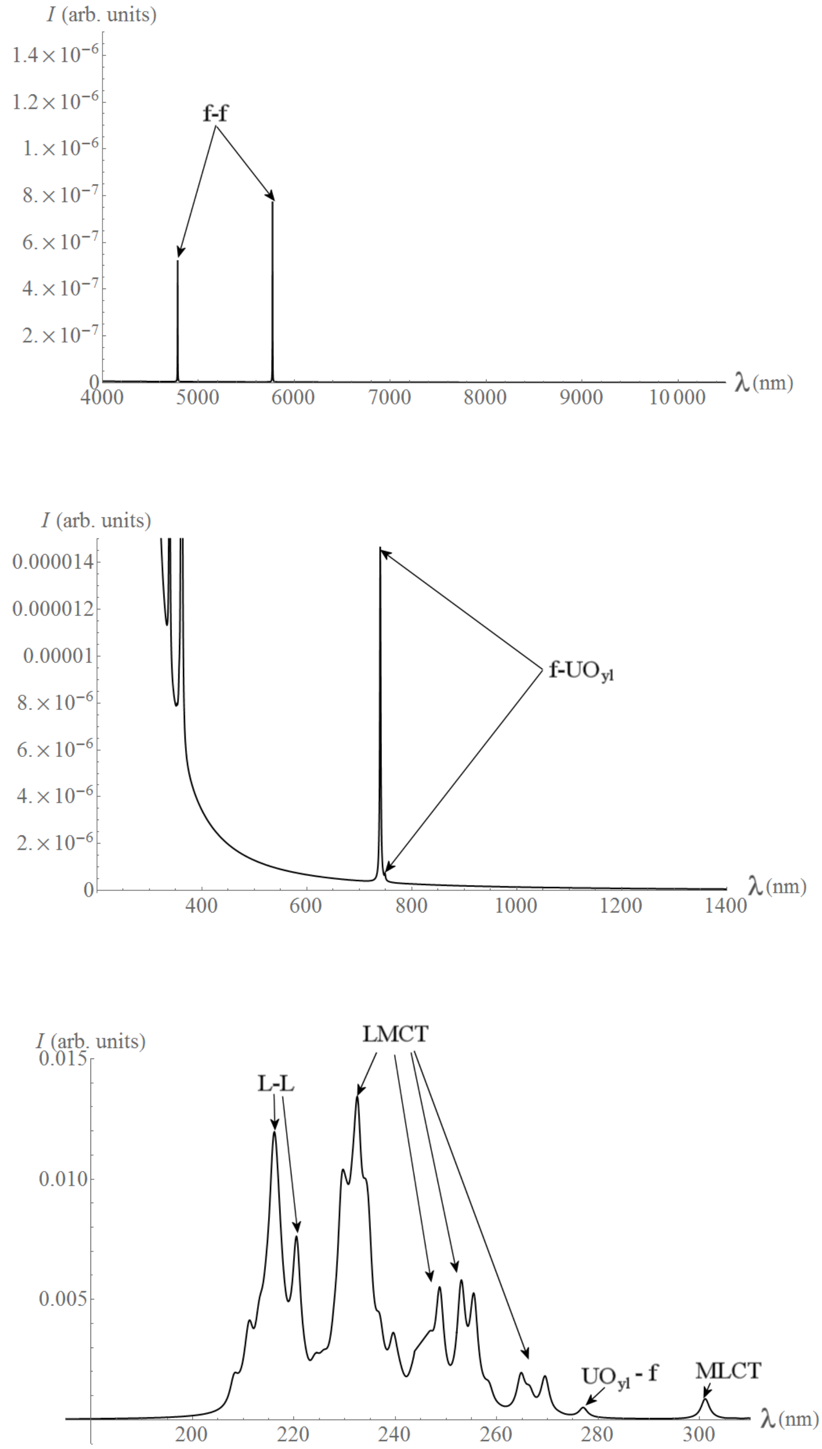

Figure S80. Calculated oscillator strengths and frequencies of excitations in the nIR (top), visible (middle) and UV (bottom) part of the electromagnetic spectrum in **3-crypt.**

Table S6. Character of all electronic transitions with appreciable oscillator strengths.

Transition wavelength (nm)	Transition Character	Oscillator Strength (*f*)
25040	5f – 5f	0.16×10^{-6}
5772	5f – 5f	0.25×10^{-5}
4782	5f – 5f	0.17×10^{-5}
748	5f – 5f, 5f – amide	0.70×10^{-6}
739	5f – amide	0.45×10^{-4}
361	5f – 5f, 5f – amide	0.17×10^{-3}
356	Uranyl-O-5f LMCT	0.26×10^{-5}
352	Uranyl-O-5f LMCT	0.57×10^{-6}
345	Uranyl-O-5f LMCT	0.30×10^{-7}
338	5f – 5f	0.54×10^{-4}
312	Uranyl-O-5f LMCT	0.45×10^{-5}
310	5f – amide	0.11×10^{-3}
307	5f – 5f	0.73×10^{-4}
302	Uranyl-O-5f LMCT	0.36×10^{-4}
301	5f – 5f, 5f – amide	0.26×10^{-2}

298	5f – 5f	0.11×10^{-4}
277	amide – 5f, UO2 – 5f	0.13×10^{-2}
270	5f – amide, amide – 5f, amide – 5f	0.49×10^{-2}
266	5f – amide, amide – 5f	0.23×10^{-2}
265	amide – 5f	0.47×10^{-2}
258	amide – 5f	0.38×10^{-3}
255	amide – 5f	0.13×10^{-1}
253	amide – 5f	0.15×10^{-1}
249	amide – amide, amide – 5f	0.13×10^{-1}
245	amide – amide, amide – 5f	0.12×10^{-1}
235	amide – amide, amide – 5f	0.13×10^{-1}
232	amide – 5f	0.94×10^{-2}
229	amide – 5f	0.19×10^{-1}
220	amide – amide	0.18×10^{-1}
216	amide – amide	0.12×10^{-1}
216	amide – amide	0.15×10^{-1}

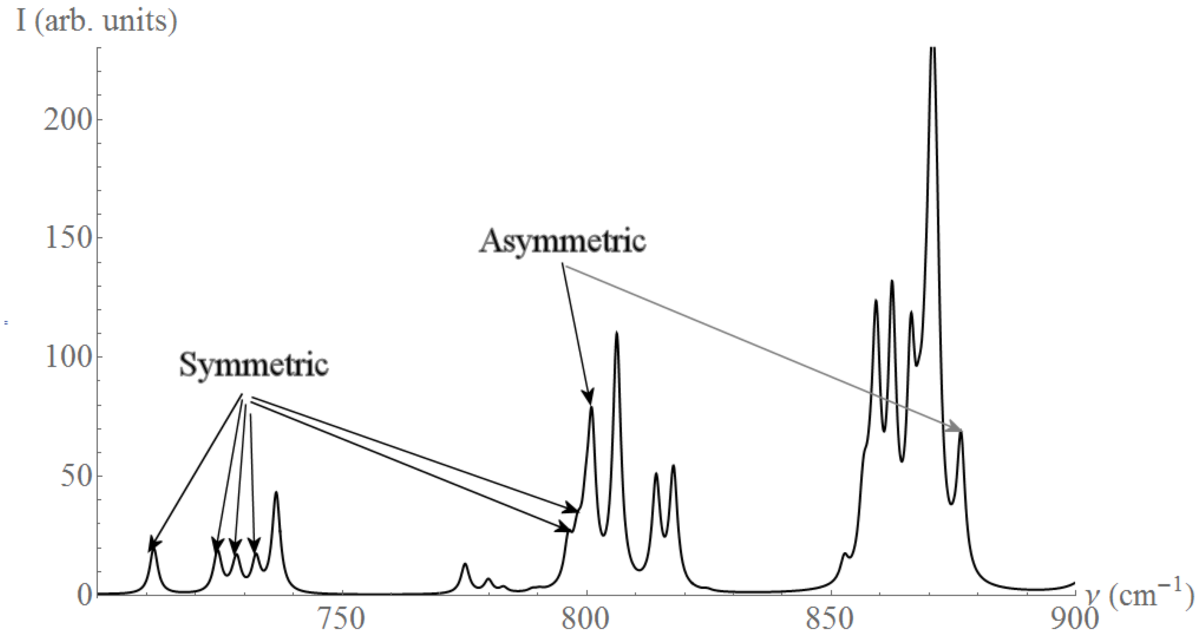

Figure S81. Calculated vibrational modes in **3-crypt**.

8. References

1 Cobb, P. J.; Moulding, D. J.; Ortu, F.; Randall, S.; Wooles, A. J.; Natrajan, L. S.; Liddle, S. T., Uranyl-tri-bis(silyl)amide Alkali Metal Contact and Separated Ion Pair Complexes. *Inorg. Chem.* **2018**, *57*, 6571-6583.

2 *CrysAlis* *Pro*, Agilent Technologies: Yarnton, England, 2010.

3 Sheldrick, G. M., A Short History of ShelX. *Acta Cryst. Sect. A* **2008**, *64*, 112-122.

4 Sheldrick, G. M., Crystal structure refinement with SHELXL. *Acta Cryst. Sect. C.* **2015**, *71*, 3-8.

5 a) Oszlanyi, G.; Suto, A., Ab initio structure solution by charge flipping. *Acta Cryst. Sect. A* **2004**, *60*, 134-141; b) Palatinus, L.; Chapuis, G., SUPERFLIP - A computer program for the solution of crystal structures by charge flipping in arbitrary dimensions. *J. Appl. Cryst.* **2007**, *40*, 786-790.

6 O. V. Dolomanov, O. V.; Bourhis, L. J.; Gildea, R. J.; Howard, J. A. K.; Puschmann, H., OLEX2: a complete structure solution, refinement and analysis program. *J. Appl. Cryst.* **2009**, *42*, 339-341.

7 Farrugia, L. J., WinGX and ORTEP for Windows: an update. *J. Appl. Cryst.* **2012**, *45*, 849-854.

8 *POV-Ray*, Persistence of Vision Raytracer Pty. Ltd.: Williamstown, Australia, 2004.

9 a) Denning, R. G., *Electronic structure and bonding in actinyl ions*. Springer: Berlin, 1992; Vol. 79; b) Denning, R. G.; Snellgrove, T. R.; Woodwark, D. R., The electronic structure of the uranyl ion. *Mol. Phys.* **2006**, 37 (4), 1109-

1143; c) Denning, R. G., Electronic Structure and Bonding in Actinyl Ions and their Analogs. *J. Phys. Chem. A* **2007**, *111* (20), 4125-4143.

10 Ahlrichs, R.; Armbruster, M. K.; Bär, M.; Baron, H.-P. TURBOMOLE 6.6. **2014**.

11 Perdew, J. P.; Burke, K.; Ernzerhof, M. Generalized Gradient Approximation Made Simple. *Phys. Rev. Lett.* **1996**, *77* (18), 3865–3868

12 Adamo, C.; Barone, V. Toward Reliable Density Functional Methods without Adjustable Parameters: The PBE0 Model. *J. Chem. Phys.* **1999**, *110* (13), 6158–6170

13 Schafer, A.; Huber, C.; Ahlrichs, R. Fully Optimized Contracted Gaussian Basis Sets of Triple Zeta Valence Quality for Atoms Li to Kr. *J. Chem. Phys.* **1994**, *100* (8), 5829.

14 Eichkorn, K.; Weigend, F.; Treutler, O.; Ahlrichs, R. Auxiliary Basis Sets for Main Row Atoms and Transition Metals and Their Use to Approximate Coulomb Potentials. *Theor. Chem. Acc.* **1997**, *97* (1–4), 119–124.

15 Küchle, W.; Dolg, M.; Stoll, H.; Preuss, H. Energy-Adjusted Pseudopotentials for the Actinides. Parameter Sets and Test Calculations for Thorium and Thorium Monoxide. *J. Chem. Phys.* **1994**, *100* (10), 7535.

16 Cao, X.; Dolg, M. Segmented Contraction Scheme for Small-Core Actinide Pseudopotential Basis Sets. *J. Mol. Struct. THEOCHEM* **2004**, *673* (1–3), 203–209.

17 Klamt, A.; Schüürmann, G. COSMO : A New Approach to Dielectric Screening in Solvents with Explicit Expressions for the Screening Energy and Its Gradient. *Perkins Trans.* **1993**, *2*, 799–805.
